# How “Green” is Your Functional Material? Assessing Environmental Impacts of Multi‐Step Synthetic Protocols in Material Sciences at Various Technology Levels

**DOI:** 10.1002/adma.74993

**Published:** 2026-09-24

**Authors:** Laura Plein, Alexander Schommer, Florian Dietrich, Moritz Gutsch, Heike Klesper, Kevin Schuller, Lars Jahnke, Jens Leker, Klaus Meerholz

**Affiliations:** ^1^ Department of Chemistry and Biochemistry University of Cologne Cologne Germany; ^2^ Institute of Business Chemistry University of Münster Münster Germany; ^3^ Helmholtz Institute Münster, IEK‐12 Forschungszentrum Jülich GmbH Münster Germany

**Keywords:** energy storage systems, material sciences, organic batteries, life cycle assessment, sustainability

## Abstract

Environmental awareness is increasing worldwide, driven by alarming developments such as the climate change. Life cycle assessment (LCA) provides a standardized framework to evaluate environmental impacts, for example in the material sciences. While LCAs are widely used for mature technologies, application in early‐stage research remains limited despite its high potential for sustainable improvements. This article introduces a broadly applicable formalism referred to as *Environmental Synthesis Radar (ESR)* to systematically evaluate the environmental impacts of multi‐step chemical synthesis protocols. ESR goes beyond the typical consideration of only one particular environmental aspect (e.g. “global warming”), but rather considers 18 midpoint impact categories (MICs), and thus yields a much more holistic and conclusive view. Importantly, the ESR formalism can be applied independently of the field of application of the material of interest, the material specifics, and the maturity of the technology. Exemplarily, we apply ESR to two cathode material classes for use in secondary batteries: an organic material based on tris‐(*p*‐methoxy)‐phenylamines (TPA) and an inorganic reference based on lithiated nickel‐manganese‐cobalt oxide (NMC). We obtain three important environmental metrics: (i) the per capita annual environmental output V_total_, a single number for comparison of different materials and contextualized interpretation; (ii) the environmental fingerprint V_EF_ for identification of the most impactful MICs, and (iii) the process radar V_PR_, which allows identification of environmentally critical synthesis steps and substances. ESR indicates that the impact of synthesizing 1 kg of the above materials is a factor of 3 (TPA) or 18 (NMC) below the global annual environmental emissions per capita, indicating that both are environmentally quite harmful. The laboratory synthesis of TPA is about one order of magnitude more harmful than the industrial production of NMC. Overall, ESR provides an easily accessible and very versatile tool for researchers—in academy as well as in industry—to evaluate and improve environmental performance of a material synthesis/production at an early stage when there is still lots of leverage. Importantly, it takes into account the holistic view of 18 MICs.

## Graphical abstract

Environmental Synthesis Radar (ESR) evaluates the environmental impact of (early‐stage) multi‐step chemical syntheses, providing a holistic view by using 18 midpoint impact categories. Unlike conventional life‐cycle assessment (LCA) approaches, ESR reveals three complementary metrics: overall impact V_total_, environmental hotspots V_EF_, and critical process steps V_PR_. By linking environmental impacts directly to individual process steps, ESR enables iterative optimization toward greener synthesis.

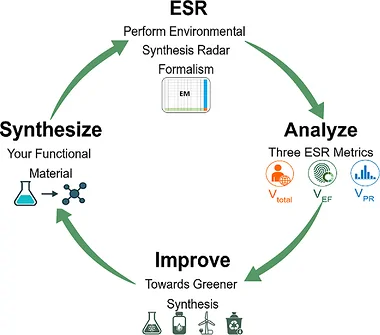

## 1 Introduction

Opto‐electronic and energy devices have recently entered global markets. A particularly successful case are organic semiconductors, such as those used in organic light‐emitting diodes (OLEDs), which are already widely applied in smartphones, tablets, and televisions, partially replacing earlier technologies such as liquid‐crystal displays (LCD) [1]. More recent, equally promising examples relate to capturing energy from abundant sources—such as in solar energy conversion [2, 3] or in thermoelectrics [4]—as well as in energy storage in primary (use once) or secondary (rechargeable) batteries [5] and flow batteries [6]. In many cases, established technologies are based on inorganic materials, while future alternatives might be based on organic materials which appear to be “greener” due to the fact that they are mostly composed of abundant elements (H, C, N, O, S, and others) [7, 8, 9, 10]. While all the “organic” technologies appear environmentally promising, there is currently no commonly accepted method to quantify the “greenness” of a material.

To reduce the negative environmental impact of laboratory syntheses or industrial production, the twelve (12) “Principles of Green Chemistry” have been introduced [11, 12]. In addition, a number of metrics‐based approaches have been proposed to assess the sustainability of multi‐step syntheses. Standard mass‐based metrics are the E‐factor (mass of waste/mass of product) [13], process mass intensity (PMI; total mass in a process/mass of product) [14, 15], solvent intensity (SI, total mass of solvent/mass of product) [16], atom economy (AE; molecular weight of product/total molecular weights of all reactants) [17], and carbon efficiency (CE, mass of carbon in product/total mass carbon in reactants) [18]. However, by reducing a synthesis or process to some mass, molar weight, or carbon content, the environmental “burden” of the individual compounds involved in the syntheses is completely neglected, even though they may have a very different level of toxicity. To assess and quantify the environmental impacts of materials syntheses, in particular at an early stage such as in an research laboratory, a broadly applicable validation method is needed. This would avoid falsely attributing superior or inferior sustainability levels to certain products, provide a more holistic view and can guide towards more sustainable syntheses [19].

Life cycle assessment (LCA), standardized under **I**nternational **O**rganization for **S**tandardization ISO 14040/14044 [20, 21], provides this comprehensive evaluation. However, LCAs are currently used mainly in industrial settings for mature technologies with a technology readiness level (TRL) above 8, where TRL is a metric to gauge the maturity of a given technology ranging from early‐stage research (TRL 1) to full commercial deployment (TRL 9). By contrast, LCAs are rarely applied for early‐stage technologies (TRL < 4), such as lab‐scale synthesis (TRL 1) or prototypes (TRL 2‐3) [22]. Such “early‐stage LCA” [23] face methodological challenges, including defining system boundaries, scaling laboratory data, and managing limited and uncertain data [24].

Material scientists in basic research are trying to develop next‐generation technologies and thus shape future industrial progress, while at the same time environmental and recycling concerns are growing due to limited resources and growing waste. Early environmental evaluation is crucial due to its significant leverage [25], and applying LCA in early research will help to identify potential environmental hotspots and support cost‐effective design improvements [26, 27] before established production setups constrain later development adoptions [28].

Among (industrial) organic syntheses, LCA of a few process routes of active pharmaceutical ingredients have been reported [29]. Recently, Carreira et al. studied the multi‐step synthesis of an antiviral drug Letermovir [30]. They considered three endpoint categories (“Human Health”, “Ecosystem Quality”, and “Natural Resources”) and optimized their reaction steps in terms of the environmental impact by a closed‐loop LCA approach. For benchmarking their optimizations, they focused on “global warming potential” (IPCC 2021 GWP100a). This allowed them to identify the (expected) hotspots: solvents, an organocatalyst, the palladium (Pd) contained in a transition‐metal catalyst, and lithium aluminum hydride (LiAlH_4_, LAH) as main contributors to the environmental impact in their study [30]. However, by focusing on only one environmental aspect (here GWP100) other impact categories were disregarded.

As evident from the above, there is an urgent need to develop a more comprehensive approach to judge the environmental impact of a material synthesis. Ultimately, we aim to motivate (material) chemists at research institutions—students and professors alike—to raise their environmental awareness, and to provide a potential synthetic pathway towards environmentally superior performance. This will allow them to assess the environmental signature of their novel materials at very early stage, and to reduce the environmental impacts of a given material synthesis.

In our work, we are introducing a novel, user‐friendly and broadly applicable LCA‐based methodology to assess the environmental burden of materials: the **“** *Environmental Synthesis Radar (ESR)* **.”** It considers the impact of all involved compounds individually, unlike mass‐based metrics mentioned above, which only consider the mass and not the “nature” of the chemical. Furthermore, it takes into account a broad range of 18 midpoint impact categories (MIC). We demonstrate how ESR employed at an early‐stage in the research process can guide the strategic design and synthesis of future materials in order to reduce environmental impacts.

As a test case for our first ESR study, we selected *cathode materials*, targeting their application in secondary batteries which are essential for the ongoing electrification of various sectors, including mobility, consumer electronics, stationary energy storage, and the Internet of Things (IoT) [31, 32]. Within the battery field, particular attention has been paid to sustainability assessments of next‐generation inorganic batteries. For instance, early‐stage sodium‐ion and lithium‐sulfur battery systems have shown a reduced environmental impact per 1 kWh of storage capacity at their current stage of development (low TLR level) when compared to state‐of‐the‐art lithium‐ion batteries [33, 34, 35, 36]. Furthermore, studies have compared battery cells based on lithium‐air, lithium‐sulfur, and sodium‐ion chemistries at various stages of technological maturity [37, 38, 39, 40].

For organic batteries, sustainability assessments are still scarce. Beyond studies focused on specific organic components—such as organic electrolytes for redox flow batteries [41]—Zhang et al. (2022) were the first to evaluate the environmental impacts of a flexible all‐organic battery [42, 43]. Using the model ILCD 2011 they identified “resource use”, “climate change”, “ozone depletion”, and “particulate matter formation” as the most critical environmental impact categories. Solvent use contributed most significantly to the environmental burden across these categories. They pointed out, that this impact could be mitigated by employing “greener” solvents or reducing solvent consumption [42].

In this paper, we benchmark organic cathodes synthesized at laboratory scale against an inorganic material produced on an industrial level. For both, we consider systematic variations of similar materials to illustrate and validate the ESR formalism and benchmark it against mass‐based metrics. Altogether, this will allow us to address the central question, “*How “Green” is your Functional Material?*”. Furthermore, we will put our results into a larger context, enabling us to estimate the contribution of energy storage in lithium ion batteries to the total global environmental emissions.

## 2 Methodology/“Experimental Section”

In this paper, we propose for the first time a logically consistent and broadly applicable formalism, referred to as *“Environmental Synthesis Radar (ESR)”* to access the environmental impact of the synthesis of a given material. In line with previous LCA and mass‐based metrics, we chose **1 kg** of the target material as the functional unit (FU; reference quantity). In the context of material research, this can be easily converted to the FU **1 mol** to enable substance‐specific comparability. The proposed formalism is independent of (i) the field of application (here, cathode materials, see below), (ii) the physical/chemical properties of the material under study (e.g., organic or inorganic, liquid or solid, etc.), and (iii) the maturity of a given technology (judged by the TRL, laboratory or industrial scale, respectively). ESR includes normalization of all MICs, which allows cumulating their impact to ultimately yield one characteristic metric, the so‐called “*total environmental impact indicator V* **_total_**”, which reflects the overall environmental burden of a target material relative to the output of a person during the year 2010 (the latest available dataset). Moreover, the ESR identifies the environmentally most critical processes of a multi‐step synthesis route (*process radar* **V_PR_**) as well as the environmentally most critical MICs of a given synthesis (*environmental fingerprint* **V_EF_**). Overall, the ESR formalism can guide the sustainable development of emerging material technologies by identifying critical environmental issues very early on.

### 2.1 Goal and Scope Definition

We have carried out an LCA in accordance with the standard ISO 14040/14044 [20, 21, 44]. It was conducted using the *openLCA 2.0* software [45] and *EcoInvent 3.9.1 (Cut‐off)* [46], a database suitable for the chemical laboratory and its processes. *ReCiPe 2016 v1.03, midpoint (E)* [47] (simply “ReCiPe” from now on) was used as life cycle impact assessment (LCIA) characterization model that converts life cycle inventory data—such as emissions and resource extractions—into environmental impact scores using scientifically derived characterization factors. The results are presented in the form of 18 midpoint impact categories (MICs; see Tables S1 and S2). ReCiPe quantifies impacts by applying substance‐specific parameters that reflect the relative contribution of each emitted or extracted substance to a given environmental mechanism, based on fate, exposure, and effect modeling [47]. It should be noted that most MICs address different environmental aspects and are, therefore, expressed in different physical units.

The ESR workflow or standard operation procedure (SOP) is shown in Figure 1, enabling simple transfer of the concept to other materials classes. We first examine the synthetic pathway (reaction scheme) of the target material, comprising n_P_ process steps (P). A generic step toward producing 1 kg of material is shown in Figure S1. Commercially available starting compounds, reagents (e.g., catalysts), and solvents (collectively termed “substances”) are used. Primary data were generated from process steps conducted in our laboratory. Since many commercial substances are not covered in the EcoInvent database, additional “upstream” synthesis steps (i.e., substrate synthesis from educts prior to our lab results; distinct from “retrosynthetic” or “reverse synthetic” approaches that trace a substrate back to an educt) were included to close data gaps (Figure 2). Data for steps not performed in our laboratory were taken from literature and patents.

**FIGURE 1:**
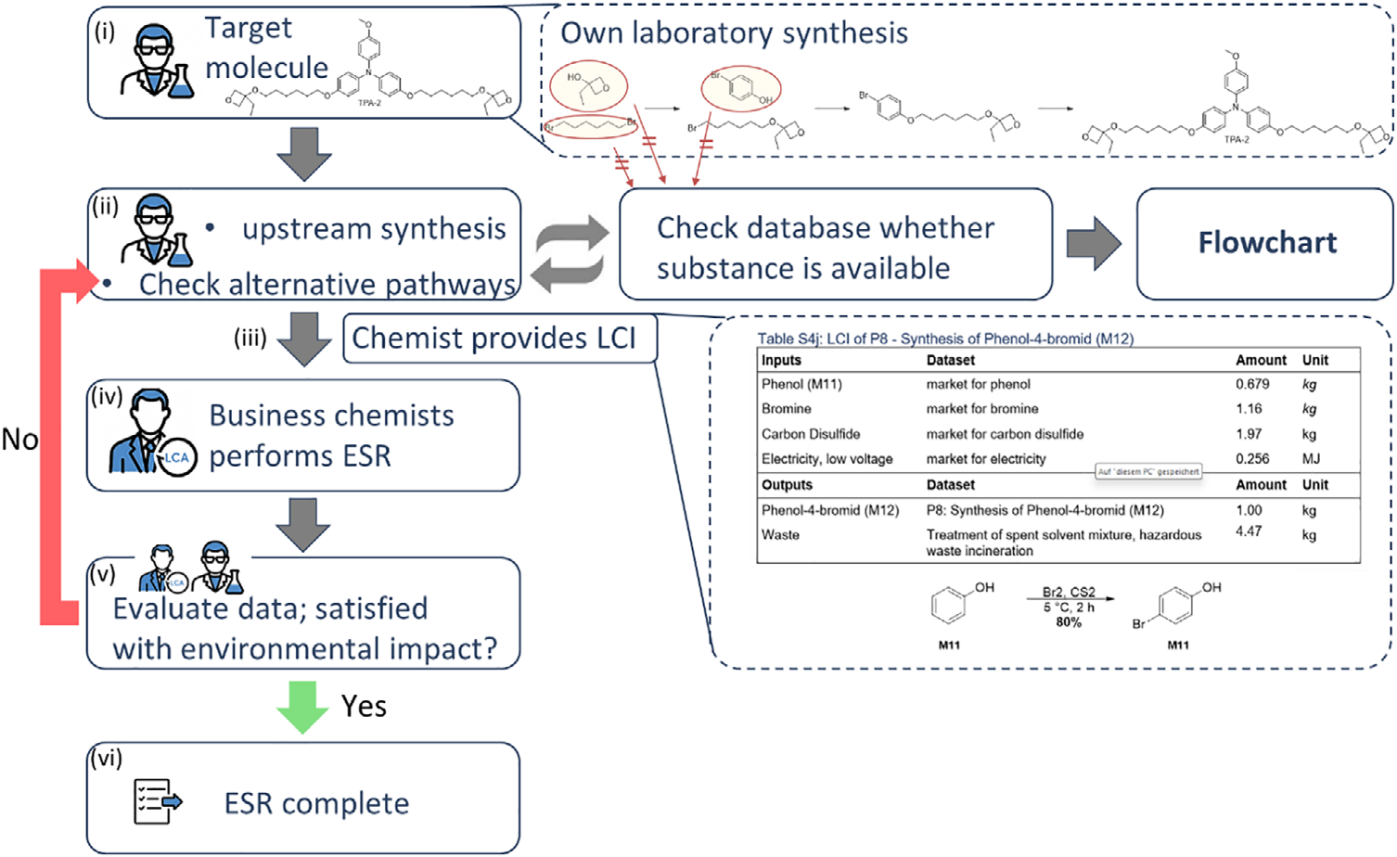
Graphical illustration of the workflow of the ESR‐formalism. (i) The synthetic chemist defines a target molecule synthesized in the own lab (here TPA‐2 as example), from a published work, or from industrial scale. (ii) The availability of the data from starting materials is checked in a suitable database. If such data is not available, an upstream synthesis is performed which can be illustrated in a flowchart (e.g. Figures S2–S4, S9, and S10). (iii) When all starting materials are found in the database, the chemist prepares the life cycle inventory (LCI) using the amounts of substances involved (e.g. Tables S4 and S13). (iv) The business chemist then performs an ESR (see Chapter 2.2). (v) The resulting data is evaluated. If the chemist is not satisfied with the environmental impact (No) and sees room for improvement, alternative pathways can be checked and reaction steps optimized start again from (ii). (vi) Alternatively, if the environmental impact is satisfactory (yes), the ESR is complete.

**FIGURE 2:**
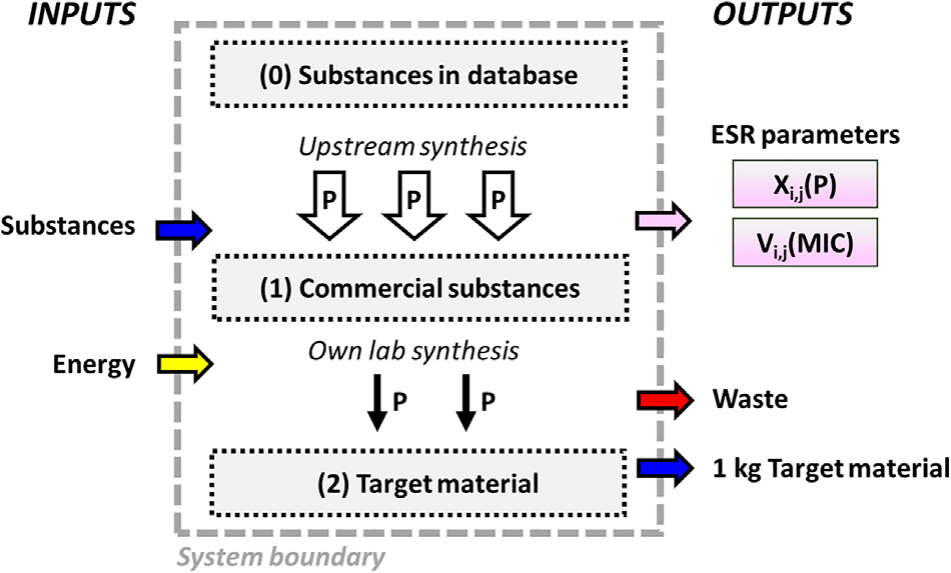
Schematic illustration of a generic multi‐step synthesis: It includes the starting substances provided by the database EcoInvent (0). A number of upstream synthetic steps (white down arrows) lead to the commercial substances (1) used in our lab synthesis (black down arrows) towards the target material (2). The system boundary is shown as a box using a grey dashed line. Inputs including various substances (blue arrow) and energy (yellow) point into the system (left), whereas outputs including 1 kg of the target material (blue) and the cumulated waste (red) point towards the outside (right). Further outputs are the process‐specific quantities X_i,j_(P) and V_i,j_(MIC) required for the ESR formalism (pink), see Figure 3.

Within the defined system boundary, the complete sequence of processes is included. The input stream consists of the electricity/energy required for each process step and all used substances. The output stream consists of 1 kg of the target material as well as the cumulated waste. The herein generated ESR parameters X_i,j_(P) and V_i,j_(MIC) (output) are entered as input for the ESR formalism in Figure 3.

**FIGURE 3:**
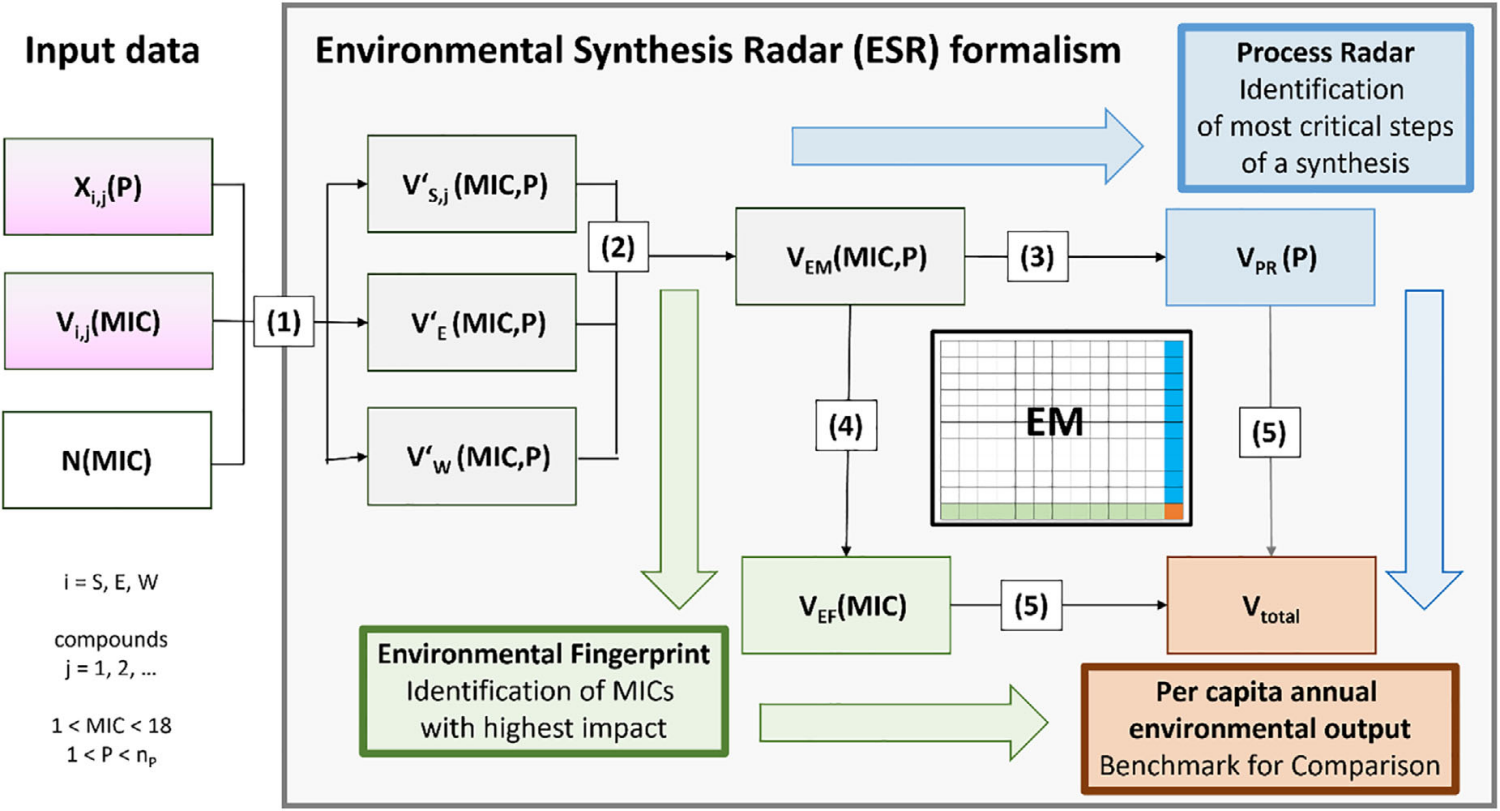
Illustration of the ESR‐formalism. The input streams are the output data from Figure 2 (X_i,j_(P) and V_i,j_(MIC), both for 1 kg of product in each process step P) and the normalization score N(MIC). The numbers (1)–(5) refer to the equations in the main text. The numerators “i”, “j”, “EM”, “PR”, and “EF” are explained in the main text. The suitable range of the variables “MIC” and “P” is indicated in the lower left corner.

### 2.2 Life Cycle Inventory and Life Cycle Impact Assessment

Our novel, generically applicable ESR formalism allows to access the environmental impact of the synthesis of a given (generic) material in five steps as illustrated in Figure 3. Considering the system boundaries as well as all defined inputs and outputs, this study constitutes a “cradle‐to‐gate” LCA. We first introduce the mathematical model, before we put the results into context.

**STEP 1**: We start by determining the environmental impact of each substance (S) that is used in the syntheses, the specific energy consumption (E), and waste generation (W) involved in each individual process step (P) for all MICs (see Figure 2). To compromise between data availability, reasonable time required to calculate the impacts and accuracy of results, LCAs commonly make use of assumptions. Details of the used methodology regarding substance and energy inputs, as well as waste output can be found in Chapter S1 of the Supporting Information. The corresponding quantities of in‐ and outputs are listed in Tables S4a—S4s.

Ultimately, this yields *item‐specific indicator* ***V*** *alues* V_i,j_(MIC, P), where the index “i” denotes the type of flow (S, E, or W), and “j” the numerator for substances S involved in a given process step P. The V_i,j_(MIC) values are retrieved in a standardized form from the EcoInvent database for 1 kg substance or 1 MJ energy (Table S5). The respective quantities X_i,j_(P) involved in a process step P were used according to the same principle.

Since a direct comparison between the MICs is not feasible due to their different physical units (Table S1), we normalized the original EcoInvent data by the official normalization scores for each midpoint impact category N(MIC) [48] (Table S6), which quantify the worldwide annual environmental emissions for the different MICs divided by the world's population in 2010 Equation (1).

(1a) $${V^{\prime }}_{S,j}\left(MIC,P\right)=\frac{V_{S,j}\left(MIC\right)\cdot X_{S,j}\left(P\right)}{N\left(MIC\right)}\;$$

(1b) $${V^{\prime }}_{E}\left(MIC,P\right)=\frac{V_{E}\left(MIC\right)\cdot X_{E}\left(P\right)}{N\left(MIC\right)}\;$$

(1c) $${V^{\prime }}_{W}\left(MIC,P\right)=\frac{V_{W}\left(MIC\right)\cdot X_{W}\left(P\right)}{N\left(MIC\right)}\;$$

**STEP 2**: Next, we calculated the ***E*** *nvironmental* ***M*** *atrix (EM) indicator values* V_EM_(MIC,P) by summarizing the dimensionless standardized specific indicator values V’_i,j_(MIC,P) (Equation 2).

(2) $$V_{EM}\left(MIC,P\right)=\sum_{nS}{V^{\prime }}_{S,j}\left(MIC,P\right)+{V^{\prime }}_{E}\left(MIC,P\right)+{V^{\prime }}_{W}\left(MIC,P\right)$$

with n_S_ being the number of substances involved in process step P.

**STEP 3**: By summarizing the V_EM_(MIC,P) across all MICs (i.e., for each process step P, each row of EM), we obtain the ***P*** *rocess* ***R*** *adar (PR) indicator values* V_PR_(P) (Equation (3), colored in blue in Figure 3).

(3) $$V_{\;PR}\left(P\right)=\sum_{18}V_{EM}\left(MIC,P\right)$$

where 18 is the number of the different MICs (defined by ReCiPe; see Table S1).

**STEP (4)**: Alternatively, by summarizing the V_EM_(MIC,P) over all processes P (i.e., for each column of the EM, each MIC), we obtain the ***E*** *nvironmental* ***F*** *ingerprint* (EF) *indicator values* V_EF_(MIC) (Equation (4), colored in green in Figure 3).

(4) $$V_{EF}\left(MIC\right)=\sum_{n_{P}}V_{EM}\left(MIC,P\right)$$

with n_P_ being the total number of process steps P in the synthesis of the target material.

**STEP (** **5** **)**: Finally, by summarizing either the V_PR_(P) for all processes P or the V_EF_(MIC) for all MICs, we obtain the *total environmental indicator value* V_total,wt_ (Equation (5), colored in orange in Figure 3.) for the synthesis of 1 kg target material.

(5)

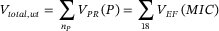




Using the molecular weight M_w_ of the target material V_total,wt_ can easily be converted from the FU 1 kg to the FU 1 mol yielding V_total,mol_ by Equation (6).

(6)

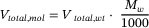




For simplicity wherever V_total_ is stated, both metrics V_total,wt_ and V_total,mol_ are meant, depending on the context. Please note, that V_total,wt_ is given in units of [kg^−1^], while V_total,mol_ is given in units of [mol^−1^].

In the following, we will put the above procedure into perspective. Importantly, it should be noted, that all indicator values (V’_i,j_, V_EM_, V_PR_, V_EF_ and V_total_) are normalized values, resulting from STEP 1. Therefore, they provide information about the impact of the synthesis of 1 kg (1 mol, respectively) of the target material relative to the amount of emissions caused by an average person in the world per MIC in 2010 (the latest available dataset). By referencing to a global benchmark, this approach yields a single‐number indicator that enables a cross‐category comparison of the environmental impacts of the individual process steps [49], which would not be possible otherwise.

Further, the environmental matrix (EM), a tabular representation of all MICs and Ps, provides a detailed overview about the environmental burden **V_EM_(MIC, P)** in a specific MIC caused by a specific process step P. All entries are dimensionless due to the normalization. The process radar **V_PR_(P)** is a measure for the overall environmental burden, that a process step P causes and can be used to explore the environmentally most critical processes of a synthesis route. Please note, that this considers all MICs as equally “important” regarding their environmental impact.

The environmental fingerprint **V_EF_(MIC)** is a measure for the environmental burden, that is accumulated in one specific MIC and can be used to identify the environmentally most critical MICs of a given synthesis. A value V_EF_(MIC) = 1 indicates that the synthesis of 1 kg of the material causes the same amount of emissions in the corresponding MIC as an average person in the world does per year. A much lower V_EF_(MIC) (<<1) indicates that the environmental burden of this MIC of the synthesis under consideration is relatively minor in a global context. Vice versa, V_EF_(MIC) (>>1) signals a high environmental impact of the synthesis in this MIC when compared to the annual environmental emissions per capita. Normalization, thus, facilitates contextualized interpretation and enhances comparability of results across different impact categories. However, it should be noted that MICs that already exhibit high global annual levels—such as “global warming” or “particulate matter formation”—are therefore less prominent in our standardized relative assessment, unless the analyzed synthesis exceeds what is currently observed at the global level and thus taken as ‘normal’ (without judgement). Our ESR approach thereby shifts attention to those impact categories where the synthesis imposes high environmental burdens relative to the global impact profile, highlighting the categories of greatest concern.

Finally, the total environmental indicators V_total,wt_ and V_total,mol_ reflect the overall environmental impact that the synthesis of 1 kg or 1 mol of a specific material causes, respectively. However, being one single metric, these values not only provide a classification of the target material to the annual environmental emissions per capita, but also offer the ability to easily compare target materials with each other. An in‐depth interpretation of V_total_ would require expert‐based weighting of the different MICs, because in our treatment all MICs have been treated equally impactful (Equation (3), prefactor 1 omitted). To include differences in impact by the MICs was considered beyond the scope of this study, since ReCiPe does not provide weighting factors that could be applied [47, 50]. Nonetheless, a value V_total,wt_ = 18 indicates that the synthesis of 1 kg of the material causes the same amount of emissions as an average person in the world does, but the individual contribution of the MICs might differ from 1.

Finally, we would like to emphasize, that none of the indicator values considers experimental performance parameters specific to a given material or application.

## 3 Selection of Test Cases for Application of the ESR‐Formalism

As a test case for our first ESR study, we selected *cathode materials*, targeting their application in secondary batteries. Inorganic Lithium‐Ion Batteries (LIB) are widely used in everyday life and find application in electric vehicles (EVs) and portable devices such as smartphones. Since their commercialization in 1991 [51, 52], significant progress in terms of sustainability and cost efficiency were made resulting in an increasing demand on the global market [53, 54]. With regard to cathode materials, the commercialization of **LiNi_x_Mn_y_Co_1‐x‐y_O_2_** layered oxides (**NMCxyz**, with x, y, and z representing the molar ratio of the three metals in the mixed oxide) is at a mature stage, due to their cost and enhanced specific capacity and thermal/mechanical stability [55, 56]. More recently, organic battery materials entered the scene, having important experimental advantages compared to inorganic batteries, for example they facilitate accelerated charge discharge cycles (CDC), and demonstrate remarkable cycling stabilities [57, 58, 59, 60, 61]. On the other hand, the capacity is often much lower than for inorganic analogues due to the large molecular weight. Many organic material classes have been reported as electrode materials [32, 62, 63, 64, 65, 66, 67, 68]. However, for each case there are technical PROs and CONs, and a clear decision regarding their future use cannot yet be taken. For this pilot study, we selected derivatives of tris‐(*p*‐methoxy)‐triphenylamine (**TPA**) developed in our lab.

Both material classes, **NMC** and **TPA,** feature similar cell voltages relative to Li/Li^+^ (3.5 V and 3.7 V, respectively; Table 1) and are obtained by multi‐step synthesis protocols. Further, systematic variations can easily be made (composition, number of reactive groups). Ultimately, this allows us to compare the synthesis of an organic (in solution) and an inorganic cathode material (by thermal conversion) on a laboratory scale (research environment) and on an industrial level.

**TABLE 1:** Technical metrics for TPA‐ and NMC811‐based cathodes synthesized on the laboratory scale.

Materials	TPA‐0	TPA‐1	TPA‐2	NMC811
Molecular formular	C_21_H_21_O_3_N	C_32_H_41_O_5_N	C_43_H_61_O_7_N	Li(Ni_0.8_Mn_0.1_ Co_0.1_)O_2_
Molecular weight per repeat unit [g mol^−1^]	335.40	519.68	703.96	97.28
Mol per kg	2.98	1.92	1.42	10.28
Theoretical specific capacity [mAh g^−1^]	76	52	38	275
Practical specific capacity [mAh g^−1^]	n.a.^a^	45 [78]	37 [78]	175‐190 [81]
Practical voltage vs. Li/Li^+^ [V]	n.a.^a^	3.5	3.5	3.7 [81]
Theoretical energy density of cathode material [Wh kg^−1^]	n.a.^a^	182	133	1018
Capacity retention # cycles (C‐rate)	n.a.^a^	n.a.^a^	82% 10,000 (6C) [78]	80% 200 (3C) [82] 2,500 (0.5C) [82]
V_total,wt_ [kg^−1^]	7.3 (1.25*)	7.3	5.2	1.0 (0.5)^b^
V_total,mol_ [mol^−1^]	2.4 (Pd‐cat.)	3.8 (Pd‐cat.)	3.7 (Pd‐cat.)	0.10 (0.05)^b^
V_total,mol_^*^ [mol^−1^]	0.42 (Cu‐cat.)	2.1^c^ (Cu‐cat.)	2.8^c^ (Cu‐cat.)	n.a.

^a^ Not applicable due to (partial) solubility.

^b^ Industrial production.

^c^ Numbers based on BBA not on ESR.

*with Cu‐catalyst.

### 3.1 Test Case 1: Organic Cathode Material TPA

Organic battery materials contain a redox‐active core unit and are often polymerized or even crosslinked to ensure that both, the neutral and the charged state are insoluble in the electrolyte, preventing leaching during repeated CDC [63, 69, 70, 71, 72]. However, the synthetic introduction of (redox‐inactive) polymerizable groups not only requires additional synthesis steps, but also increases the molecular weight, thereby reducing the theoretical redox capacity (charge per mass; mAh g^−1^) as well as the energy density (Wh kg^−1^), which in turn alters the environmental impact.

For this pilot study, we selected crosslinkable derivatives of tris‐(*p*‐methoxy)‐triphenylamine (**TPA,** Scheme 1). Initially, this material class was synthesized in our laboratory for application as hole‐transport material in the field of organic electronics, for example in organic light‐emitting diodes (OLEDs) [73, 74, 75, 76]. However, these **TPA**s also exhibit remarkable advantages as a cathode material in secondary batteries [77, 78]. For the purpose of comparison and validation of the ESR formalism, three **TPA**‐derivatives (Scheme 1) will be considered in this work, featuring varying numbers of side groups (pink, also referred to as “arm”) bearing an oxetane ring as reactive unit, which can be polymerized via cationic ring‐opening polymerization [73, 76]: **TPA‐0** without a reactive group is considered as reference to differentiate the environmental impact of the redox‐active core unit (green) from the reactive side groups. Note, that **TPA‐0** would be unsuitable as a battery material because it is soluble in most electrolytes and would dissolve from the current collector. **TPA‐1** features one reactive oxetane unit; polymerization would lead to a linear side‐chain polymer, which is still soluble in the electrolyte. Finally, **TPA‐2** features two reactive oxetane units and can be turned into a polymer network which is insoluble in all solvents [73, 76]. Table 1 compares some key technical metrics of the cathode materials under consideration.

**SCHEME 1:**
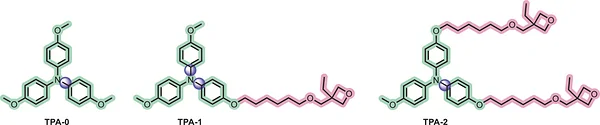
Chemical structures of **TPA‐0**, **TPA‐1** and **TPA‐2**. We color‐coded the redox‐active core (green), the polymerizable functional groups with linker “arm” (pink), and the C─N bond formed using a catalyst (blue circles).

The actual experimental synthesis performed in the laboratory for **TPA‐0** can be accomplished in a single step (step P15, red frame, in Scheme 2) starting from the commercially available precursor (here M13 and M14), as reported by Stephens et al. [79] (Buchwald‐Hartwig amination, BHA, Pd‐catalyzed) or by Chaudhari et al. [80] (Ullmann reaction, Cu‐catalyzed). In analogy, three‐step synthesis protocols were developed in our research group for **TPA‐1** and **TPA‐2** (see Schemes S1–S4); here, the commercially available starting materials M6, M9, M12–M14, and M17 were used. Since these commercial starting materials are not included in the EcoInvent database, the upstream‐synthetic approach described above (Figure 2) was applied iteratively until suitable starting materials could be found in the database. This enabled the determination of the corresponding environmental impacts.

**SCHEME 2:**
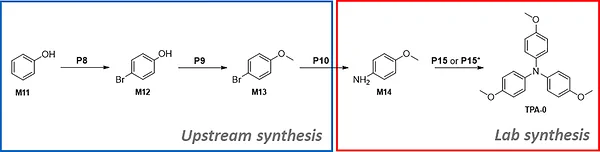
Reaction scheme for the synthesis of **TPA‐0** starting from phenol (M11), including **P8** Br_2_, CS_2_, 5°C for 2 h, distillation, 80%; **P9** Dimethyl carbonate, Acetonitrile, 160°C for 2 h, MW‐reactor, 100%; P10 Cu, NH_3_, water, autoclave 200°C for 2 h, 91%; **P15** BHA: Pd‐cat., P‐ligand, two equivalents of M13, NaO^t^Bu, toluene, 100°C for 15 h, 95% or P15* Ullmann reaction: CuI, P‐ligand, two equivalents of M13, KO^t^Bu, toluene, 135°C for 10.5 h, 87%. Process steps **P8** – **P10** are upstream synthesis steps, and P15/P15* is a lab synthesis step.

A detailed overview of the individual process steps P of the three **TPA** syntheses (**P1**–**P15**, including both, the experimental steps performed in our laboratory and the upstream‐synthetic steps from the literature, as well as all LCA‐relevant data (reagents, solvents, and reaction conditions) are provided in Table S3. Flowcharts showing the complete synthesis route are provided in Figures S2–S4, respectively.

#### 3.1.1 Application of the ESR Formalism to the TPA Synthesis

We first apply the ESR formalism to the synthesis of the three **TPAs**, yielding total environmental impact values V_total,wt_ for 1 kg of each material: it appears that **TPA‐0** and **TPA**‐**1** have a similar impact (ca. 7.3 kg^−1^; see Table 1), while **TPA‐2** appears less harmful (ca. 5.2 kg^−1^). By categorizing the impacts into the categories “reagents”, “solvents”, “energy”, and “waste” (Table S11 and Figure S5), it becomes obvious that the largest impact originates from the harmful reagents used in the synthesis (∼ 65%–80%; details about what specifically causes the high impacts will be discussed using V_PR_(P), see below), followed by solvents (∼10%–20%), while the energy consumption (to heat or cool the reaction vessels) and waste disposal play a minor role only (both below 10%).

The environmental fingerprint **V_EF_(MIC)** identifies critical MICs, whereas values larger than unity indicate environmental impacts exceeding the annual per‐capita level and, thus, require further discussion. In our case, “Human Toxicity—Cancer” (HTPc; MIC 9) was identified as most critical (Figure 4 and Tables S7–S9), being responsible for about 80% of the total environmental burden of all three **TPA** syntheses. This outcome results primarily from the fact that most of the substances used in our syntheses inherit a high cancerogenic potential [83]. For comparison, the LCA performed by Zhang et al. [42, 84]. on a fully‐organic battery based on conductive redox polymers [43] identified the impact categories “climate change” (in our analysis: GWP100, MIC 2), “ozone depletion” (ODP infinite, MIC 14), and “particulate matter formation” (PMFP, MIC 15) as environmentally most relevant [42, 84]. In our study these categories do not stand out, even have a minor contribution. This discrepancy results from the fact that Zhang et al. quantified the environmental impacts based on the absolute values per functional unit, whereas our approach uses normalization of the absolute values to the global per capita consumption and production levels. Naturally, also a difference due to the different chemistries can be expected. Carreira et al. focused only on one MIC, “climate change”, for the synthesis optimization of *Letermovir*. They identified hotspots such as solvents (THF, MTBE, DCM), reagents (Pd‐catalyst, organocatalysts, LiAlH_4_ and *m*CPBA) and yields. Additionally, they used the three Endpoint impact categories (EIC) defined in ReCiPe 2016 to broaden their scope. However, with addressing EICs no particular reagents/solvents can be identified as environmentally (most) harmful. We emphasize, that the proportional ranking of the four syntheses depends on the MIC chosen for comparison.

**FIGURE 4:**
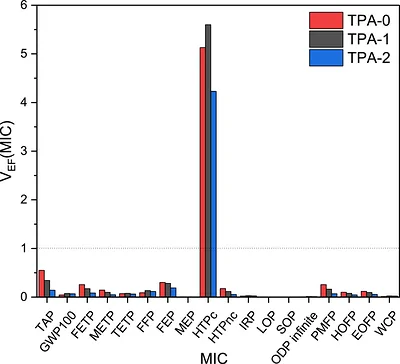
Environmental fingerprints of the syntheses towards **TPA‐0** (red), **TPA‐1** (black), and **TPA‐2** (blue). For abbreviations, see Table S1.

Next, we use the process radar (PR) indicator values **V_PR_(P)** to identify the environmentally most critical processes steps of the synthesis of the respective **TPA** derivatives. We will only discuss processes exceeding a threshold, defined as the total impact V_total,wt_ for 1 kg of the material divided by the number of process steps (average impact). Figure 5 identifies three environmental “hotspots”: the processes **P5**, **P6**, and **P12** ‐ **P15** surpass the respective thresholds, that is, have an above‐average, critical environmental impact. Overall, these processes collectively account for 75%–85% of the total environmental impacts for the laboratory‐based **TPA** synthesis. **P5** (required for **TPA‐1** and **TPA‐2**) concerns the reduction of adipic acid (M7) to 1,6‐hexanediol (M8) using LAH (LiAlH_4_) as the reducing agent. LAH contributes substantially to the overall environmental burden due to the use of lithium as also noted by Carreira et al. [30]. **P6** (required for **TPA‐2**) concerns the bromination of 1,6‐hexanediol (M8) towards 1,6‐dibromohexane (M9). The involvement of bromine causes the elevated impact, as also noted by Zhang et al. [42]. Finally, the process steps **P12** ‐ **P15**, which are specific to only one of the three **TPA** versions, respectively, are Pd‐catalyzed C─N coupling reactions. As a precious metal, Pd exhibits a particularly high environmental burden. Other LCAs also identified the high environmental impact of brominated educts and Pd‐catalysts in C─C coupling reactions [85, 86]. We emphasize that these environmental “hotspots” are expected, but it is important to demonstrate that ESR is able to identify and even quantify them. LAH as well as metal‐catalysts had also been reported as crucial by Carreira et al. [30]. In the following, we will discuss possible improvements.

**FIGURE 5:**
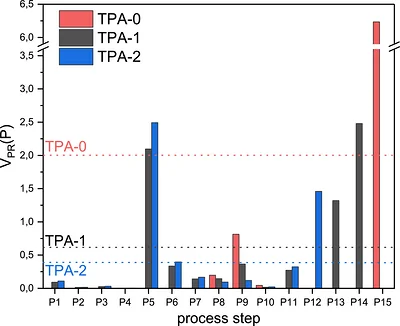
Process radar of **TPA‐0** (red), **TPA‐1** (black), and **TPA‐2** (blue). The cutoff threshold (see main text) is shown as dashed line in the respective color. For respective process steps, see Table S3.

#### 3.1.2 Improvements of the TPA Synthesis Route

The insights gained by **V_PR_(P)** enable an iterative improvement process aimed at reducing the overall impact of the synthesis. In general, to optimize a given synthesis route, there are some general concepts to be considered: **(i)** improving the most critical process step(s) by replacing the most critical substance(s) with less critical ones, as will be discussed below; **(ii)** increasing the process yields, especially for processes situated late in the process chain, which leverage the impact of all prior processes; **(iii)** reducing the amount of catalyst and/or its recovery, for example by using an immobilized catalyst, which can be reused many times, and **(iv)** reducing the impact of solvents by minimizing their use in large excess, recycling of solvents, and exploring the substitution with greener alternatives [84, 87, 88]. On the industrial level, various scaling effects can further improve the environmental impacts. Examples are **(v)** optimized reaction timing and **(vi)** heat recovery leading to reduced energy demand. **(vii)** Furthermore, stoichiometric by‐products can be further processed in an industrial purpose. This would be treated as environmental “income” (positive impact) as opposed to “burden” (negative impact).

We now address improvements specific to the **TPA** synthesis suggested by the ESR formalism. As already stated above (point (i)), the most critical process steps can be optimized by replacing the most critical substances or processes with less critical ones. When considering the details of **P5**, requiring LAH, to the best of our knowledge every alternative reducing agent with a more favorable environmental impact (e.g. TiCl_4_/BH_3_NH_3,_) [89] would reduce the yield (only 55% yield compared to 78% using LAH, Table S3). Please note, that generally a lower yield impacts on all prior process steps, because all “early” quantities have to be increased [90]. Most probably, this will outbalance the environmental gain achieved by the “better” (i.e., less harmful) reducing agent. In **P6** a halogen, more specifically bromine, is essential for the further synthesis and can, therefore, not be avoided. To reduce the environmental impact, Br_2_ could be replaced by Cl_2,_ [42] again most probably reducing the yields; in addition, this substitution might not be compatible with the following reaction(s). Finally, for the Pd‐catalyzed BHA‐reactions (**P12‐P15**), the catalyst quantity varies by a factor of four across the individual steps, that is, 0.5 mol% Pd‐catalyst for **P13** and 2.0 mol% Pd‐catalyst for **P15** (Table S3). We hypothesize that these differences are not truly necessitated by the synthesis, but are rather the chemist's choice. This is quite common, because in laboratory research the amounts of a chemical (here Pd) employed are commonly not adjusted having in mind the environmental impact, but are rather based on precedents in the literature, such as the original C─N coupling protocol (2.0 mol%) reported by Hartwig et al. [91] to which Stephens et al. [79] (**P15**) referred to.

Therefore, starting from the original synthesis protocols (referred to as **case A**) we propose two *“*in silico*”* ESR‐based improvement steps (theoretical, not performed in the laboratory). First, we suggest using an equal amount of Pd‐catalyst of 0.5 mol% (the smallest value used in the literature we used) for all C─N coupling reactions **P12**‐**P15**, assuming the same yields as in case A (**case B**). Second, we propose to replace the Pd‐catalyzed BHA with an Ullmann‐type reaction, employing 3.5 mol% of a copper‐based catalyst as reported for the synthesis of **TPA‐0** [80] (**P15**) (**case C**). Here, we assume the same yield reported in the literature for P15 also for **P12**‐**P14**. Since in the scientific literature the amounts of catalyst are given in units of [%mol], for the further discussion we convert V_total,wt_ used so far to V_total,mol_ Equation (6).

The original synthesis protocols (**case A**) yielded V_total,mol_ = 2.4 mol^−1^ (**TPA‐0**), 3.8 mol^−1^ (**TPA‐1**), and 3.7 mol^−1^ (**TPA‐2**), respectively (see Table 1). By using equal amounts of Pd‐catalyst in all processes steps **P12**‐**P15** (**case B**) reduced the impact of the catalyst by 63% (**TPA‐0**), 30% (**TPA‐1**), and 20% (**TPA‐2**). By further introducing a Cu‐catalyst (**case C**), despite the lower yield of the Ullmann reaction (87% compared to 95% in BHA), this replacement strongly reduced the environmental impact for **TPA‐0** from V_total,mol_ = 2.4 mol^−1^ (original Pd‐catalyzed route) to V_total,mol_ = 0.42 (new Cu‐catalyzed route), corresponding to a 83% reduction. Similarly, for **TPA‐1** (**P13** and **P14**) and **TPA‐2** (**P12**) this step resulted in a reduction of the overall environmental impact as well. However, since more process steps are required for the synthesis of **TPA‐1** and **TPA‐2** (11 instead of 4 upstream process steps for **TPA‐0**), the lower yield expected with the Cu‐catalyzed route increases the influence of all preceding synthesis steps **P1** to **P11**, which is reflected in the rise in V_total,mol_‐V_cat_. (yellow bars in Figure 6a). Overall, the environmental impact could be reduced substantially by the two in silico steps.

**FIGURE 6:**
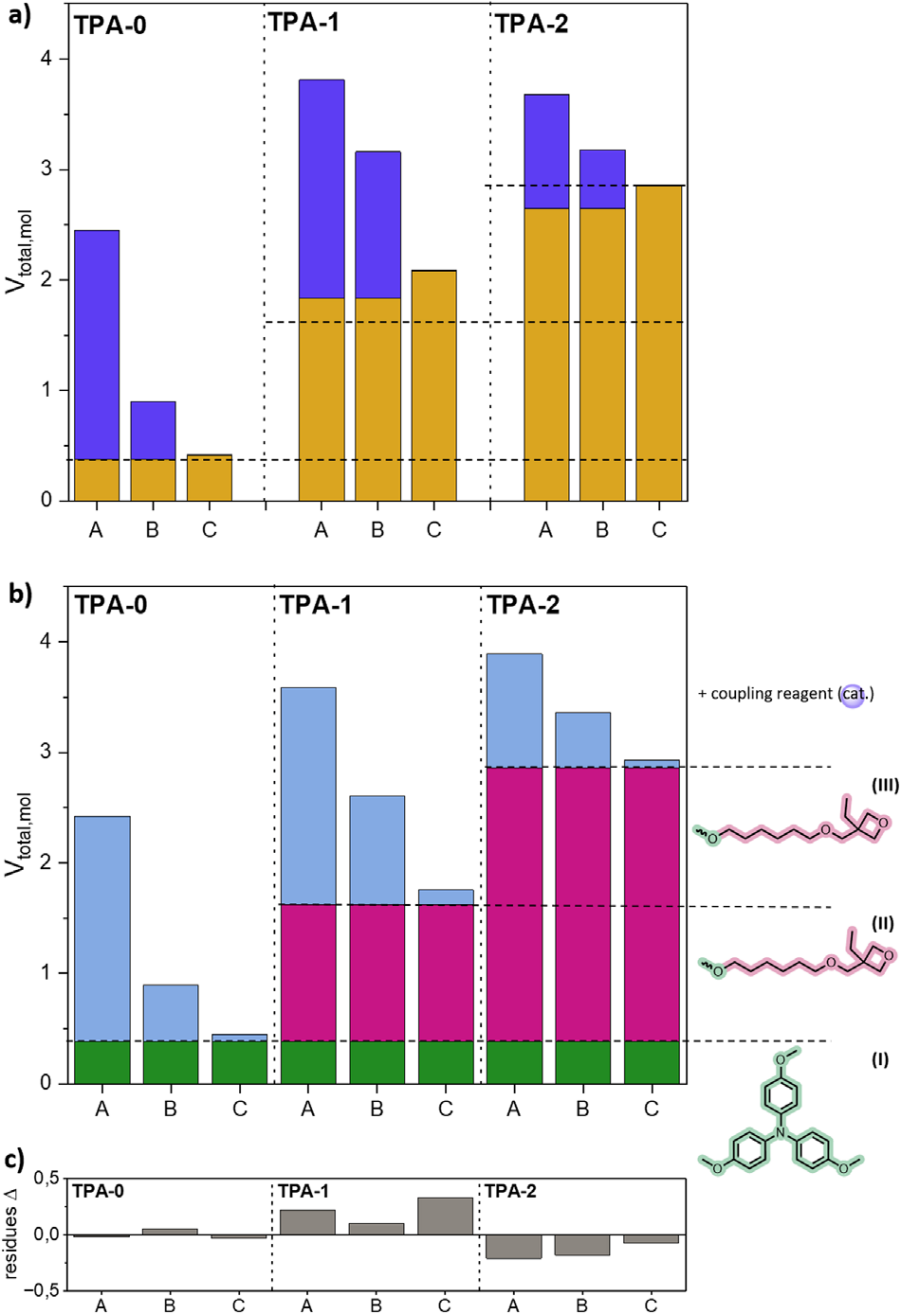
Comparison of TPA syntheses (a) In silico improvements in C─N cross‐coupling reactions. The impact value of the metal catalyst (Pd or Cu) is shown in blue (very small for Cu in case C), all other substances, energy, and waste contributions are summarized in the yellow bars (V_total,mol_‐V_cat._); (b) building block approach assuming V_BBA, core_ = 0.38 (green), V_BBA, arm_ = 1.24 (pink), V_BBA,Pd_ = 1.03, and V_BBA,Cu_ = 0.02 (both light blue); see Figure S7 for details; (c) residues Δ (ESR–BBA). For each material (**TPA‐0** left; **TPA‐1** middle; **TPA‐2** right), the left bar represents the original synthetic route (**case A**) using different amounts of a Pd‐catalyst (Tables S4o‐q), the middle bar reflects the first theoretical improvement (**case B**) using consistently Pd‐values to 0.5 mol%, and the right bar shows formal replacement of the BHA by 3.5 mol% Cu‐catalyzed Ullmann reaction (**case C**). The horizontal dashed lines indicate approximate contributions from (I) the core, (II) contribution by one arm, and (III) by two arms.

In preliminary conclusion, after in silico optimization of the cross‐coupling reaction steps (**P12**‐**P15**) the metal catalyst's contribution to V_total,mol_ became negligeable, leaving only contributions from the redoxactive core and the polymerizable “arms” (in **TPA‐1** and **TPA‐2**). The introduction of the arm(s) results in a factor 5‐7 higher environmental impacts compared to the standalone redox system **TPA‐0**. As expected, the introduction of two polymerizable side chains has a larger impact than only one. The necessity of two metal‐catalyzed steps to build the redox system in **TPA‐1** reduces the ratio V_total,mol_(**TPA‐2**)/V_total,mol_ (**TPA‐1**) < 2.

These qualitative arguments led us to assume three seemingly “independent” contributions (building blocks): (i) the redox‐active **TPA** core, (ii) the polymerizable “arm”, and (iii) the metal‐containing coupling reagent catalyzing the C─N bond formation. This simplified **“building‐block approach (BBA)”** yielded self‐consistent results for all **TPA** syntheses including nine variants and four variables (see Chapter S6), where the residues between V_total,mol_ obtained via ESR or BBA, respectively, are below 5 percent (Figure 6c), demonstrating the validity of the approach.

The BBA approach can facilitate future investigations on other material classes that are synthesized via coupling reactions, that is combine “building blocks”: by estimating the environmental impact of a material from a growing ESR library of building blocks, a complete, more time‐consuming ESR analysis can be avoided. Note, BBA can only be applied for related compounds featuring similar building blocks.

#### 3.1.3 Comparison of ESR to Other Metrics

To conclude our discussion of the organic cathode material, we compare our original results (**case A**) obtained by the novel ESR formalism, considering 18 MICs, with single‐MICs as well as standard mass‐based metrics of green chemistry (Figures 7 and S8) [92, 93]. This discussion can only be qualitative, since these approaches have different assumptions prohibiting a quantitative view.

**FIGURE 7:**
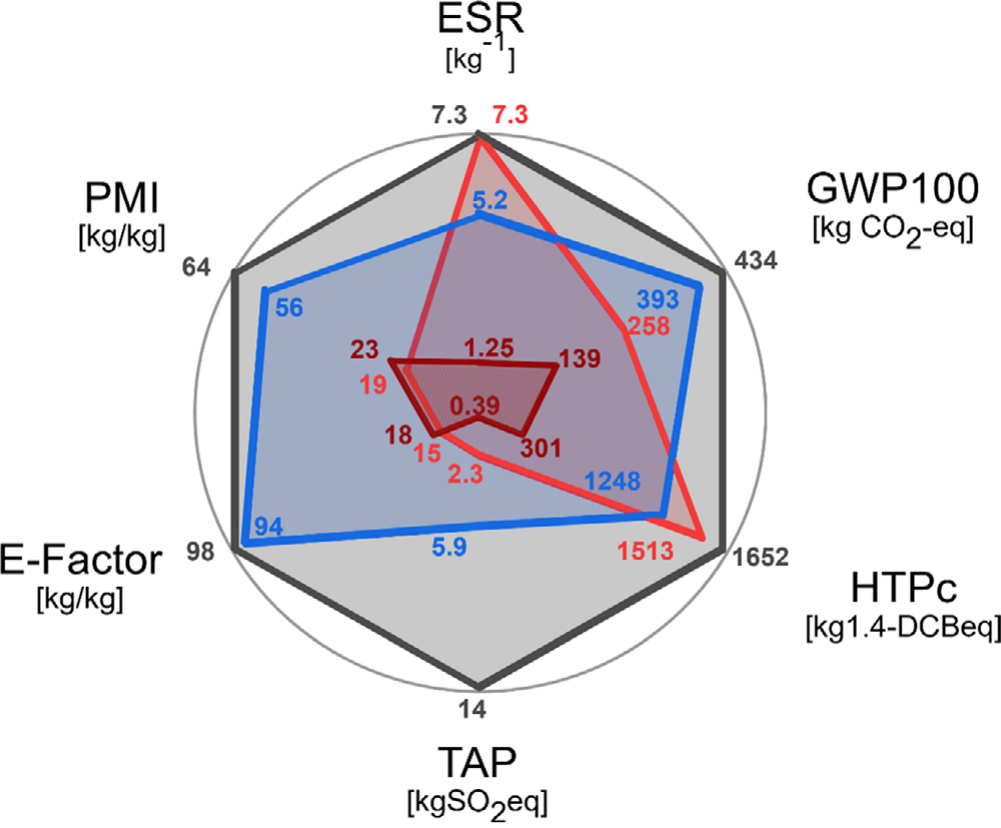
Comparison of ESR to mass‐based metrics: spider plot of the environmental impacts of the synthesis towards **TPA‐0** (red; dark red with Cu‐catalyst), **TPA‐1** (black) and **TPA‐2** (blue) using different approaches: ESR (18 impact categories, with normalization), specific single MICs “HTPc” (MIC 9), “GWP100” (MIC 2) and “TAP” (MIC 1) (all without normalization to the per capita annual environmental output), and the mass‐metrics “E‐Factor” and “PMI”. All values are normalized to the highest value in the respective category, and the actual numbers are given.

Using our novel ESR we obtain a proportional ranking of the environmental impact as **TPA‐0** = **TPA‐1**> **TPA‐2** (where larger means environmentally more harmful). As evident from Figure 3, the ranking of the four syntheses depends on the MIC. When considering only one single MIC such as “human toxicity—cancer (HTPc, MIC 9)”, which was identified as most critical impact category in our study, the relative situation does not change much (**TPA‐0 ≈ TPA‐1**> **TPA‐2,** Figures 4 and 7), which is expected since HTPc is responsible for ca. 80% of the total environmental burden. But when considering “GWP100” (MIC 2), as it is often done in the literature [30, 94, 95, 96], the ranking of the compounds changes slightly (**TPA‐1**> **TPA‐0** ≈ **TPA‐2**). The absolute values are in the range 250–400 kgCO_2_^−^eq/kg, which compares to 369 kgCO_2_^−^eq/kg determined by Carreira et al. for the antiviral drug *Letermovir*, placing both classes of chemical in a similar regime.

For other single MICs such as “TAP” (MIC 1) or “FEP” (MIC 7) one would yield a significantly different picture (**TPA‐1**> **TPA‐0** ≈ **TPA‐2**). In all LCA‐metrics replacing the Pd‐catalyzed BHA reaction by a Cu‐catalyst Ullmann reaction the impact was improved (TPA‐0> TPA‐0*). Finally, the mass‐based metrics E‐Factor (mass of waste/mass of product) [13] and process mass intensity (PMI; total mass in a process/mass of product) [14, 15], yielded **TPA‐1** ≈ **TPA‐2> TPA‐0**), while **TPA‐0** performed best due to the reduced the number of synthetic steps (leading to reduced waste for E‐factor and reduced amount of harmful reagents for PMI). We point out, that the E‐factor values obtained for the **TPA** synthesis are in a similar range as the production of pharmaceuticals in the chemical industry (E‐Factor 25 – 100) [13].

Due to the fact that the environmental “burden” of the individual compounds involved in the syntheses is consistently disregarded, the use of a Cu‐catalyst (TPA‐0*) even slightly increased the mass metrics values compared to **TPA‐0**, due to the reduced yields in the synthesis (TPA‐0* > TPA‐0). This clearly shows, that mass‐based methods do not allow to identify particularly environmentally harmful substances or most critical impact categories (MIC).

Overall, in order not to overemphasize or prioritize a certain view by considering all 18 MICs defined by ReCiPe, not just one, ESR can provide a more holistic view to the environmental impact. Thus, the metric V_total,wt_ takes into account the collective impact of all MICs. By contrast, if only one MIC is considered, other similarly harmful MICs impacting on other damage pathways could be overlooked.

### 3.2 Test Case 2: Inorganic Cathode Material NMC

Next, we applied the novel ESR formalism to the laboratory synthesis as well as the industrial production of the inorganic cathode material **NMCxyz**. Over the last years, the cobalt content in cathode materials has been successively lowered (“nickel‐rich” NMC), driven by economic, environmental, and social considerations [97]. Therefore, three NMCs with different compositions were investigated: **NMC111**, **NMC511**, and **NMC811**. The analysis was based on experimental data for the laboratory processes [98] (see Chapter S8 for further details).

Our results substantiate the environmental reasons: a lower cobalt content decreases the environmental burden (see Table S16): Overall, the synthesis of **NMC811** (V_total,wt_ = 1.0 kg^−1^) on the laboratory scale is 23% less harmful than for **NMC111** (V_total,wt_ = 1.3 kg^−1^). The environmental fingerprint **V_EF_(MIC)** revealed that “Human Toxicity—Cancer” (HTPc; MIC 9) is the most critical MIC in the synthesis or production of **NMCxyz**, see Figure S11, similar to the TPA‐based materials. Further, the process radar **V_PR_(P)** indicated, that the strongest impact is on the process step **Pi** (mixing and milling of precursor oxides), due to the direct impact of the cobalt content. By contrast, **Pii** (thermal conversion) was unchanged, being identical in all cases.

Therefore, the largest (if not only) handle to improve the environmental burden is the improvement of the sintering conditions in **Pii**, which is exactly, what is done for industrial production: the commonly oversized laboratory oven is replaced by an oven with optimized geometry and minimized empty volume. For the state‐of‐the‐art material **NMC811** the industrial production scale was investigated using industrial datasets for the large‐scale production [99] (see Chapter S8 for further details). The comparison of laboratory synthesis and industrial production shows that the first process step (**Pi** and **PI**, respectively) is largely consistent across both scales (V_PR_(Pi) ≈ V_PR_(PI) ≈ 0.37 kg^−1^). Variations in environmental impacts arise predominantly in the second process step, the calcination, see Figure S12. The use of a more energy‐efficient furnace in industrial production reduces V_PR_(PII) = 0.15 kg^−1%^ to 25% of the laboratory V_PR_(Pii) = 0.66 kg^−1^. Overall, the environmental impacts relative to the laboratory synthesis reduced from V_total,wt_ (lab.) = 1.0 kg^−1^ to V_total,wt_ (ind.) = 0.53 kg^−1^, respectively, for **NMC811**.

### 3.3 Comparison of TPA and NMC

The comparison of the two material classes should be done on a laboratory scale focusing on those materials, which are actually useful as battery cathode material. Therefore, **TPA‐0** is disregarded due to its solubility issues. Overall, V_total,wt_ for the synthesis of 1 kg of **NMC811** is about six‐times more environmentally friendly than for **TPA** (V_total,wt_ ≈ 1.0 kg^−1^; 7.3 kg^−1^, and 5.2 kg^−1^, respectively; Table 1). This is rationalized by the fact that while the **TPA** synthesis requires up to maximum 13 steps involving multiple solvents, reactants, and low overall yields, whereas **NMC811** synthesis proceeds in only two steps, using water as the only solvent and achieving nearly quantitative yields. When comparing the molar environmental impact of the material of interest on a laboratory scale, the difference between **NMC** (V_total,wt_ ≈ 0.1 mol^−1^) and **TPA** (V_total,wt_ ≈ 2.45 mol^−1^) is more pronounced (factor ≈ 25, Table 1). This is due to the higher molecular weight of **TPA** compared to **NMC**. However, it can be expected that some environmental credits can be earned for the organic synthesis by upscaling to an industrial scale [100, 101, 102, 103, 104].

The above statements strictly concern the synthesis of 1 kg or 1 mol each of the two material classes, that is the general validity of the ESR formalism. Whenever application‐specific aspects or experimental performance parameters are to be included, V_total_ can be scaled accordingly. For example, in the current case of battery materials, the energy density of 1 kg of active material allows easy calculation of the corresponding V_total_ in units of [kWh^−1^], showing that lab‐scale synthesis of **TPA‐1/‐2** is a factor ca. 40 more environmentally harmful than **NMC811** (Table 1).

### 3.4 Sensitivity Analysis

Sensitivity and uncertainty analyses are suitable for assessing the influence of variable experimental parameters, such as material and energy inputs. In contrast, broader methodological choices, such as the definition of cut‐off criteria or the selection of the database, define the overall framework of the study and were therefore kept constant to ensure consistency across all assessed systems. The assumptions are reported and justified in Chapter S1. Consequently, differences in the calculated ESR values are interpreted as arising from the investigated process alternatives rather than from variations in the underlying modelling approach.

As we already stated above, the largest impact of the **TPA** synthesis routes originates from the use of harmful reagents (∼65%–80%), followed by solvents (∼10%–20%), while the energy consumption and waste disposal play a minor role only (both below 10%; Figure S5). For **NMC**, the reagents and the energy consumption are similarly harmful (Figure S13), while solvents and waste play a minor role.

In order to assess the sensitivity, we have calculated the impact on V_total,wt_ of variations of solvent use (which is often used in large excess in laboratory research) as well as energy consumption (not optimized in laboratory work) by ± 30%. This revealed that the sensitivity varies in the sequence **TPA‐0/TPA‐1/TPA‐2** from ca. 1% to 6% for solvents and from 0.1% to 1.3% for energy (see Figure S6). Thus, V_total_ is sensitive to changes in the amount of solvent used than to changes in the energy demand. This is also reflected in the generally higher contribution of the solvent to the overall environmental impacts compared with the contribution of energy use, in agreement with the pie charts shown in Figure S5. For **NMC**, V_total,wt_ varies by 13%–16% when changing the energy consumption. For industrial scale production of **NMC811** the variation is less (5.5%) due to improved oven energy efficiency. As expected, the synthesis is mostly insensitive to solvents (< 1%; Figure S14).

In conclusion, the sensitivity of our ESR analysis is small for all **TPA** as well as for most **NMC** synthesis (few percent change for 30% change in input parameter). Importantly, this demonstrates that variations do not change the absolute values much, and the ranking of the compounds we studied is not affected.

## 4 Conclusion

One aim of this work is to provide academic researchers with a simple, ready‐to‐use tool to evaluate the environmental impact of the synthesis of new materials on the laboratory scale. Additionally, the tool should be broadly applicable to different research fields independently of the technological status. For that, we introduced the **Environmental Synthesis Radar (ESR)** formalism as a methodological framework designed to systematically assess the environmental impacts of chemical synthesis routes. Unlike existing studies which only focus on selected midpoint impact categories (MICs) such as “global warming” or “particulate matter formation”, **ESR considers the complete set of 18 MICs** (as defined by ReCiPe), thus, ensuring a **more holistic and conclusive evaluation**. Normalization of the individual impacts to the annual environmental emissions per capita, that is, the worldwide annual environmental emissions divided by the world's population, enables comparability of results across different MICs and facilitates contextualized interpretation.

ESR yields three environmental metrics: the metric **V_total_** offers a comprehensive measure of environmental burden across all midpoint impact categories (MICs) and synthesis steps, contextualized by referencing to the annual environmental emissions per capita. Since it is a single number, it facilitates straightforward comparisons between different material classes. Additional, more specific indicators are the environmental fingerprint **V_EF_(MIC)** and the process radar **V_PR_(P)**. The former reveals the most critical MICs of a given synthesis, while the latter identifies the most environmentally harmful synthesis steps (caused by high environmental burden of the substances involved or excessive energy consumption) and also offers a basis for targeted improvements, for example by in silico optimization. Please note, that experimental performance parameters are not considered in our generic analysis, since these would be specific for a given material or application. Thus, ESR should not be regarded as a standalone measure of the ecofriendliness of a material, but rather as a framework for identifying environmentally favorable synthesis strategies. Otherwise, this may lead to “false‐green” conclusions.

As test cases for demonstrating and illustrating the **ESR** methodology and its evaluation strategy, we selected two classes of cathode materials used in secondary batteries: an organic system based on **TPA** and an inorganic reference based on **NMC**. In the TPA synthesis, **V_PR_(P)** identified LAH, bromine, and the Pd included in the catalyst for C─N coupling as most harmful. All three results were expected [30, 42], and this clearly demonstrates the validity of our approach. Using in silico improvements, we replaced the commonly utilized, but environmentally most harmful Pd‐catalyzed coupling reaction by the Cu‐catalyzed analog. This led to a significant improvement of V_total,mol_. Such insights cannot be obtained by mass‐metrics‐based approaches, where all compounds are treated environmentally nondescript.

Since organic cathode materials require functionalization of the bare redox system with polymerizable groups to render them insoluble, it was expected that this would increase the environmental impact due to the increased number of synthetic steps. By considering molar quantities, ESR was able to quantify this impact. In our case, functionalization led to a ca. 40% increase going from **TPA‐0** (nonpolymerizable reference) to **TPA‐1/‐2** (polymerizable/cross‐linkable), which can be considered quite large. The impact of functionalization is even larger after in silico improvement, replacing the Pd‐catalyzed Hartwig‐Buchwald amination by Cu‐catalyzed Ullmann coupling (factor 5‐7). This is due to the harmful chemicals and the many (seven) steps involved in the synthesis of the crosslinkable oxetane arm(s).

This work is the first example detailing the impact of functionalization by polymerizable groups on the environmental impact. Note, that the absolute values will vary for different polymerization chemistries, that is, when different spacers and reactive groups other than C6‐alkyl and oxetane will be used such as derivatives of styrene, (methyl‐) methacrylate, etc.

Furthermore, we introduced the building block approach (BBA), showing strong potential for future application of the ESR formalism for coupling reactions in general, for example, addition and condensation reactions used to assemble (conjugated) molecules or polymers. After establishing a library of building blocks, the environmental impact of future products can be quickly predicted and with sufficient accuracy, allowing for informed adjustments of select building blocks and reaction chemistry with low error margins. A detailed ESR treatment is not required any more.

Applying the ESR formalism to the state‐of‐the‐art **NMC811** cathode material confirms the generally accepted expectation that increasing the nickel content at the expense of the cobalt content will significantly lower the environmental impact. Going from **NMC111** to **NMC811** reduced the environmental burden by 23%. **V_PR_(P)** identified the calcination as most harmful step. Scaling from laboratory synthesis to industrial production halves the overall environmental burden of **NMC811**, primarily due to improved energy efficiency during calcination (process step **PII**).

Finally answering our central question ***How “Green” is your Functional Material?*** our results demonstrate that the syntheses of 1 kg of organic **TPA** as well as inorganic **NMC** have an environmental impact not far below the annual environmental emissions per capita (about 1/3 for TPA, 1/18 for NMC), indicating that both are environmentally quite harmful. For both material classes, **V_EF_(MIC)** revealed “Human Toxicity—Cancer” (HTPc; MIC 9) as most impactful MIC. We attribute that to the involvement of environmentally critical chemicals in both cases.

In comparison, the organic material class **TPA** performs environmentally inferior (average factor 6) compared to the lab‐scale **NMC811**, mostly due to the involvement of harmful chemicals in the polymerizable groups, limited yields, and the use of excess solvents in organic synthesis, while **NMC** production is water‐based and reaches quasi‐ideal yields. By in silico optimization of the **TPA‐1/‐2** synthesis we could reduce V_total,mol_ by a factor of 4, closing down the gap to lab‐scale **NMC** to similar levels. We expect that advances in synthesis methods and scalable production will improve the production of organic pi‐conjugated materials in the coming years. Since all synthesis routes (organic and inorganic) were assessed using identical assumptions, databases and system boundaries, a consistent comparison was ensured.

Having in mind the global demand for energy and energy storage, as a final validation of our approach and its practical applicability, we apply the results obtained using the ESR analysis of industrially produced **NMC811** to estimate the environmental impact of global lithium‐ion battery (LIB) production. Using 2024 as the reference year [105, 106], we find that the production of LIB active materials accounts for approximately 1% of total global emissions (see Chapter S9 for details), in agreement with current market assessments. It should be noted that this estimate represents a lower bound, as it assumes (i) the theoretical capacity of **NMC811** (see Table 1), which is rarely achieved in practice (ca. 175‐190 mAh/g (77) vs. 275 mAh/g, that is, 80% Table 1), and (ii) considers only the production of the active material, excluding additional materials and processing steps required for complete battery cell manufacturing (ca. 50% [106]). Nonetheless, this clearly demonstrates the validity of the ESR‐formalism.

In overall conclusion, the ESR formalism enables quantification of the “greenness” of a material synthesis relative to the annual emissions of one human being (per capita emissions). One of its main advantages is its wide applicability to different use cases, that is, types of material (such as organic or inorganic, liquid or solid), or stages of technological development (industrial production vs. lab scale synthesis), future work may extend the ESR to other emerging technologies, for example battery recycling. In the future, due to the global energy demand which is predicted to steeply increase, to maintain operational or financial leverage, one should implement risk management tools (such as stop‐losses) and strict position sizing at the earliest stages. Assessments such as the ESR formalism we introduced here will become more important and should be implemented at an early stage to keep the leverage. Therefore, our work represents an important step towards integrating LCA into (early‐stage) research. Based on our results, researchers will be able to complement their technical advances (higher efficiency, life time, etc.) with assessment and potential improvements of the environmental performance of their material at a point when their designs are still highly flexible.

## Author Contributions

**Laura Plein**: methodology, data curation, validation, visualization, writing – review and editing. **Alexander Schommer**: methodology, data curation, validation, visualization, writing – review and editing, software. **Florian Dietrich**: methodology, data curation, writing – review and editing. **Moritz Gutsch**: conceptualization, methodology, writing – review and editing, validation. **Kevin Schuller**: methodology, validation, data curation, writing – review and editing. **Heike Klesper**: validation, writing – review and editing, formal analysis. **Lars Jahnke**: conceptualization, methodology, writing – review and editing, software, validation, formal analysis. **Jens Leker**: conceptualization, resources, supervision, methodology, writing – review and editing, formal analysis, project administration. **Klaus Meerholz**: conceptualization, methodology, validation, funding acquisition, writing – original draft, writing – review and editing, project administration, formal analysis, supervision, data curation, resources, visualization.

## Conflicts of Interest

The authors declare no conflicts of interest.

## Supporting information




**Supporting File**: adma74993‐sup‐0001‐SuppMat.pdf.

## Data Availability

The data that support the findings of this study are available on request from the corresponding author. The data are not publicly available due to privacy or ethical restrictions.
