# Supplementary material for: How “Green” is Your Functional Material? Assessing Environmental Impacts of Multi‐Step Synthetic Protocols in Material Sciences at Various Technology Levels
Source: Adv Mater. 2026 Sep 24;38(62):e74993. doi: 10.1002/adma.74993 (PMC13741109; doi:10.1002/adma.74993)
Supplement: Supplementary file 1 [file ADMA-38-e74993-s001.pdf]

# SUPPORTING INFORMATION

# How “Green” is your Functional Material? Assessing Environmental Impacts of Multi-Step Synthetic Protocols in Material Sciences at Various Technology Levels

Laura Plein[1§], Alexander Schommer[2§], Florian Dietrich[1,2], Moritz Gutsch[2], Heike Klesper[1], Kevin Schuller[1], Lars Jahnke[2], Jens Leker[2,3], Klaus Meerholz[1*]

1 Department of Chemistry and Biochemistry, University of Cologne, Greinstrasse 4-6, 50939 Cologne, Germany

2 Institute of Business Chemistry, University of Münster, Leonardo-Campus 1, 48149 Münster, Germany

3 Helmholtz Institute Münster, IEK-12, Forschungszentrum Jülich GmbH, 48149 Münster, Germany

## TABLE OF CONTENTS

**CHAPTER S1) METHODOLOGY OF LIFE CYCLE ASSESSMENT ......................... 3**

Chapter S1a) Midpoint Impact Categories (MICs) ........................................................................................ 3

Chapter S1b) General assumptions life-cycle inventory ................................................................................ 4

**CHAPTER S2) TPA LAB SYNTHESIS ..................................................................... 6**

Chapter S2a) **TPA** Steps performed in our laboratory (unpublished) ........................................................ 6

Chapter S2b) **TPA** Overview process details.................................................................................................. 11

Chapter S2c) **TPA** Life Cycle Inventory ........................................................................................................... 13

**CHAPTER S3) ORIGINAL DATA FOR THE SYNTHESIS OF TPA AND NMCXYZ 30**

**CHAPTER S4) NORMALIZATION FACTORS ......................................................... 32**

**CHAPTER S5) ENVIRONMENTAL MATRIX EM FOR TPA .................................... 33**

Chapter S5a) Total impacts of the individual process steps of **TPAs** and the entire synthesis ............. 36

Chapter S5b) Contribution of Reagents, Energy, Solvents and Waste for **TPA** Synthesis .................... 37

**CHAPTER S6) BUILDING BLOCK APPROACH (BBA)...................................................... 39**

**CHAPTER S7) COMPARISON TO OTHER METRICS........................................................ 40**

**CHAPTER S8) NMC LAB-SCALE SYNTHESIS & INDUSTRIAL PRODUCTION... 41**

Chapter S8a) **NMC** synthesis ............................................................................................................................ 41

Chapter S8b) Overview process details for all four **NMC** synthesis .......................................................... 43

Chapter S8c) **NMC** Life Cycle Inventory ........................................................................................................... 44

Contributions of each Process Step for **NMC811** lab-synthesis ................................................................. 46

Chapter S8d) Environmental Matrix **EM** (process steps vs. MICs) for **NMC**............................................ 48

Chapter S8e) Total impacts of the individual process steps of **NMCxyz** and the entire synthesis........ 49

Chapter S8f) Contribution of Reagents, Energy, Solvents and Waste for **NMC** Synthesis .................... 52

**CHAPTER S9) ESTIMATION OF WORLD-WIDE LITHIUM-ION BATTERY (LIB) PRODUCTION - VALIDITY AND APPLICABILITY OF THE ESR FORMALISM ........................... 54**

**CHAPTER S10) REFERENCES IN SUPPORTING INFORMATION ....................... 55**

# Chapter S1) Methodology of Life Cycle Assessment

## Chapter S1a) Midpoint Impact Categories (MICs)

We used the life cycle impact assessment (LCIA) characterization model **ReCiPe 2016 v1.03, midpoint (E)**[1] (simply "ReCiPe" from now on) that converts life cycle inventory data - such as emissions and resource extractions - into environmental impact scores using scientifically derived characterization factors. At the midpoint level, it quantifies impacts in 18 categories by applying substance-specific parameters that reflect the relative contribution of each emitted or extracted substance to a given environmental mechanism, based on fate, exposure, and effect modeling. These midpoint results represent specific problem-oriented indicators, forming the scientific basis for optional aggregation into endpoint categories related to human health, ecosystem quality, and resource depletion. The 18 midpoint impact categories (MICs) are listed in the following Table S1. Furthermore, ReCiPe defines three endpoint areas of protection, comprising the different MICs (Table S2).

### Table S1: Midpoint Impact Categories

**Table S1**: Midpoint impact categories (MICs) defined by *ReCiPe2016*.

| # | **MIC** | **Abbreviation*** | **Physical Units** |
|---|---|---|---|
| 1 | Acidification potential terrestrial | TAP | [kg $SO_2$-Eq.] |
| 2 | Climate change, global warming potential | GWP100 | [kg $CO_2$-Eq.] |
| 3 | Ecotoxicity – fresh water | FETP | [kg 14-DCB-Eq.] |
| 4 | Ecotoxicity – marine | METP | [kg 14-DCB-Eq.] |
| 5 | Ecotoxicity – terrestrial | TETP | [kg 14-DCB-Eq.] |
| 6 | Energy resources: non renewable fossil | FFP | [kg oil-Eq.] |
| 7 | Eutrophication: freshwater | FEP | [kg P-Eq.] |
| 8 | Eutrophication: marine | MEP | [kg N-Eq.] |
| 9 | Human toxicity – carcinogenic | HTPc | [kg 14-DCB-Eq.] |
| 10 | Human toxicity – non-carcinogenic | HTPnc | [kg 14-DCB-Eq.] |
| 11 | Ionizing radiation | IRP | [kBq Co-60-Eq.] |
| 12 | Land use | LOP | [$m^2$·a crop-Eq.] |
| 13 | Material resources: metals, minerals | SOP | [kg Cu-Eq.] |
| 14 | Ozone depletion | ODP infinite | [kg CFC-11-Eq.] |
| 15 | Particulate matter formation | PMFP | [kg PM2.5-Eq.] |
| 16 | Photochemical oxidant formation - human health | HOFP | [kg $NO_x$-Eq.] |
| 17 | Photochemical oxidant formation - terrestrial ecosystems | EOFP | [kg $NO_x$-Eq.] |
| 18 | Water use | WCP | [$m^3$] |

* Abbreviations following *ReCiPe*.

### Table S2: Endpoint Impact Categories

**Table S2**: Endpoint areas of protection defined by *ReCiPe2016.*

| **Endpoint areas of protection** | **Issues covered (damage pathways)** |
|---|---|
| Damage to human health | increase in respiratory disease, various types of cancer, other diseases and malnutrition |
| Damage to ecosystems | damage to freshwater species, terrestrial species, and marine species |
| Damage to resource availability | increased extraction costs |

## Chapter S1b) General assumptions life-cycle inventory

To compromise between data availability, reasonable time required to calculate the impacts and accuracy of results, LCAs commonly make use of assumptions. We have listed our assumptions in the following.

## Substance inputs

1. Precursors are selected based on lowest cost and availability.
2. Substances, which are involved in a given process step with less than 10%wt (cutoff threshold) are listed in the life cycle inventory, but are not considered in the calculations. Importantly, this cutoff criterion is not applied to metals and metal-containing substances due to their strong environmental impact. For metal-organic complexes (e.g. transition metal catalysts) only the metal is considered and the ligands are neglected).
3. Whenever a certain chemical can easily be "divided", Wernet et al.[2] proposed to replace it in the calculation by similar substances with the same sum formula:
   - replace copper iodide (CuI) by copper and iodine,
   - replace lithium aluminum hydride ($LiAlH_3$) by lithium, aluminum and hydrogen,
   - replace sodium *tert*-butoxide ($Na(O^tBu)$) by sodium and butanol,
   - replace oxalyl chloride ($C_2O_2Cl_2$) by oxalyl acid and chlorine.

   This procedure keeps the number of process steps to be evaluated to a minimum.
4. Whenever the amount of solvent used in a certain process step is not given in the literature, we assumed it to be 20 times the molar amount of the main (major carbon contributing) educt.
5. For each substance, a "market" (supplier) was chosen in EcoInvent 3.9.1. This market does not represent one specific source or production technology, but constitutes a weighted average of different production methods used within a specific region. Whenever possible, we chose "Germany", otherwise "Europe", or "Global". Details are given in the "Dataset" column in the life cycle inventory.
6. Finally, we did not consider the reaction atmosphere (ambient, inert gas, pressure) nor the pre-cleaning and drying of the reaction vessels before the respective process step.
7. The Workup of the materials/target molecules was not considered, since downstream purification steps are highly process-specific, lack reliable and comparable data, and fall outside the study's focus on synthesis efficiency and core reaction steps.

## Energy Input

The energy consumption (heating or cooling) of a process or laboratory scale synthesis is calculated following the physicochemical methodology proposed by Piccinno et al..[3] However, in our study, we have replaced the insulation with the minimum convective heat transfer coefficient (h) of air, based on the value provided by Kosky et al..[4] Equation S1 takes into account the energy required for the initial heating of the reaction mixture to the desired temperature and the energy required to maintain the reaction temperature over the period of the reaction time. The respective quantities of the substances and the solvent, as well as their specific heat capacity of the solvent, were incorporated into the calculation.

The surface area of the reaction vessel was calculated for each individual process step based on the quantities used and the different densities of the substances and the solvent for a round flask. If no specific density was known, a density of 1 $g/cm^3$ was assumed as an approximation. Following Piccinno et al., a correction factor of 1.1 (1.35) was assumed as the industrial standard during heating (cooling).[3] Heat recovery was not considered.

The German electricity mix from 2014-2022 (available in Ecoinvent) is used as the energy source.[5]

We assumed that the cooling water was not contaminated during the processing and could, therefore, be returned untreated to the biosphere after use.

The energy required to establish reduced pressure is negligible compared to the energy required for heating and is, therefore, not considered.

$$Q_{react} = \frac{Q_{heat}+Q_{loss}}{\eta_{heat}} = \frac{[C_p \cdot m_{mix} \cdot (T_r - T_0)] + [A \cdot h \cdot (T_r - T_{out}) \cdot t]}{\eta_{heat}} \cdot F \qquad \textbf{S1}$$

with

| | | |
|---|---|---|
| $Q_x$ | = | heat for process x |
| $\eta_{heat}$ | = | efficiency of heating element, 90% was assumed |
| $C_p$ | = | heating capacity at constant pressure |
| $m_{mix}$ | = | mass of solvent and educt mixture |
| $T_r$ | = | process temperature |
| $T_0$ | = | starting temperature, room temperature |
| A | = | surface area of reaction vessel/flask |
| h | = | convection heat transfer coefficient h, 2.5 W/(m$^2$·K) was assumed |
| $T_{out}$ | = | temperature outside reaction vessel/flask (25C was assumed in laboratory) |
| t | = | reaction time |
| F | = | industrial correction factor (F = 1.1 for heating, F = 1.35 for cooling) |

## Waste output (spent solvent mixture)

In addition to the desired product of each process step, waste is produced, containing the complete solvent (100%), unreacted educts, by-products, and (stoichiometric) co-products. The treatment of the waste varies considerably depending on the specific details of the reaction and where (location, i.e. country or even region) it is performed[6] and it is very costly to determine the exact composition of the by-products for each process step. Therefore, by- or co-products that could potentially be used for other products in an industrial setting (receive credits), are considered to be part of the waste treatment process. for liquid or solid co-products, or are assumed to be emitted to the air if they are gaseous. In other words, the waste is the total weight of the inputs to a given process step minus the product (1 kg). As such, input and output are balanced.

The resulting waste quantities are treated using the generic waste treatment process "spent solvent mixture" that is representative of the incineration of hazardous waste with energy recovery for a mixture of different solvents in Europe, with the exception of Switzerland from the EcoInvent database (see Table S3, last line).

## Figure S1: Schematic illustration of a generic process step P#:

The generic process of 1 kg of a target material is schematically illustrated in Figure S1.

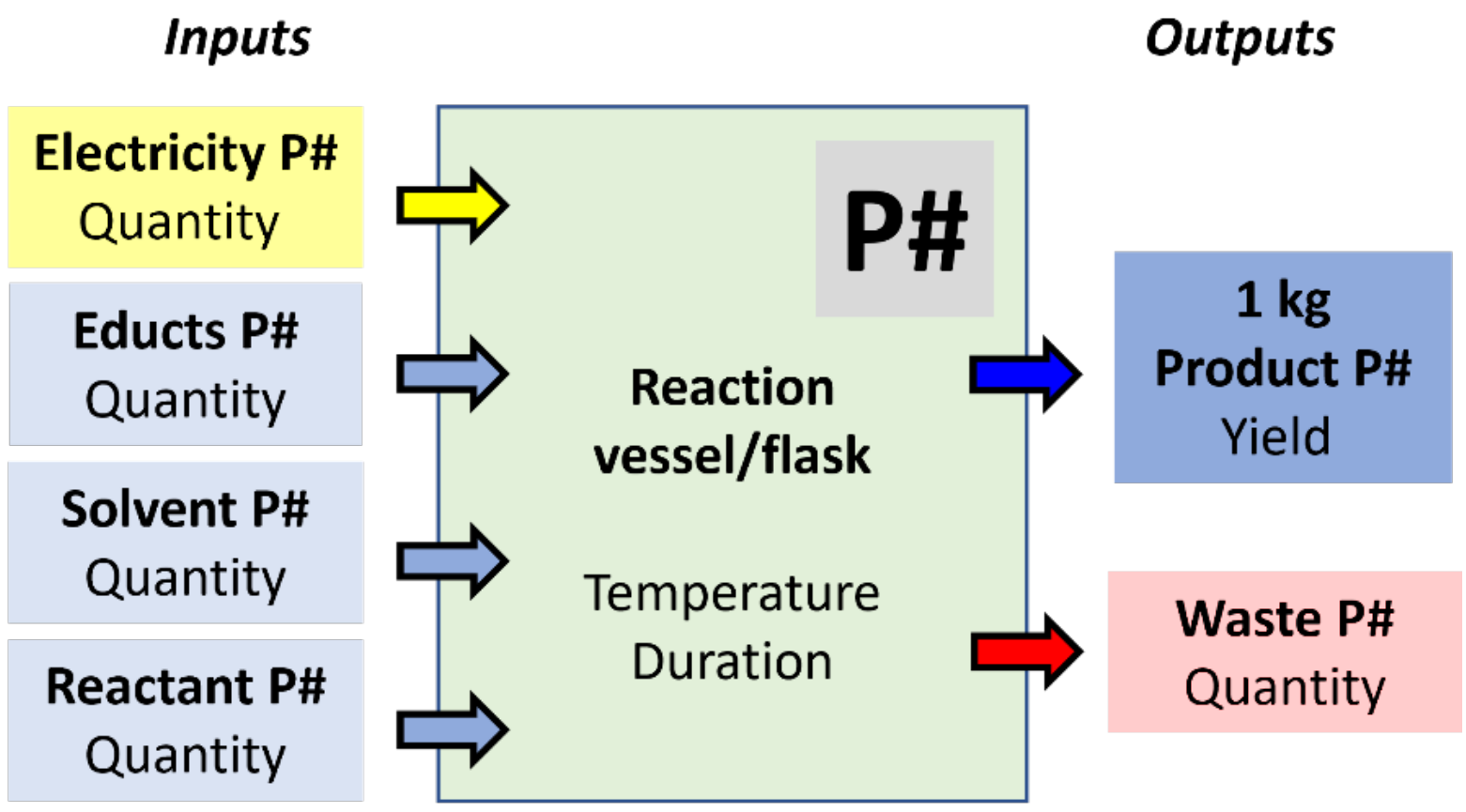


**Figure S1***:* Each process has several inputs (left) and output streams (right) as indicated exemplarily. Several such processes **P#** are included in complete system (Figure 2, main text).

# Chapter S2) **TPA** Lab Synthesis

## Chapter S2a) **TPA** Steps performed in our laboratory (unpublished)

Characterizations (NMR, MS, etc.) are not relevant to the LCA and will be reported separately.

### Process step **P7** towards **M10**

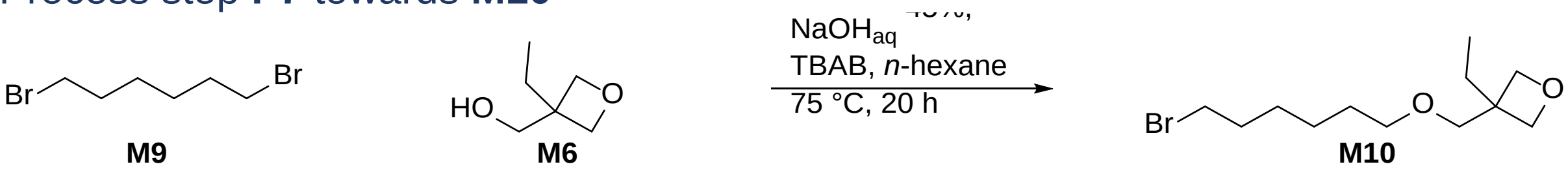


**Scheme S1:** Synthesis step **P7** to obtain **M10**.

According to a procedure from *Jungermann et al.*[7], to a solution of 6.00 mL (6.11 g, 52.6 mmol, 1.00 eq.) 3-ethyl-3-oxetanemethanol (**M6**), 24.0 mL (38.6 g, 158 mmol, 3.01 eq.) 1,6-dibromohexane (**M9**) and 864 mg (2.68 mmol, 0.05 eq.) tetrabutylammonium bromide dissolved in 200 mL *n*-hexane, 35 mL of 45% $NaOH_{aq}$ solution were added (Scheme S1). After the reaction mixture was stirred under reflux at 75 °C for 6 h, 150 mL water were added. The aqueous layer was extracted by *n*-hexane. The combined organic phases were dried over $MgSO_4$ and concentrated. The crude product was purified *via* column chromatography over $SiO_2$ eluting first with *n*-hexane, changing to THF. The desired product (**M10**) was obtained in a yield of 14.5 g (52.1 mmol, 99%) as a yellowish oil.[8]

### Process step **P11** towards **M15**

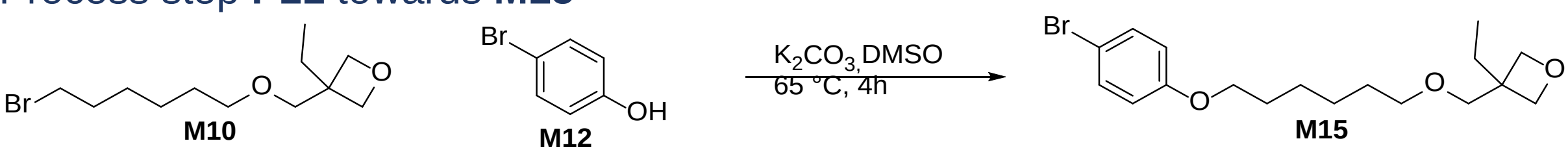


**Scheme S2:** Synthesis step **P11** to obtain **M15**.

3-(6-(4-bromophenyl)oxyhexyloxymethyl)-3-ethyloxetane (**M15**) was synthesized based on a route of Jungermann et al.[7] as shown in Scheme S2. The desired product (**M15**) could be obtained in a yield of 97%.

### Process step **P14** towards **TPA-1**

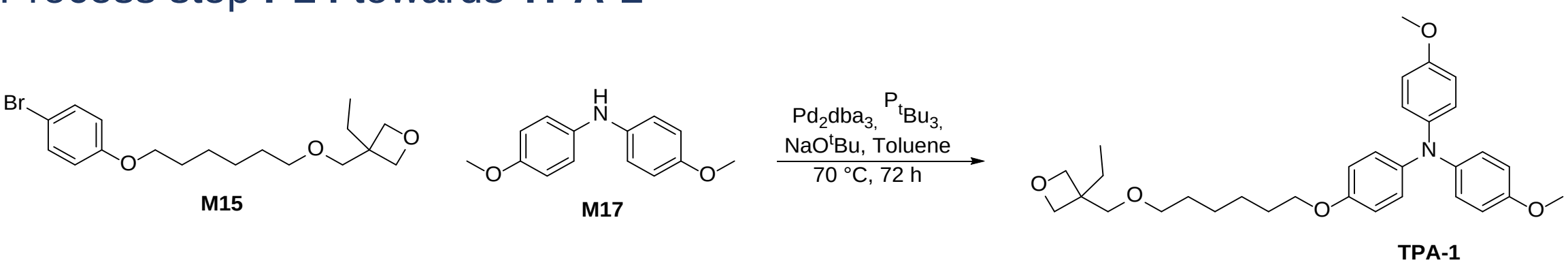


**Scheme S3:** Synthesis step **P14** to obtain **TPA-1**.

Prepared under inert atmosphere, the catalyst mixture consisting of 91.6 mg (0.10 mmol, 0.01 eq.) tris(dibenzylideneacetone)dipalladium ($Pd_2(dba)_3$) and 32.4 mg (0.16 mmol, 0.016 eq.) tri-*tert*-butylphosphine $P(^tBu)_3$ dissolved in 2 mL toluene was allowed to pre-react for 30 minutes (Scheme S3). The catalyst mixture was added dropwise to a prepared mixture of 2.29 g (10 mmol, 1.00 eq.) bis(4-methoxyphenyl) amine (**M17**), 4.08 g (11 mmol, 1.13 eq.) 3-(6-(4-bromphenyloxyhexyloximethyl)-3-ethyloxetane (**M15**) and 1.20 g (12.5 mmol, 1.25 eq.) sodium-*tert*-butoxide in 20 mL toluene. The reaction mixture was stirred under monitoring the conversion via TLC at 70 °C for 72 h. Afterwards, the reaction mixture was diluted with water and extracted with MTBE three times. The combined organic phases were dried over $MgSO_4$ and concentrated. The crude product was purified by column chromatography over $SiO_2$ eluting with cyclohexane/ethyl acetate 9:1. The desired product **TPA-1** was obtained in a yield of (4.16 g, 8.01 mmol, 80%) as a yellow viscous oil.[9]

## Process step **P12** towards **TPA-2**

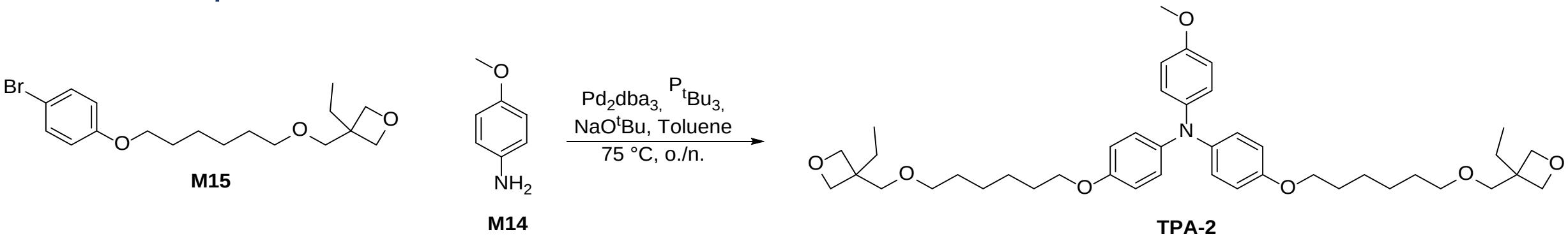


**Scheme S4:** Synthesis step **P12** to obtain **TPA-2**.

Under inert conditions, a precatalyst solution was prepared from 7.4 mg tris(dibenzylideneacetone)dipalladium ($Pd_2(dba)_3$) (0.081 mmol, 0.01 eq.) and 2.6 mg tri-*tert*-butylphosphine (0.130 mmol, 0.016 eq.) dissolved in 15 mL toluene (Scheme S4). After stirring for 30 min, subsequently, 1.00 g (8.12 mmol, 1.00 eq.) *p*-anisidine **M14**, 6.66 g (17.9 mmol, 2.21 eq.) 3-(6-(4-bromophenyl)oxyhexyloxymethyl)-3-ethyloxetane **M15** and 1.95 g (20.3 mmol, 2.5 eq.) sodium-*tert*-butoxide were added with an additional portion of 5 mL toluene. The mixture was stirred at 75°C overnight and the conversion was monitored using thin layer chromatography (TLC). Afterwards, the reaction mixture was diluted with water and washed with MTBE three times. The combined organic phases were dried over $MgSO_4$, filtered, evaporated and purified over silica gel (cyclohexane:ethyl acetate 9:1) to yield **M15** (5.49 g, 7.80 mmol, 96%) as a yellow viscous substance.[10]

## Retro-synthesis steps

The synthesis steps **P1**-**P6**, **P8**-**P10, P13** and **P15** were not performed in our laboratory, but are based on literature reports. These routes were chosen to trace back to the compounds that can be found in the Ecoinvent database (Figure 2, main text). The “theoretical” retro-synthesis steps from literature were selected based on high yields, lowest possible number of reaction steps and lowest possible amount of (transition) metals necessary. Products, yields and data sources are given in Table S3. The corresponding flowcharts can be seen for **TPA-0** in Figure S2, **TPA-1** in Figure S3, and **TPA-2** in Figure S4.

## Figure S2: Flow chart **TPA-0** synthesis

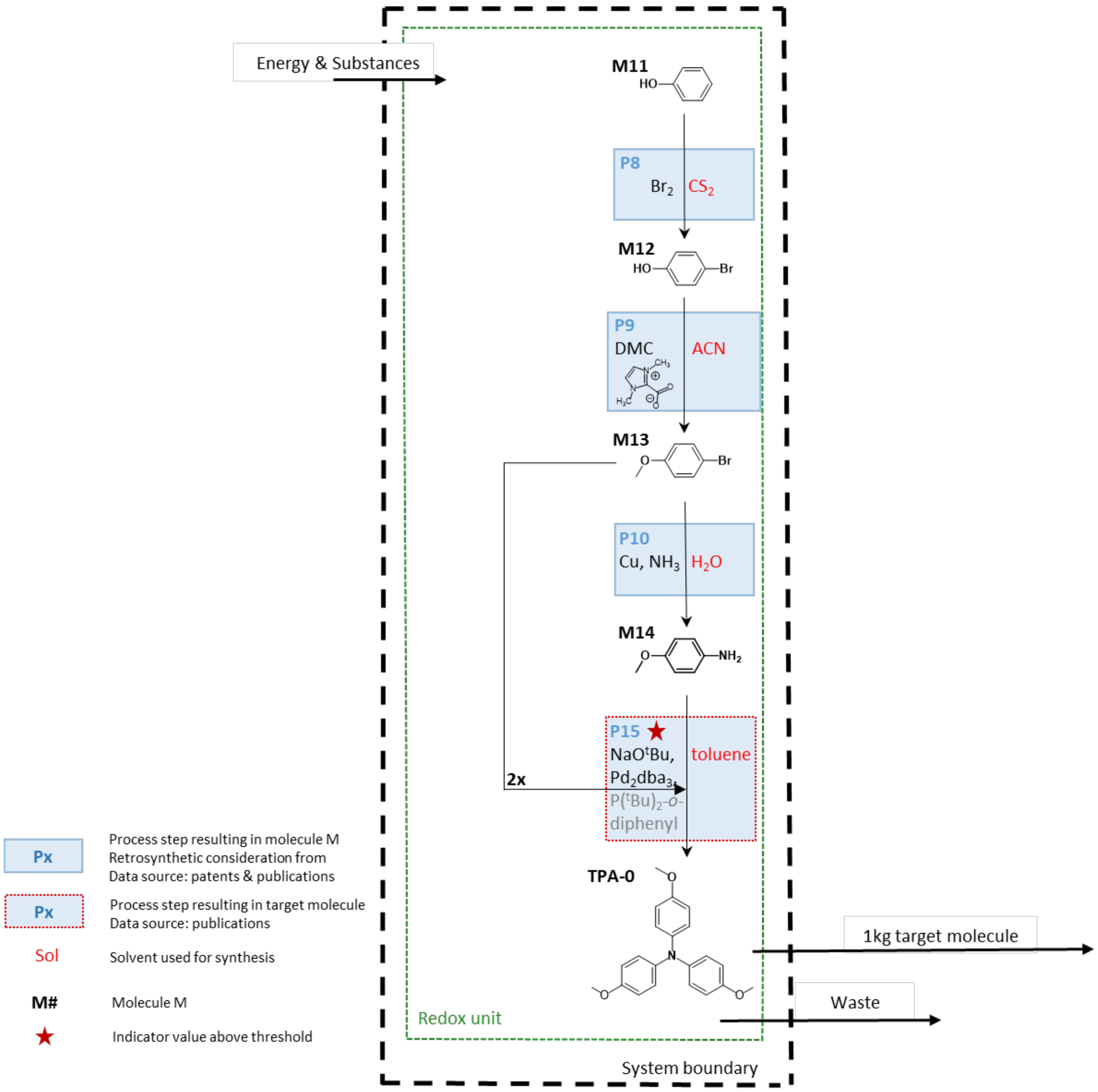


**Figure S2:** Flowchart for the system boundary for the laboratory synthesis of **TPA-0**. Compounds **M13**, and **M14** are commercially available and used in **P15**, in literature laboratory-scale synthesis. Materials in light gray letters are below the LCA cut-off of 10 mol%; therefore, they are not considered in the impact calculations. A detailed list of all solvents and reactants used can be found in Table S3. The red star highlights the environmentally most critical process step as identified by our ESR-method. Abbreviations are: ACN = acetonitrile, dba = dibenzylideneacetone, DMC = dimethyl carbonate, and $^t$Bu = *tert*-butyl.

## Figure S3: Flow chart **TPA-1** synthesis

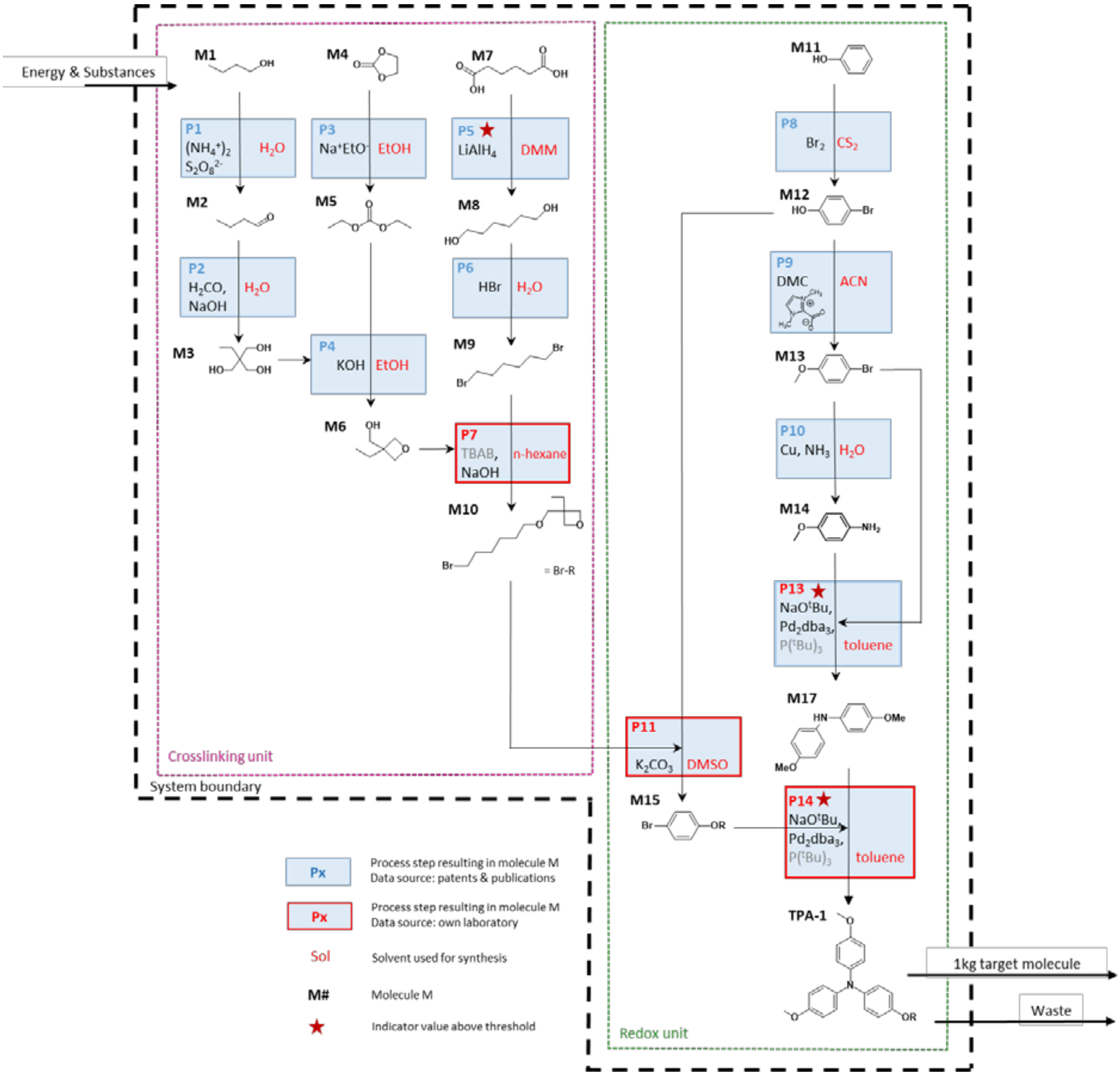


**Figure S3:** Flowchart for the system boundary for the laboratory synthesis of **TPA-1**. Compounds **M6**, **M9**, **M12,** and **M17** are commercially available and used in **P7**, **P11** and **P14** in laboratory-scale synthesis. Materials in light gray letters are below the LCA cut-off of 10 mol%; therefore, they are not considered in the impact calculations. A detailed list of all solvents and reactants used can be found in Table S3. The red stars highlight the environmentally most critical process steps as identified by the ESR-method. Abbreviations are: ACN = acetonitrile, dba = dibenzylideneacetone, DMC = dimethyl carbonate, DMM = dimethoxymethane, DMSO = dimethyl sulfoxide, Me = methyl, Et = ethyl, TBAB = tetrabutylammonium bromide, and $^t$Bu = *tert*-butyl.

## Figure S4: Flow chart **TPA-2** synthesis

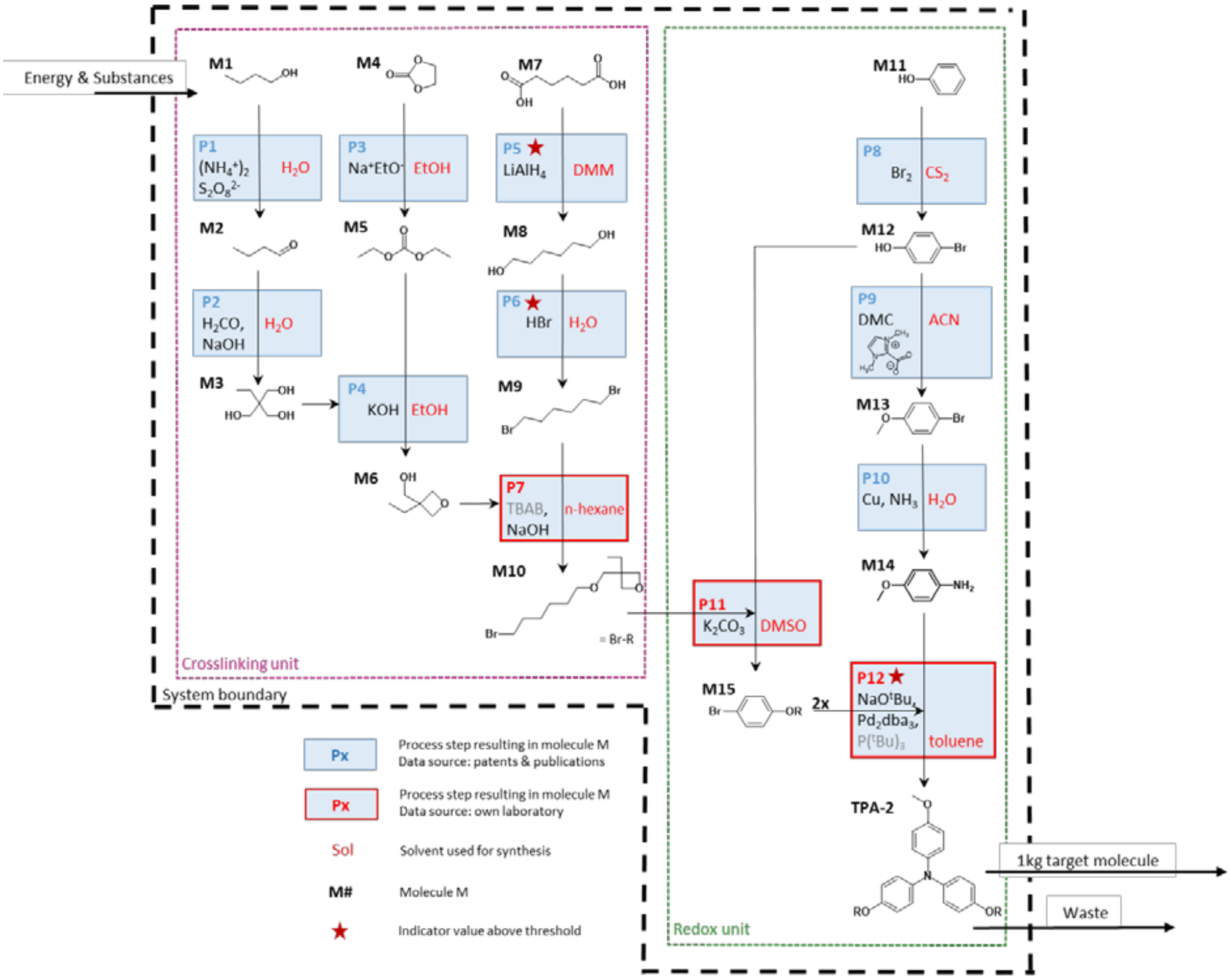


**Figure S4:** Flowchart for the system boundary for the laboratory synthesis of **TPA-2**. Compounds **M6**, **M9**, **M12,** and **M14** are commercially available and used in **P7**, **P11** and **P12** in laboratory-scale synthesis. Materials in light gray letters are below the LCA cut-off of 10 mol%; therefore, they are not considered in the impact calculations. A detailed list of all solvents and reactants used can be found in Table S3. The red stars highlight the environmentally most critical process steps as identified by the ESR-method and set threshold. Abbreviations are: ACN = acetonitrile, dba = dibenzylideneacetone, DMC = dimethyl carbonate, DMM = dimethoxymethane, DMSO = dimethyl sulfoxide, Me = methyl, Et = ethyl, TBAB = tetrabutylammonium bromide, and $^t$Bu = *tert*-butyl.

# Chapter S2b) **TPA** Overview process details

## Table S3 Overview process details

**Table S3**: Process steps with detailed information for the synthesis of the target compounds **TPA-0, TPA-1** and **TPA-2** from compounds that are available in the EcoInvent database (yellow). Synthesis steps (**P7**, **P11**, **P12** and **P14**) which were performed in our laboratory are highlighted in dark grey, the other reaction procedures were adapted from the corresponding literature (see references given in the table). The light grey colour visualizes, which reaction step has been applied to obtain **TPA-0, TPA-1,** or **TPA-2**. The process steps **P1 - P7** include the formation of the polymerizable arm and are highlighted in pink, in the process steps **P8 - P15** (green) the redox active core is formed, in the process steps **P12 - P15** (blue) metal catalysts are involved. The reaction step **P15*** is shown to compare different synthesis strategies regarding their environmental impact.

| P# | 0 | 1 | 2 | Educt | Reactant(s) | Solvent | Cp / $^{J}/_{mol \cdot K}$ | Reaction conditions | Product (state) | Byproduct(s) | Yield [%] | Reference |
|---|---|---|---|---|---|---|---|---|---|---|---|---|
| **P1** | | | | M1 | $(NH_4)_2S_2O_8$[11] (APS) | $H_2O$ | 75 | 50 °C for 1 h | M2 (liquid) | $NH_4SO_4$ | 88 | Imanzadeh et al. 2007[12] |
| **P2** | | | | M2 | 3x $CH_2O$, NaOH | $H_2O$ | 75 | 58 °C for 1 h[13] | M3 (solid) | $HCOO^-$, $Na^+$ | 90 | Werle et al. 2000[14] |
| **P3** | | | | M4 | $Na^+EtO^-$ | EtOH | 121 | transesterification with distillation 180 °C for 7 h | M5 (liquid) | ethylene glycol | 91 | Qiu et al. 2012[15] |
| **P4** | | | | M3, M5 | KOH | EtOH | 121 | 110 °C for 1 h, distillation | M6 (liquid) | $CO_2$ | 87 | Pattison 1957[16] |
| **P5** | | | | M7 | $LiAlH_4$ | DMM[17] | 164 | 42°C for 1 h, cooling to r.t. | M8 (solid) | LiOH, $Al(OH)_3$, $H_2$ | 78 | Bernatek 1954[18] |
| **P6** | | | | M8 | HBr | $H_2O$ | 75 | 100 °C for 24 h | M9 (liquid) | $H_2O$ | 97 | De, Aswal et al. 2010[19] |
| **P7** | | | | M6, M9 | $NaOH_{aq.}$, TBAB | n-$C_6H_{14}$ | 198 | 75°C for 6 h | M10 (liquid) | HBr | 99 | Schuller 2025[8] |
| **P8** | | | | M11 | $Br_2$ | $CS_2$ | 76 | 5 °C for 2 h, distillation | M12 (solid) | HBr | 80 | Adams & Marvel 1921[20] |
| **P9** | | | | M12 | 1,3-DMI-2-$CO_2$, DMC | ACN | 92 | 160 °C for 2 h, microwave reactor | M13 (liquid) | MeOH, $CO_2$ | 100 | Lui et al. 2016[21] |
| **P10** | | | | M13 | $NH_3$, Cu | $H_2O$ | 75 | autoclave, 200 °C for 2 h | M14 (solid) | HBr | 91 | Wang et al. 2013[22] |

| P# | 0 | 1 | 2 | Educt | Reactant(s) | Solvent | Cp / $^{J}/_{mol \cdot K}$ | Reaction conditions | Product (state) | Byproduct(s) | Yield [%] | Reference |
|---|---|---|---|---|---|---|---|---|---|---|---|---|
| **P11** | | | | M10, M12 | $K_2CO_3$ | DMSO | 149 | 65 °C for 4 h | M15 (liquid) | HBr | 97 | Jungermann et al. 2006[7] |
| **P12** | | | | M14, 2x M15 | $Pd_2dba_3$ (0.01 eq.), $P^tBu_3$, $NaO^tBu$ | Toluene | 157 | 75 °C, over night | **TPA-2 (liquid)** | HBr, Pd cat. | 96 | Plein & Gatzka 2025[9] |
| **P13** | | | | M13, M14 | $Pd_2dba_3$ (0.005 eq.), $P^tBu_3$, $NaO^tBu$ | Toluene | 157 | 90 °C for 1 h | M17 (solid) | HBr, Pd cat. | 95 | Saint-Louis et al. 2021[23] |
| **P14** | | | | M15, M17 | $Pd_2dba_3$ (0.01 eq.), $P^tBu_3$, $NaO^tBu$ | Toluene | 157 | 70 °C for 72 h | **TPA-1 (liquid)** | HBr, Pd cat. | 80 | Plein & Gatzka 2025[9] |
| **P15** | | | | 2x M13, M14 | $Pd_2dba_3$ (0.02 eq.), $P^tBu_2$-*o*-diphenyl, $NaO^tBu$ | Toluene | 157 | 100 °C for 15 h | **TPA-0 (solid)** | HBr, Pd cat | 95 | Stephens et al. 2008[24] |
| **P15*** | | | | 2x M13, M14 | CuI (0.035 eq.), $PBu_3$, $KO^tBu$[25] | Toluene | 157 | 135 °C for 10.5 h | **TPA-0 (solid)** | HBr, Cu Kat. | 87 | Chaudhari et al. 2003[26] |

## Chapter S2c) **TPA** Life Cycle Inventory

### Table S4a: LCI of P1a - synthesis of ammonium persulfate (reactant)

| **Inputs** | **Dataset** | **Amount** | **$x_i$(TPA-0)** | **$x_i$(TPA-0*)** | **$x_i$(TPA-1)** | **$x_i$(TPA-2)** | **Unit** |
|---|---|---|---|---|---|---|---|
| Sulfuric acid | market for sulfuric acid | 0.578 | | | 0.905 | 1.07 | kg |
| Ammonium sulfate | market for ammonium sulfate | 0.429 | | | 0.671 | 0.797 | kg |
| Electricity, low voltage | market for electricity | 7.20 | | | 11.3 | 13.4 | MJ |
| **Outputs** | **Dataset** | **Amount** | | | | | **Unit** |
| Ammonium persulfate | P1a: Synthesis of ammonium persulfate | 1.00 | | | 1.57 | 1.86 | kg |

### Table S4b: LCI of P1b - Synthesis of Butanal (M2)

| **Inputs** | **Dataset** | **Amount** | **$x_i$(TPA-0)** | **$x_i$(TPA-0*)** | **$x_i$(TPA-1)** | **$x_i$(TPA-2)** | **Unit** |
|---|---|---|---|---|---|---|---|
| 1-butanol (M1) | market for 1-butanol | 1.17 | | | 0.340 | 0.403 | *kg* |
| *Ammonium persulfate* | *P1a: Synthesis of ammonium persulfate* | 5.39 | | | 1.57 | 1.86 | *kg* |
| Water, deionized | market for water | 0.268 | | | 0.078 | 0.092 | kg |
| Electricity, low voltage | market for electricity | 0.324 | | | 0.094 | 0.111 | MJ |
| **Outputs** | **Dataset** | **Amount** | | | | | **Unit** |
| Butanal (M2) | P1: Synthesis of butanal | 1.00 | | | 0.290 | 0.344 | kg |
| Waste | Treatment of spent solvent mixture, hazardous waste incineration | 5.83 | | | 1.69 | 2.01 | kg |

Table S4c: LCI of P2 - Synthesis of 1,1,1 Trimethylol propane (M3)

| Inputs | Dataset | Amount | $x_i$(TPA-0) | $x_i$(TPA-0*) | $x_i$(TPA-1) | $x_i$(TPA-2) | Unit |
|---|---|---|---|---|---|---|---|
| *Butanal (M2)* | *P1: Synthesis of butanal* | *0.597* | | | *0.290* | *0.344* | *kg* |
| Formaldehyde | market for formaldehyde | 0.747 | | | 0.363 | 0.431 | *kg* |
| Sodium hydroxide, without water, in 50% solution state | market for sodium hydroxide, without water, in 50% solution state | 0.332 | | | 0.161 | 0.192 | kg |
| Water, deionized | market for water, deionized | 0.460 | | | 0.224 | 0.265 | kg |
| Electricity, low voltage | market for electricity | 0.157 | | | 0.076 | 0.089 | MJ |
| **Outputs** | **Dataset** | **Amount** | | | | | **Unit** |
| 1,1,1 Trimethylolpropane (M3) | P2: Synthesis of 1,1,1 Trimethylolpropane (M3) | 1.00 | | | 0.486 | 0.577 | kg |
| Waste | Treatment of spent solvent mixture, hazardous waste incineration | 1.14 | | | 0.552 | 0.655 | kg |

Table S4d: LCI of P3 - Synthesis of Diethyl carbonate (M5)

| **Inputs** | **Dataset** | **Amount** | **$x_i$(TPA-0)** | **$x_i$(TPA-0*)** | **$x_i$(TPA-1)** | **$x_i$(TPA-2)** | **Unit** |
|---|---|---|---|---|---|---|---|
| Ethylene Carbonate (M4) | market for ethylene carbonate | 0.820 | | | 0.331 | 0.393 | *kg* |
| Ethanol | market for ethanol, without water, in 99.7% solution state | 1.55 | | | 0.629 | 0.746 | *kg* |
| Sodium ethoxide | market for sodium ethoxide | 0.110 | | | 0.044 | 0.053 | kg |
| Electricity, low voltage | market for electricity | 2.36 | | | 0.954 | 1.10 | MJ |
| **Outputs** | **Dataset** | **Amount** | | | | | **Unit** |
| Diethyl carbonate (M5) | P3: Synthesis of Diethyl carbonate (M5) | 1.00 | | | 0.404 | 0.479 | kg |
| Waste | Treatment of spent solvent mixture, hazardous waste incineration | 1.49 | | | 0.601 | 0.713 | kg |

Table S4e: LCI of P4 - Synthesis of 3-Ethyl-3-hydroxymethyloxetane (M6)

| **Inputs** | **Dataset** | **Amount** | **$x_i$(TPA-0)** | **$x_i$(TPA-0*)** | **$x_i$(TPA-1)** | **$x_i$(TPA-2)** | **Unit** |
|---|---|---|---|---|---|---|---|
| *1,1,1 Trimethylolpropane (M3)* | *P2: Synthesis of 1,1,1 Trimethylolpropane (M3)* | *1.46* | | | *0.486* | *0.577* | *kg* |
| *Diethyl carbonate (M5)* | *P3: Synthesis of Diethyl carbonate (M5)* | *1.21* | | | *0.404* | *0.479* | *kg* |
| Potassium Hydroxide | market for potassium hydroxide | 0.005 | | | 0.002 | 0.002 | kg |
| Ethanol | market for ethanol, without water, in 99.7% solution state | 0.041 | | | 0.014 | 0.016 | kg |
| Electricity, low voltage | market for electricity | 0.131 | | | 0.044 | 0.049 | MJ |
| **Outputs** | **Dataset** | **Amount** | | | | | **Unit** |
| 3-Ethyl-3-hydroxy-methyloxetane (M6) | P4: Synthesis of 3-Ethyl-3-hydroxy-methyloxetane (M6) | 1.00 | | | 0.486 | 0.395 | kg |
| Waste | Treatment of spent solvent mixture, hazardous waste incineration | 1.71 | | | 0.404 | 0.678 | kg |

Table S4f: LCI of P5a - Synthesis of Dimethoxymethane (DMM)

| **Inputs** | **Dataset** | **Amount** | **$x_i$(TPA-0)** | **$x_i$(TPA-0*)** | **$x_i$(TPA-1)** | **$x_i$(TPA-2)** | **Unit** |
|---|---|---|---|---|---|---|---|
| Methanol | market for methanol | 0.859 | | | 32.3 | 38.3 | kg |
| Formaldehyde | market for formaldehyde | 0.403 | | | 15.1 | 17.9 | kg |
| Water, deionized | market for water, deionized | 0.976 | | | 36.7 | 43.5 | kg |
| Electricity, low voltage | market for electricity | 1.02 | | | 38.2 | 44.1 | MJ |
| **Outputs** | **Dataset** | **Amount** | | | | | **Unit** |
| Dimethoxymethane | P5a: Synthesis of Dimethoxymethane | 1.00 | | | 37.6 | 44.6 | kg |
| Waste | Treatment of spent solvent mixture, hazardous waste incineration | 1.24 | | | 46.5 | 55.2 | kg |

Table S4g: LCI of P5 - Synthesis of Hexane-1,6-diol (M8)

| **Inputs** | **Dataset** | **Amount** | **$x_i$(TPA-0)** | **$x_i$(TPA-0*)** | **$x_i$(TPA-1)** | **$x_i$(TPA-2)** | **Unit** |
|---|---|---|---|---|---|---|---|
| Adipic Acid (M7) | market for adipic acid | 1.59 | | | 1.67 | 1.98 | kg |
| Lithium | market for lithium | 0.228 | | | 0.240 | 0.284 | kg |
| Aluminum | market for aluminum, primary, ingot | 0.880 | | | 0.925 | 1.10 | kg |
| Hydrogen, gaseous | market for hydrogen, gaseous | 0.130 | | | 0.137 | 0.162 | kg |
| *Dimethoxymethane* | *P5a: Synthesis of Dimethoxymethane* | *35.7* | | | *37.6* | *44.6* | *kg* |
| Electricity, low voltage | market for electricity | 0.143 | | | 0.151 | 0.178 | MJ |
| **Outputs** | **Dataset** | **Amount** | | | | | **Unit** |
| Hexane-1,6-diol (M8) | P5: Synthesis of Hexane-1,6-diol (M8) | 1.00 | | | 1.05 | 1.25 | kg |
| Waste | Treatment of spent solvent mixture, hazardous waste incineration | 1.82 | | | 1.92 | 2.27 | kg |

Table S4h: LCI of P6 - Synthesis of Hexane-1,6-dibromide (M9)

| **Inputs** | **Dataset** | **Amount** | **$x_i$(TPA-0)** | **$x_i$(TPA-0*)** | **$x_i$(TPA-1)** | **$x_i$(TPA-2)** | **Unit** |
|---|---|---|---|---|---|---|---|
| *1,6 Hexane diol (M8)* | *P5: Synthesis of Hexane-1,6-diol (M8)* | *0.499* | | | *1.05* | *1.25* | *kg* |
| Bromine (as molar equivalent for HBr) | market for bromine | 2.74 | | | 5.78 | 6.86 | kg |
| Hydrogen, gaseous | market for hydrogen, gaseous | 0.034 | | | 0.072 | 0.085 | kg |
| Water, deionized | market for water, deionized | 2.91 | | | 6.13 | 7.28 | kg |
| Electricity, low voltage | market for electricity | 3.03 | | | 6.38 | 7.30 | MJ |
| **Outputs** | **Dataset** | **Amount** | | | | | **Unit** |
| Hexane-1,6-dibromide (M9) | P6: Synthesis of Hexane-1,6-dibromide (M9) | 1.00 | | | 2.11 | 2.50 | kg |
| Waste | Treatment of spent solvent mixture, hazardous waste incineration | 5.19 | | | 10.9 | 13.0 | kg |

Table S4i: LCI of P7 - Synthesis of 3-(6-Bromohexyloxymethyl)-3-ethyloxetane (M10)

| **Inputs** | **Dataset** | **Amount** | **$x_i$(TPA-0)** | **$x_i$(TPA-0*)** | **$x_i$(TPA-1)** | **$x_i$(TPA-2)** | **Unit** |
|---|---|---|---|---|---|---|---|
| *Hexane-1,6-dibromide (M9)* | *P6: Synthesis of Hexane-1,6-dibromide (M9)* | *2.65* | | | *2.11* | *2.50* | *kg* |
| *3-Ethyl-3-hydroxymethyloxetane (M6)* | *P4: Synthesis of 3-Ethyl-3-hydroxy-methyloxetane (M6)* | *0.420* | | | *0.334* | *0.396* | *kg* |
| Sodium hydroxide | market for sodium hydroxide, without water, in 50% solution state | 1.63 | | | 1.29 | 1.54 | kg |
| *Tetra butyl ammonium bromine* | *Not considered* | *0.006* | | | *0.005* | *0.006* | *kg* |
| Hexane | market for hexane | 6.58 | | | 5.22 | 6.20 | kg |
| Electricity, low voltage | market for electricity | 1.88 | | | 1.49 | 1.72 | MJ |
| **Outputs** | **Dataset** | **Amount** | | | | | **Unit** |
| 3-(6-Bromohexyloxymethyl)-3-ethyloxetane (M10) | P7: Synthesis of 3-(6-Bromohexyloxymethyl)-3-ethyloxetane (M10) | 1.00 | | | 0.794 | 0.942 | kg |
| Waste | Treatment of spent solvent mixture, hazardous waste incineration | 10.3 | | | 8.12 | 9.69 | kg |

Table S4j: LCI of P8 - Synthesis of Phenol-4-bromid (M12)

| **Inputs** | **Dataset** | **Amount** | **$x_i$(TPA-0)** | **$x_i$(TPA-0*)** | **$x_i$(TPA-1)** | **$x_i$(TPA-2)** | **Unit** |
|---|---|---|---|---|---|---|---|
| Phenol (M11) | market for phenol | 0.679 | 1.33 | 1.29 | 0.967 | 0.634 | *kg* |
| Bromine | market for bromine | 1.16 | 2.26 | 2.19 | 1.65 | 1.08 | *kg* |
| Carbon Disulfide | market for carbon disulfide | 1.97 | 1.28 | 1.24 | 0.935 | 0.612 | kg |
| Electricity, low voltage | market for electricity | 0.256 | 0.161 | 0.16 | 0.122 | 0.084 | MJ |
| **Outputs** | **Dataset** | **Amount** | | | | | **Unit** |
| Phenol-4-bromid (M12) | P8: Synthesis of Phenol-4-bromid (M12) | 1.00 | 1.96 | 1.89 | 1.42 | 0.933 | kg |
| Waste | Treatment of spent solvent mixture, hazardous waste incineration | 4.47 | 2.92 | 2.82 | 2.12 | 1.39 | kg |

Table S4k: LCI of P9 - Synthesis of Benzene (1-methoxy, 4-bromid) (M13)

| **Inputs** | **Dataset** | **Amount** | **$x_i$(TPA-0)** | **$x_i$(TPA-0*)** | **$x_i$(TPA-1)** | **$x_i$(TPA-2)** | **Unit** |
|---|---|---|---|---|---|---|---|
| *Phenol-4-bromid (M12)* | *P8: Synthesis of Phenol-4-bromid (M12)* | *0.925* | *1.96* | *1.89* | *0.878* | *0.284* | *kg* |
| Dimethyl carbonate (DMC) | market for dimethyl carbonate | 6.34 | 13.4 | 13.0 | 6.16 | 1.95 | kg |
| *1,3 Dimethyl Imidazolium 2-carboxylate* | *Not considered* | *0.075* | *0.158* | *0.153* | *0.071* | *0.023* | *kg* |
| Acetonitrile | market for acetonitrile | 4.74 | 5.01 | 4.85 | 2.25 | 0.728 | kg |
| Electricity, low voltage | market for electricity | 6.13 | 6.15 | 5.97 | 2.91 | 1.03 | MJ |
| **Outputs** | **Dataset** | **Amount** | | | | | **Unit** |
| Benzene (1-methoxy, 4-bromid) (M13) | P9: Synthesis of Benzene (1-methoxy, 4-bromid) (M13) | 1.00 | 2.11 | 2.05 | 0.949 | 0.307 | kg |
| Waste | Treatment of spent solvent mixture, hazardous waste incineration | 17.4 | 18.4 | 17.8 | 8.26 | 2.68 | kg |

Table S4l: LCI of P10 - Synthesis of 4-Anisidin (M14)

| **Inputs** | **Dataset** | **Amount** | **$x_i$(TPA-0)** | **$x_i$(TPA-0*)** | **$x_i$(TPA-1)** | **$x_i$(TPA-2)** | **Unit** |
|---|---|---|---|---|---|---|---|
| *Benzene (1-methoxy, 4-bromid) (M13)* | *P9: Synthesis of Benzene (1-methoxy, 4-bromid) (M13)* | *1.67* | *0.646* | *0.703* | *0.482* | *0.307* | *kg* |
| Iodine | market for iodine | 0.113 | 0.044 | 0.047 | 0.035 | 0.021 | kg |
| Copper | market for copper, cathode | 0.056 | 0.022 | 0.024 | 0.018 | 0.010 | kg |
| Ammonia (anhydrous) liquid | market for ammonia | 0.455 | 0.176 | 0.192 | 0.142 | 0.084 | kg |
| Water, deionized | market for water, deionized | 0.878 | 0.340 | 0.370 | 0.273 | 0.162 | kg |
| Electricity, low voltage | market for electricity | 6.33 | 2.36 | 2.50 | 1.970 | 1.388 | MJ |
| **Outputs** | **Dataset** | **Amount** | | | | | **Unit** |
| 4-Anisidin (M14) | P10: Synthesis of 4-Anisidin (M14) | 1.00 | 0.387 | 0.421 | 0.311 | 0.184 | kg |
| Waste | Treatment of spent solvent mixture, hazardous waste incineration | 2.05 | 0.840 | 0.914 | 0.639 | 0.340 | kg |

Table S4m: LCI of P11 - Synthesis of 3-(6-(4-Bromophenyl)oxyhexyloxymethyl)-3-ethyloxetane (M15)

| **Inputs** | **Dataset** | **Amount** | **$x_i$(TPA-0)** | **$x_i$(TPA-0*)** | **$x_i$(TPA-1)** | **$x_i$(TPA-2)** | **Unit** |
|---|---|---|---|---|---|---|---|
| *3-(6-Bromohexyloxymethyl)-3-ethyloxetane (M10)* | *P7: Synthesis of 3-(6-Bromohexyloxymethyl)-3-ethyloxetane (M10)* | *0.810* | | | *0.794* | *0.942* | *kg* |
| *Phenol-4-bromid (M12)* | *P8: Synthesis of Phenol-4-bromid (M12)* | *0.558* | | | *0.547* | *0.649* | *kg* |
| Potassium carbonate | market for potassium carbonate | 0.891 | | | 0.873 | 1.04 | kg |
| Dimethyl sulfoxide | market for dimethyl sulfoxide | 7.16 | | | 7.02 | 8.33 | kg |
| Electricity,low voltage | market for electricity | 1.00 | | | 0.99 | 1.15 | MJ |
| **Outputs** | **Dataset** | **Amount** | | | | | **Unit** |
| 3-(6-(4-Bromophenyl) oxyhexyl-oxymethyl)-3-ethyloxetane (M15) | P11: Synthesis of 3-(6-(4-Bromophenyl) oxyhexyl-oxymethyl)-3-ethyloxetane (M15) | 1.00 | | | 0.980 | 1.16 | kg |
| Waste | Treatment of spent solvent mixture, hazardous waste incineration | 8.42 | | | 8.25 | 9.79 | kg |

Table S4n: LCI of P12 - Synthesis of TPA-2

| **Inputs** | **Dataset** | **Amount** | **$x_i$(TPA-0)** | **$x_i$(TPA-0*)** | **$x_i$(TPA-1)** | **$x_i$(TPA-2)** | **Unit** |
|---|---|---|---|---|---|---|---|
| *4-Anisidin (M14)* | *P10: Synthesis of 4-Anisidin (M14)* | *0.184* | | | | *0.184* | *kg* |
| *3-(6-(4-Bromophenyl) oxyhexyl-oxymethyl)-3-ethyloxetane (M15)* | *P11: Synthesis of 3-(6-(4-Bromophenyl) oxyhexyl-oxymethyl)-3-ethyloxetane (M15)* | *1.16* | | | | *1.16* | *kg* |
| Sodium | market for sodium | 0.085 | | | | 0.085 | kg |
| 2-butanol (molar equivalent of tert-butylat) | market for 2-butanol | 0.274 | | | | 0.274 | kg |
| Palladium (molar equivalent of Pd2dba3) | market for palladium | 0.002 | | | | 0.002 | kg |
| *Tri-tertbutyl phosphine* | *Not considered* | *0.005* | | | | *0.005* | *kg* |
| Toluene | market for toluene | 1.64 | | | | 1.64 | kg |
| Electricity, low voltage | market for electricity | 0.936 | | | | 0.936 | MJ |
| **Outputs** | **Dataset** | **Amount** | | | | | **Unit** |
| TPA-2 (M18) | P12: Synthesis of TPA-1 (M18) | 1.00 | | | | 1.00 | kg |
| Waste | Treatment of spent solvent mixture, hazardous waste incineration | 2.35 | | | | 2.35 | kg |

Table S4o: LCI of P13 - Synthesis of 4,4′-Dimethoxydiphenylamine (M17)

| **Inputs** | **Dataset** | **Amount** | **$x_i$(TPA-0)** | **$x_i$(TPA-0*)** | **$x_i$(TPA-1)** | **$x_i$(TPA-2)** | **Unit** |
|---|---|---|---|---|---|---|---|
| *4-Anisidin (M14)* | *P10: Synthesis of 4-Anisidin (M14)* | *0.566* | | | *0.311* | | *kg* |
| Benzene (1-methoxy, 4-bromid) (M13) | P9: Synthesis of Benzene (1-methoxy, 4-bromid) (M13) | *0.848* | | | *0.466* | | *kg* |
| Sodium | market for sodium | 0.158 | | | 0.087 | | kg |
| 2-butanol (molar equivalent of tert-butylat) | market for 2-butanol | 0.504 | | | 0.277 | | kg |
| Palladium (molar equivalent of Pd2dba3) | market for palladium | 0.003 | | | 0.002 | | kg |
| *Tri-tertbutyl phosphine* | *Not considered* | *0.005* | | | *0.003* | | *kg* |
| Toluene | market for toluene | 3.15 | | | 1.73 | | kg |
| Electricity, low voltage | market for electricity | 0.633 | | | 0.348 | | MJ |
| **Outputs** | **Dataset** | **Amount** | | | | | **Unit** |
| 4,4′-Dimethoxydiphenylamine (M17) | P13: Synthesis of 4,4′-Dimethoxydiphenylamine (M17) | 1.00 | | | 0.550 | | kg |
| Waste | Treatment of spent solvent mixture, hazardous waste incineration | 4.23 | | | 2.33 | | kg |

Table S4p: LCI of P14 - Synthesis of TPA-1

| **Inputs** | **Dataset** | **Amount** | **$x_i$(TPA-0)** | **$x_i$(TPA-0*)** | **$x_i$(TPA-1)** | **$x_i$(TPA-2)** | **Unit** |
|---|---|---|---|---|---|---|---|
| *4,4'-Dimethoxydiphenylamine (M17)* | *P13: Synthesis of 4,4'-Dimethoxydiphenylamine (M17)* | *0.550* | | | *0.550* | | *kg* |
| *3-(6-(4-Bromophenyl)oxy hexyl-oxymethyl)-3-ethyloxetane (M15)* | *P11: Synthesis of 3-(6-(4-Bromophenyl) oxyhexyl-oxymethyl)-3-ethyloxetane (M15)* | *0.980* | | | *0.980* | | *kg* |
| Sodium | market for sodium | 0.069 | | | 0.069 | | kg |
| 2-butanol (molare equivalent of tert-butylat) | market for 2-butanol | 0.219 | | | 0.219 | | kg |
| Palladium (molar equivalent of Pd2(dba)3) | market for palladium | 0.005 | | | 0.005 | | kg |
| *Tri-tertbutyl phosphine* | *Not considered* | *0.008* | | | *0.008* | | *kg* |
| Toluene | market for toluene | 2.13 | | | 2.13 | | kg |
| Electricity, low voltage | market for electricity | 3.82 | | | 4.61 | | MJ |
| **Outputs** | **Dataset** | **Amount** | | | | | **Unit** |
| TPA-1 (M18) | P14: Synthesis of TPA-1 (M18) | 1.00 | | | 1.00 | | kg |
| Waste | Treatment of spent solvent mixture, hazardous waste incineration | 2.96 | | | 2.96 | | kg |

Table S4q: LCI of P15 - Synthesis of TPA-0

| **Inputs** | **Dataset** | **Amount** | **$x_i$(TPA-0)** | **$x_i$(TPA-0*)** | **$x_i$(TPA-1)** | **$x_i$(TPA-2)** | **Unit** |
|---|---|---|---|---|---|---|---|
| *4-Anisidin (M14)* | *P10: Synthesis of 4-Anisidin (M14)* | *0.387* | *0.387* | | | | *kg* |
| *Benzene (1-methoxy, 4-bromid) (M13)* | *P9: Synthesis of (M13)* | *1.47* | *1.47* | | | | *kg* |
| Sodium | market for sodium | 0.180 | 0.180 | | | | kg |
| 2-butanol (molar equivalent of tert-butylat) | market for 2-butanol | 0.582 | 0.582 | | | | kg |
| Palladium (molar equivalent of Pd2dba3) | market for palladium | 0.013 | 0.013 | | | | kg |
| *PtBu2-o-diphenyl* | *Not considered* | *0.050* | *0.050* | | | | *kg* |
| Toluene | market for toluene | 1.36 | 1.36 | | | | kg |
| Electricity, low voltage | market for electricity | 1.47 | 1.47 | | | | MJ |
| **Outputs** | **Dataset** | **Amount** | | | | | **Unit** |
| TPA-0 (M18) | P15: Synthesis of TPA-0 (M18) | 1.00 | 1.00 | | | | kg |
| Waste | Treatment of spent solvent mixture, hazardous waste incineration | 3.04 | 3.04 | | | | kg |

Table S4r: LCI of P15a* - Synthesis of Kalium-tert-butanolat (reactant)

| Inputs | Dataset | Amount | $x_i$(TPA-0) | $x_i$(TPA-0*) | $x_i$(TPA-1) | $x_i$(TPA-2) | Unit |
|---|---|---|---|---|---|---|---|
| 2-butanol (molare equivalent of tert-butylat) | market for 2-butanol | 3.12 | | 3.59 | | | kg |
| Potassium hydroxide | market for potassium hydroxide | 0.505 | | 0.581 | | | kg |
| Water, deionized | market for water, deionized | 0.499 | | 0.574 | | | kg |
| Electricity, low voltage | market for electricity | 1.18 | | 1.35 | | | MJ |
| **Outputs** | **Dataset** | **Amount** | | | | | **Unit** |
| $KO^tBu$ | P15s*: Synthesis of $KO^tBu$ | 1.00 | | 1.15 | | | kg |
| Waste | Treatment of spent solvent mixture, hazardous waste incineration | 3.12 | | 3.59 | | | kg |

Table S4s: LCI of P15b* - Synthesis of TPA-0*

| **Inputs** | **Dataset** | **Amount** | **$x_i$(TPA-0)** | **$x_i$(TPA-0*)** | **$x_i$(TPA-1)** | **$x_i$(TPA-2)** | **Unit** |
|---|---|---|---|---|---|---|---|
| *4-Anisidine (M14)* | *P10: Synthesis of 4-Anisidine (M14)* | *0.421* | | *0.421* | | | *kg* |
| *Benzene (1-methoxy, 4-bromid) (M13)* | *P9: Synthesis of (M13)* | *1.35* | | *1.35* | | | *kg* |
| *Potassium-tert-butoxide* | *P15a*: Synthesis of Potassium-tert-butoxide* | *1.15* | | *1.15* | | | *kg* |
| Copper | market for copper, cathode | 0.008 | | 0.008 | | | kg |
| Iodine | market for iodine | 0.016 | | 0.016 | | | kg |
| *PBu3* | *Not considered* | *0.049* | | *0.049* | | | *kg* |
| Toluene | market for toluene | 0.697 | | 0.697 | | | kg |
| Electricity, low voltage | market for electricity | 1.46 | | 0.146 | | | MJ |
| **Outputs** | **Dataset** | **Amount** | | | | | **Unit** |
| TPA-0* (M18) | P13: Synthesis of TPA-0 (M18) | 1.00 | | 1.00 | | | kg |
| Waste | Treatment of spent solvent mixture, hazardous waste incineration | 2.69 | | 2.69 | | | kg |

# Chapter S3) Original data for the synthesis of **TPA** and **NMCxyz**

## Table S5: Specific data for the synthesis of **TPA.**

**Table S5**: Specific data for the synthesis of **TPA-0**, **TPA-1** and **TPA-2** from the EcoInvent database.

| Category | TAP | GWP 1000 | FETP | METP | TETP | FFP | FEP | MEP | HTPc | HTPnc | IRP | LOP | SOP | ODP infinite | PMFP | HOFP | EOFP | WCP |
|---|---|---|---|---|---|---|---|---|---|---|---|---|---|---|---|---|---|---|
| **Unit** | kg SO2-Eq | kg CO2-Eq | kg 1,4-DCB-Eq | kg 1,4-DCB-Eq | kg 1,4-DCB-Eq | kg oil-Eq | kg P-Eq | kg N-Eq | kg 1,4-DCB-Eq | kg 1,4-DCB-Eq | kBq Co-60-Eq | m2*a crop-Eq | kg Cu-Eq | kg CFC-11-Eq | kg PM2.5-Eq | kg NOx-Eq | kg NOx-Eq | m3 |
| 1-Butanol | | | | | | | | | | | | | | | | | | |
| 2-Butanol | | | | | | | | | | | | | | | | | | |
| Acetonitrile | | | | | | | | | | | | | | | | | | |
| Adipic acid | | | | | | | | | | | | | | | | | | |
| Aluminum | | | | | | | | | | | | | | | | | | |
| Ammonia | | | | | | | | | | | | | | | | | | |
| Ammonium sulfate | | | | | | | | | | | | | | | | | | |
| Bromine | | | | | | | | | | | | | | | | | | |
| Carbon disulfide | | | | | | | | | | | | | | | | | | |
| Chlorine | | | | | | | | | | | | | | | | | | |
| Copper, cathode | | | | | | | | | | | | | | | | | | |
| Dichloromethane | | | | | | | | | | | | | | | | | | |
| Dimethoxymethane | | | | | | | | | | | | | | | | | | |
| Dimethyl-sulfoxide | | | | | | | | | | | | | | | | | | |
| Dimethyl carbonate | | | | | | | | | | | | | | | | | | |
| Ethanol | | | | | | | | | | | | | | | | | | |
| Ethylene carbonate | | | | | | | | | | | | | | | | | | |
| Formaldehyde | | | | | | | | | | | | | | | | | | |
| Hydrogen | | | | | | | | | | | | | | | | | | |
| Hydrogen bromide | | | | | | | | | | | | | | | | | | |

Raw data cannot be published here. Licence from EcoInvent required.

| Category | TAP | GWP 1000 | FETP | METP | TETP | FFP | FEP | MEP | HTPc | HTPnc | IRP | LOP | SOP | ODP infinite | PMFP | HOFP | EOFP | WCP |
|---|---|---|---|---|---|---|---|---|---|---|---|---|---|---|---|---|---|---|
| Iod | | | | | | | | | | | | | | | | | | |
| Lithium | | | | | | | | | | | | | | | | | | |
| Methanol | | | | | | | | | | | | | | | | | | |
| n-hexane | | | | | | | | | | | | | | | | | | |
| Oxalyl acid | | | | | | | | | | | | | | | | | | |
| Palladium | | | | | | | | | | | | | | | | | | |
| Phenol | | | | | | | | | | | | | | | | | | |
| Potassium carbonate | | | | | | | | | | | | | | | | | | |
| Potassium hydroxide | | | | | | | | | | | | | | | | | | |
| Sodium hydroxide | | | | | | | | | | | | | | | | | | |
| Sodium (Na) | | | | | | | | | | | | | | | | | | |
| Sodium ethoxide | | | | | | | | | | | | | | | | | | |
| Sulfuric acid | | | | | | | | | | | | | | | | | | |
| Toluene | | | | | | | | | | | | | | | | | | |
| Triethylamine | | | | | | | | | | | | | | | | | | |
| Water, deionized | | | | | | | | | | | | | | | | | | |
| Electricity | | | | | | | | | | | | | | | | | | |
| Waste | | | | | | | | | | | | | | | | | | |

Raw data cannot be published here. Licence from EcoInvent required.

# Chapter S4) Normalization factors

Normalization factors in ReCiPe2016 are based on measured global emissions and reflect their average contributions to the 18 environmental impact categories caused by one average individual somewhere on the world per year. These values expressed in the specific units of the respective MIC (e.g., kg $CO_2$-equivalents for GWP100) provide a benchmark for assessing the extent to which each category is influenced by per capita consumption. They serve as reference values to facilitate the evaluation of the significance and magnitude of specific environmental impacts in relation to global averages.

Table S6: Environmental scores per person of the world's population and year listed by ReCiPe 2016.[1,27,28]

| **MIC#** | **1** | **2** | **3** | **4** | **5** | **6** | **7** | **8** | **9** | **10** | **11** | **12** | **13** | **14** | **15** | **16** | **17** | **18** |
|---|---|---|---|---|---|---|---|---|---|---|---|---|---|---|---|---|---|---|
| **MIC** | **TAP** | **GWP 1000** | **FETP** | **METP** | **TETP** | **FFP** | **FEP** | **MEP** | **HTPc** | **HTPnc** | **IRP** | **LOP** | **SOP** | **ODP infinite** | **PMFP** | **HOFP** | **EOFP** | **WCP** |
| **Unit** | kg SO2 Eq | kg CO2 Eq | kg 1.4-DCB Eq | kg 1.4-DCB Eq | kg 1.4-DCB Eq | kg oil Eq | kg P Eq | kg N Eq | kg 1.4-DCB Eq | kg 1.4-DCB Eq | kBq Co-60 Eq | m2*a crop Eq | kg Cu Eq | kg CFC-11 Eq | kg PM2.5 Eq | kg NOx Eq | kg NOx Eq | m3 |
| **Normalization score N(MIC)** | 4.10 E+01 | 5.80 E+03 | 2.90 E+02 | 2.46 E+06 | 1.64 E+04 | 9.83 E+02 | 6.50 E-01 | 4.62 E+00 | 2.95 E+02 | 2.22 E+06 | 6.99 E+02 | 6.17 E+03 | 1.20 E+05 | 7.04 E-02 | 2.56 E+01 | 2.06 E+01 | 1.77 E+01 | 2.67 E+02 |

# Chapter S5) Environmental Matrix **EM** for **TPA**

## Table S7: **TPA-0**

**Table S7a**: **Environmental Matrix (EM):** Normalized impact values $V_{EM}$(MIC,P) according to Equation 2 (main text) for all process steps and MICs involved in the lab-scale synthesis of **TPA-0** (synthesized using Pd-cat. route; **P15**) using ReCiPe 2016 for functional unit of 1 kg material.

| Category | TAP | GWP 1000 | FETP | METP | TETP | FFP | FEP | MEP | HTPc | HTPnc | IRP | LOP | SOP | ODP infinite | PMFP | HOFP | EOFP | WCP |
|---|---|---|---|---|---|---|---|---|---|---|---|---|---|---|---|---|---|---|
| **P8** | 3.01E-03 | 4.08E-03 | 1.52E-03 | 9.89E-04 | 4.48E-03 | 7.52E-03 | 6.67E-03 | 1.02E-04 | 1.57E-01 | 9.17E-04 | 1.37E-03 | 3.94E-05 | 2.51E-06 | 1.05E-04 | 1.64E-03 | 1.76E-03 | 2.21E-03 | 4.16E-03 |
| **P9** | 4.88E-03 | 1.46E-02 | 7.90E-03 | 4.45E-03 | 1.45E-02 | 2.93E-02 | 2.75E-02 | 5.01E-03 | 6.77E-01 | 4.10E-03 | 4.59E-03 | 1.25E-04 | 6.13E-06 | 3.83E-04 | 2.83E-03 | 6.28E-03 | 7.89E-03 | 2.31E-03 |
| **P10** | 2.71E-04 | 4.72E-04 | 2.92E-03 | 1.22E-03 | 5.08E-03 | 4.07E-04 | 2.57E-03 | 1.98E-05 | 2.97E-02 | 1.13E-03 | 1.95E-04 | 5.95E-06 | 3.06E-06 | 1.79E-05 | 1.46E-04 | 1.62E-04 | 1.97E-04 | 6.10E-04 |
| **P15** | 5.41E-01 | 2.53E-02 | 2.44E-01 | 1.39E-01 | 4.73E-02 | 5.19E-02 | 2.66E-01 | 1.70E-03 | 4.27E+00 | 1.68E-01 | 1.59E-02 | 6.78E-04 | 2.36E-04 | 5.84E-03 | 2.52E-01 | 9.17E-02 | 1.09E-01 | 4.14E-03 |
| **$V_{EF}$** | 5.49E-01 | 4.45E-02 | 2.56E-01 | 1.45E-01 | 7.14E-02 | 8.91E-02 | 3.02E-01 | 6.83E-03 | 5.13E+00 | 1.74E-01 | 2.21E-02 | 8.48E-04 | 2.48E-04 | 6.35E-03 | 2.56E-01 | 9.99E-02 | 1.19E-01 | 1.12E-02 |

**Table S7b**: **Environmental Matrix (EM):** Normalized impact values $V_{EM}$(MIC,P) according to Equation 2 (main text) for all process steps and MICs involved in the lab-scale synthesis of **TPA-0** (synthesized using Cu-cat. route, **P15***) using ReCiPe 2016 for functional unit of 1 kg material.

| Category | TAP | GWP 1000 | FETP | METP | TETP | FFP | FEP | MEP | HTPc | HTPnc | IRP | LOP | SOP | ODP infinite | PMFP | HOFP | EOFP | WCP |
|---|---|---|---|---|---|---|---|---|---|---|---|---|---|---|---|---|---|---|
| **P8** | 3.01E-03 | 4.08E-03 | 1.52E-03 | 9.89E-04 | 4.48E-03 | 7.52E-03 | 6.67E-03 | 1.02E-04 | 1.57E-01 | 9.17E-04 | 1.37E-03 | 3.94E-05 | 2.51E-06 | 1.05E-04 | 1.64E-03 | 1.76E-03 | 2.21E-03 | 4.16E-03 |
| **P9** | 4.88E-03 | 1.46E-02 | 7.90E-03 | 4.45E-03 | 1.45E-02 | 2.93E-02 | 2.75E-02 | 5.01E-03 | 6.77E-01 | 4.10E-03 | 4.59E-03 | 1.25E-04 | 6.13E-06 | 3.83E-04 | 2.83E-03 | 6.28E-03 | 7.89E-03 | 2.31E-03 |
| **P10** | 2.71E-04 | 4.72E-04 | 2.92E-03 | 1.22E-03 | 5.08E-03 | 4.07E-04 | 2.57E-03 | 1.98E-05 | 2.97E-02 | 1.13E-03 | 1.95E-04 | 5.95E-06 | 3.06E-06 | 1.79E-05 | 1.46E-04 | 1.62E-04 | 1.97E-04 | 6.10E-04 |
| **P15*** | 1.27E-03 | 4.72E-03 | 3.20E-03 | 1.60E-03 | 5.64E-03 | 9.42E-03 | 8.11E-03 | 1.09E-04 | 1.56E-01 | 1.49E-03 | 1.21E-03 | 5.40E-05 | 3.48E-06 | 1.16E-04 | 7.47E-04 | 2.40E-03 | 3.40E-03 | 3.58E-03 |
| **$V_{EF}$** | 9.42E-03 | 2.39E-02 | 1.55E-02 | 8.26E-03 | 2.97E-02 | 4.66E-02 | 4.48E-02 | 5.24E-03 | 1.02E+00 | 7.63E-03 | 7.35E-03 | 2.24E-04 | 1.52E-05 | 6.21E-04 | 5.37E-03 | 1.06E-02 | 1.37E-02 | 1.07E-02 |

## Table S8: **TPA-1**

**Table S8**: **Environmental Matrix (EM):** Normalized impact values $V_{EM}$(MIC,P) according to Equation 2 (main text) for all process steps and MICs involved in the lab-scale synthesis of **TPA-1** (synthesized with literature values of Pd-cat. for **P13** and **P14**) using ReCiPe 2016 for functional unit of 1 kg material.

| Category | TAP | GWP 1000 | FETP | METP | TETP | FFP | FEP | MEP | HTPc | HTPnc | IRP | LOP | SOP | ODP infinite | PMFP | HOFP | EOFP | WCP |
|---|---|---|---|---|---|---|---|---|---|---|---|---|---|---|---|---|---|---|
| **P1** | 6.55E-04 | 1.13E-03 | 1.32E-03 | 6.96E-04 | 1.83E-03 | 1.39E-03 | 4.96E-03 | 2.26E-04 | 7.71E-02 | 6.45E-04 | 9.33E-04 | 1.42E-05 | 6.02E-07 | 4.49E-05 | 3.40E-04 | 3.88E-04 | 4.79E-04 | 2.33E-04 |
| **P2** | 4.73E-05 | 2.72E-04 | 1.08E-04 | 6.22E-05 | 1.88E-04 | 3.94E-04 | 5.10E-04 | 8.75E-06 | 1.09E-02 | 5.68E-05 | 1.02E-04 | 1.83E-06 | 3.28E-07 | 1.10E-05 | 3.62E-05 | 9.69E-05 | 1.20E-04 | 3.40E-05 |
| **P3** | 2.14E-04 | 4.12E-04 | 3.22E-04 | 1.48E-04 | 4.88E-04 | 5.43E-04 | 1.14E-03 | 3.09E-04 | 2.12E-02 | 1.37E-04 | 1.74E-04 | 1.61E-04 | 2.61E-07 | 1.48E-04 | 1.10E-04 | 2.32E-04 | 2.80E-04 | 3.91E-04 |
| **P4** | 1.35E-05 | 1.97E-04 | 1.75E-05 | 1.02E-05 | 2.64E-05 | 4.36E-05 | 2.68E-04 | 1.17E-05 | 2.22E-03 | 9.32E-06 | 1.49E-05 | 3.75E-06 | 5.20E-08 | 9.03E-06 | 8.86E-06 | 2.68E-05 | 3.21E-05 | 1.33E-05 |
| **P5** | 7.06E-03 | 2.85E-02 | 1.31E-02 | 7.67E-03 | 2.09E-02 | 5.23E-02 | 6.39E-02 | 2.77E-03 | 1.84E+00 | 6.90E-03 | 1.03E-02 | 2.15E-04 | 3.41E-04 | 7.15E-03 | 4.73E-03 | 1.17E-02 | 1.47E-02 | 3.51E-03 |
| **P6** | 2.20E-03 | 8.89E-03 | 2.59E-03 | 1.70E-03 | 8.35E-03 | 1.04E-02 | 1.45E-02 | 2.44E-04 | 2.64E-01 | 1.58E-03 | 2.35E-03 | 5.14E-05 | 5.71E-06 | 2.47E-04 | 1.40E-03 | 2.87E-03 | 3.53E-03 | 9.98E-03 |
| **P7** | 6.42E-04 | 3.59E-03 | 1.11E-03 | 6.90E-04 | 1.96E-03 | 5.84E-03 | 6.01E-03 | 1.68E-04 | 1.15E-01 | 6.32E-04 | 8.29E-04 | 2.42E-05 | 3.18E-06 | 1.34E-04 | 4.47E-04 | 1.47E-03 | 2.05E-03 | 4.06E-04 |
| **P8** | 2.19E-03 | 2.97E-03 | 1.11E-03 | 7.21E-04 | 3.27E-03 | 5.48E-03 | 4.86E-03 | 7.43E-05 | 1.15E-01 | 6.68E-04 | 9.95E-04 | 2.87E-05 | 1.83E-06 | 7.65E-05 | 1.20E-03 | 1.28E-03 | 1.61E-03 | 3.03E-03 |
| **P9** | 2.19E-03 | 6.58E-03 | 3.55E-03 | 2.00E-03 | 6.52E-03 | 1.31E-02 | 1.24E-02 | 2.25E-03 | 3.04E-01 | 1.84E-03 | 2.07E-03 | 5.60E-05 | 2.75E-06 | 1.72E-04 | 1.27E-03 | 2.82E-03 | 3.54E-03 | 1.04E-03 |
| **P10** | 4.81E-05 | 3.53E-04 | 6.72E-04 | 2.94E-04 | 2.21E-04 | 3.02E-04 | 1.16E-03 | 1.36E-05 | 1.33E-02 | 2.63E-04 | 1.41E-04 | 2.81E-06 | 2.29E-06 | 1.18E-05 | 3.23E-05 | 8.73E-05 | 1.07E-04 | 4.84E-04 |
| **P11** | 2.63E-03 | 4.72E-03 | 2.47E-03 | 1.38E-03 | 4.63E-03 | 7.76E-03 | 8.95E-03 | 1.38E-04 | 2.30E-01 | 1.25E-03 | 1.80E-03 | 4.38E-05 | 2.60E-06 | 1.62E-04 | 1.51E-03 | 1.92E-03 | 2.38E-03 | 8.31E-04 |
| **P13** | 1.13E-01 | 6.26E-03 | 5.07E-02 | 2.89E-02 | 1.01E-02 | 1.33E-02 | 5.62E-02 | 3.74E-04 | 9.05E-01 | 3.49E-02 | 3.41E-03 | 1.44E-04 | 4.95E-05 | 1.23E-03 | 5.24E-02 | 1.94E-02 | 2.31E-02 | 1.20E-03 |
| **P14** | 2.13E-01 | 1.10E-02 | 9.62E-02 | 5.47E-02 | 1.90E-02 | 2.29E-02 | 1.06E-01 | 6.97E-04 | 1.70E+00 | 6.62E-02 | 6.52E-03 | 2.69E-04 | 9.31E-05 | 2.32E-03 | 9.91E-02 | 3.64E-02 | 4.32E-02 | 1.86E-03 |
| $V_{EF}$ | 3.43E-01 | 7.49E-02 | 1.73E-01 | 9.90E-02 | 7.74E-02 | 1.34E-01 | 2.81E-01 | 7.28E-03 | 5.60E+00 | 1.15E-01 | 2.96E-02 | 1.02E-03 | 5.03E-04 | 1.17E-02 | 1.63E-01 | 7.87E-02 | 9.51E-02 | 2.30E-02 |

There is no EM for the use of the Cu-catalyzed Ullmann reaction towards **TPA-1**, because no literature is available. Therefore, no additional LCA was conducted, and only assumptions (8% lower yield compared to Pd-catalyzed BHA; impact of 3.5 mol% Cu; for **P13** and **P14**) were made.

## Table S9: **TPA-2**

**Table S9**: **Environmental Matrix (EM):** Normalized impact values $V_{EM}$(MIC,P) according to Equation 2 for all process steps and MICs involved in the lab-scale synthesis of **TPA-2** using ReCiPe 2016 for functional unit of 1 kg material.

| Category | TAP | GWP 1000 | FETP | METP | TETP | FFP | FEP | MEP | HTPc | HTPnc | IRP | LOP | SOP | ODP infinite | PMFP | HOFP | EOFP | WCP |
|---|---|---|---|---|---|---|---|---|---|---|---|---|---|---|---|---|---|---|
| **P1** | 7.77E-04 | 1.34E-03 | 1.56E-03 | 8.26E-04 | 2.17E-03 | 1.65E-03 | 5.88E-03 | 2.69E-04 | 9.14E-02 | 7.66E-04 | 1.11E-03 | 1.68E-05 | 7.15E-07 | 5.33E-05 | 4.03E-04 | 4.61E-04 | 5.68E-04 | 2.77E-04 |
| **P2** | 5.61E-05 | 3.22E-04 | 1.29E-04 | 7.37E-05 | 2.22E-04 | 4.67E-04 | 6.05E-04 | 1.04E-05 | 1.29E-02 | 6.73E-05 | 1.21E-04 | 2.17E-06 | 3.89E-07 | 1.30E-05 | 4.30E-05 | 1.15E-04 | 1.43E-04 | 4.03E-05 |
| **P3** | 2.54E-04 | 4.88E-04 | 3.80E-04 | 1.74E-04 | 5.77E-04 | 6.43E-04 | 1.35E-03 | 3.67E-04 | 2.50E-02 | 1.62E-04 | 2.04E-04 | 1.91E-04 | 3.09E-07 | 1.75E-04 | 1.31E-04 | 2.75E-04 | 3.32E-04 | 4.64E-04 |
| **P4** | 1.60E-05 | 2.34E-04 | 2.07E-05 | 1.21E-05 | 3.12E-05 | 5.16E-05 | 3.17E-04 | 1.38E-05 | 2.62E-03 | 1.10E-05 | 1.76E-05 | 4.45E-06 | 6.17E-08 | 1.07E-05 | 1.05E-05 | 3.18E-05 | 3.81E-05 | 1.58E-05 |
| **P5** | 8.37E-03 | 3.38E-02 | 1.55E-02 | 9.07E-03 | 2.47E-02 | 6.21E-02 | 7.56E-02 | 3.28E-03 | 2.19E+00 | 8.16E-03 | 1.21E-02 | 2.55E-04 | 4.05E-04 | 8.49E-03 | 5.60E-03 | 1.39E-02 | 1.74E-02 | 4.16E-03 |
| **P6** | 2.61E-03 | 1.05E-02 | 3.06E-03 | 2.01E-03 | 9.90E-03 | 1.24E-02 | 1.71E-02 | 2.89E-04 | 3.13E-01 | 1.87E-03 | 2.78E-03 | 6.08E-05 | 6.77E-06 | 2.93E-04 | 1.66E-03 | 3.40E-03 | 4.18E-03 | 1.18E-02 |
| **P7** | 7.62E-04 | 4.26E-03 | 1.32E-03 | 8.17E-04 | 2.32E-03 | 6.92E-03 | 7.12E-03 | 2.00E-04 | 1.36E-01 | 7.49E-04 | 9.81E-04 | 2.87E-05 | 3.77E-06 | 1.59E-04 | 5.30E-04 | 1.75E-03 | 2.43E-03 | 4.82E-04 |
| **P8** | 1.43E-03 | 1.95E-03 | 7.27E-04 | 4.72E-04 | 2.14E-03 | 3.59E-03 | 3.18E-03 | 4.87E-05 | 7.52E-02 | 4.38E-04 | 6.52E-04 | 1.88E-05 | 1.20E-06 | 5.01E-05 | 7.84E-04 | 8.38E-04 | 1.05E-03 | 1.99E-03 |
| **P9** | 7.09E-04 | 2.13E-03 | 1.16E-03 | 6.51E-04 | 2.11E-03 | 4.26E-03 | 4.03E-03 | 7.28E-04 | 9.87E-02 | 5.99E-04 | 6.73E-04 | 1.82E-05 | 8.92E-07 | 5.58E-05 | 4.12E-04 | 9.13E-04 | 1.15E-03 | 3.36E-04 |
| **P10** | 1.30E-04 | 2.30E-04 | 1.40E-03 | 5.86E-04 | 2.43E-03 | 2.02E-04 | 1.29E-03 | 1.01E-05 | 1.48E-02 | 5.45E-04 | 1.07E-04 | 2.94E-06 | 1.45E-06 | 8.87E-06 | 7.03E-05 | 7.90E-05 | 9.58E-05 | 2.91E-04 |
| **P11** | 3.13E-03 | 5.60E-03 | 2.93E-03 | 1.63E-03 | 5.49E-03 | 9.21E-03 | 1.06E-02 | 1.63E-04 | 2.73E-01 | 1.49E-03 | 2.13E-03 | 5.20E-05 | 3.08E-06 | 1.92E-04 | 1.79E-03 | 2.28E-03 | 2.83E-03 | 9.86E-04 |
| **P12** | 1.25E-01 | 6.80E-03 | 5.64E-02 | 3.21E-02 | 1.12E-02 | 1.43E-02 | 6.24E-02 | 4.13E-04 | 1.00E+00 | 3.88E-02 | 3.80E-03 | 1.60E-04 | 5.49E-05 | 1.37E-03 | 5.82E-02 | 2.15E-02 | 2.56E-02 | 1.27E-03 |
| **$V_{EF}$** | 1.43E-01 | 6.77E-02 | 8.46E-02 | 4.84E-02 | 6.33E-02 | 1.16E-01 | 1.89E-01 | 5.79E-03 | 4.23E+00 | 5.37E-02 | 2.47E-02 | 8.11E-04 | 4.78E-04 | 1.09E-02 | 6.96E-02 | 4.55E-02 | 5.58E-02 | 2.21E-02 |

There is no EM for the use of the Cu-catalyzed Ullmann reaction towards **TPA-2**, because no literature is available. Therefore, no additional LCA was conducted, and only assumptions (8% lower yield compared to Pd-catalyzed BHA; impact of 3.5 mol% Cu for **P12**) were made.

## Chapter S5a) Total impacts of the individual process steps of **TPAs** and the entire synthesis

### Table S10: Overview of the process radar impact values $V_{PR}$(P) **TPA**

**Table S10:** Overview of the process radar impact values $V_{PR}$ according to Equation 3 (main text) of the individual process steps and the total value according to Equation 5 (main text) for the entire synthesis of 1kg of the three **TPAs**.

| | **TPA-0** | **TPA-1** | **TPA-2** | | **TPA-0** |
|---|---|---|---|---|---|
| **catalyst** | **Pd** | **Pd** | **Pd** | | **Cu*** |
| **$V_{PR}$(P1)** | | 9.23E-02 | 1.10E-01 | | |
| **$V_{PR}$(P2)** | | 1.30E-02 | 1.54E-02 | | |
| **$V_{PR}$(P3)** | | 2.64E-02 | 3.12E-02 | | |
| **$V_{PR}$(P4)** | | 2.92E-03 | 3.46E-03 | | |
| **$V_{PR}$(P5)** | | 2.10E+00 | 2.49E+00 | | |
| **$V_{PR}$(P6)** | | 3.35E-01 | 3.96E-01 | | |
| **$V_{PR}$(P7)** | | 1.41E-01 | 1.67E-01 | | |
| **$V_{PR}$(P8)** | 1.98E-01 | 1.44E-01 | 9.45E-02 | | 1.98E-01 |
| **$V_{PR}$(P9)** | 8.14E-01 | 3.66E-01 | 1.19E-01 | | 8.14E-01 |
| **$V_{PR}$(P10)** | 2.16E-02 | 1.75E-02 | 2.23E-02 | | 4.51E-02 |
| **$V_{PR}$(P11)** | | 2.72E-01 | 3.23E-01 | | |
| **$V_{PR}$(P12)*** | | | 1.46E+00 | | |
| **$V_{PR}$(P13)*** | | 1.32E+00 | | | |
| **$V_{PR}$(P14)*** | | 2.48E+00 | | | |
| **$V_{PR}$(P15)*** | 6.23E+00 | | | | |
| **$V_{PR}$(P15)**** | | | | | 2.03E-01 |
| **$V_{total,wt}$** | 7.29E+00 | 7.31E+00 | 5.23E+00 | | 1.26E+00 |

* Pd catalyst; ** Cu catalyst

This data is plotted in Figure 5 of the main text.

## Chapter S5b) Contribution of Reagents, Energy, Solvents and Waste for **TPA** Synthesis

### Table S11: Overview of the impact values in the categories “reagents”, “solvent”, “energy”, and “waste”

**Table S11:** Overview of the impact values in the categories “reagents”, “solvent”, “energy”, and “waste” accumulated over all process steps for the respective **TPA**-derivatives and the total value $V_{total,wt}$ for the entire synthesis (compare Figure S5).

| | **TPA-0** | **TPA-1** | **TPA-2** | | **TPA-0** |
|---|---|---|---|---|---|
| **catalyst** | **Pd** | **Pd** | **Pd** | | **Cu** |
| | | | | | |
| **Reagents** | 6.90 | 5.68 | 3.49 | | 0.85 |
| **Solvent** | 0.25 | 1.00 | 1.08 | | 0.25 |
| **Energy** | 0.03 | 0.22 | 0.23 | | 0.03 |
| **Waste** | 0.11 | 0.41 | 0.44 | | 0.12 |
| $\mathbf{V_{total,wt}}$ | 7.29 | 7.31 | 5.23 | | 1.26 |

### Figure S5: Pie charts TPA Synthesis

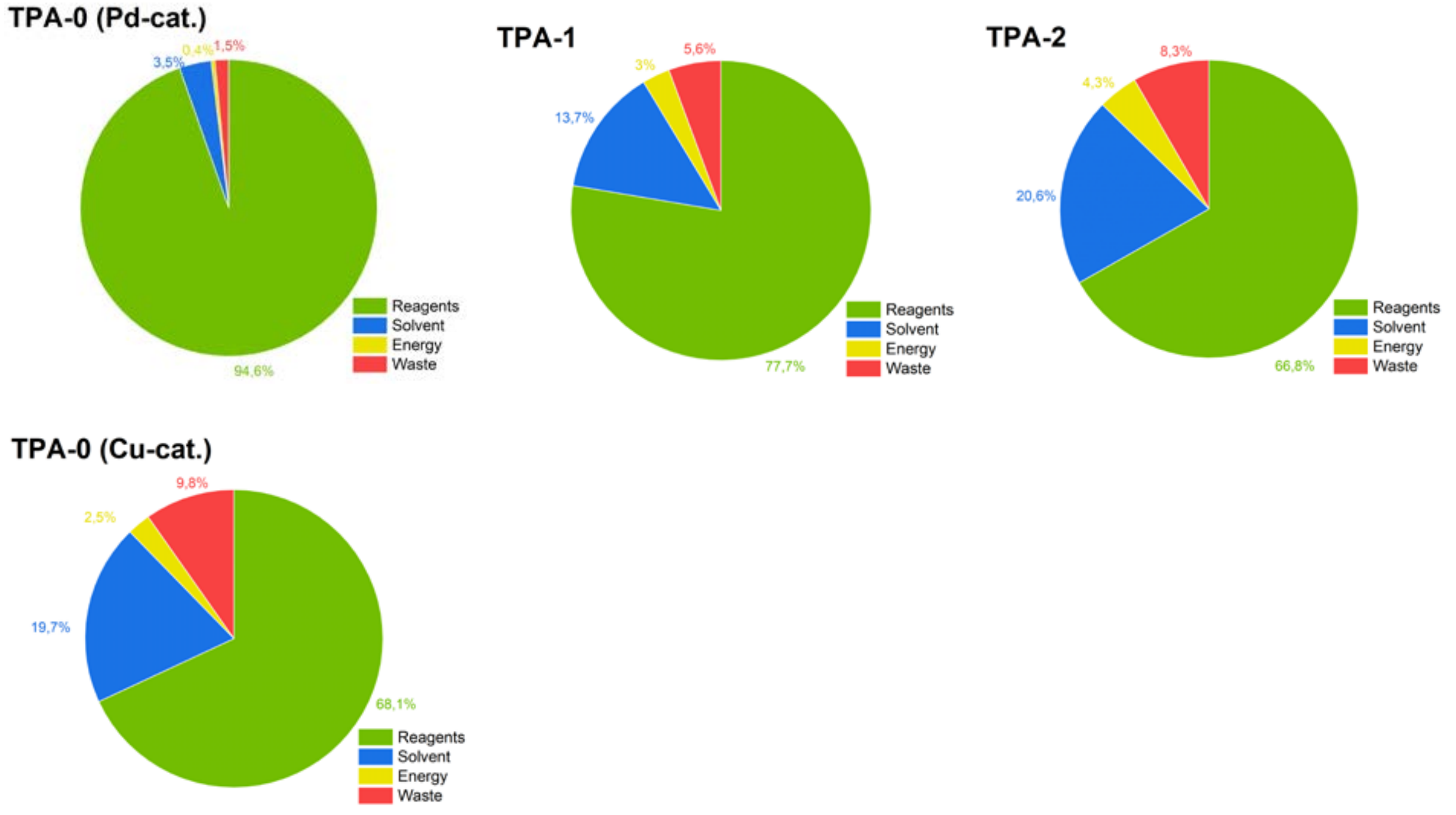


**Figure S5:** Pie-chart of the impact values of “reagents” (green), “solvents” (blue), “energy” (yellow), and “waste” (red) for the synthesis of all **TPA**-derivatives.

## Figure S6: Sensitivity analysis of energy and solvent input

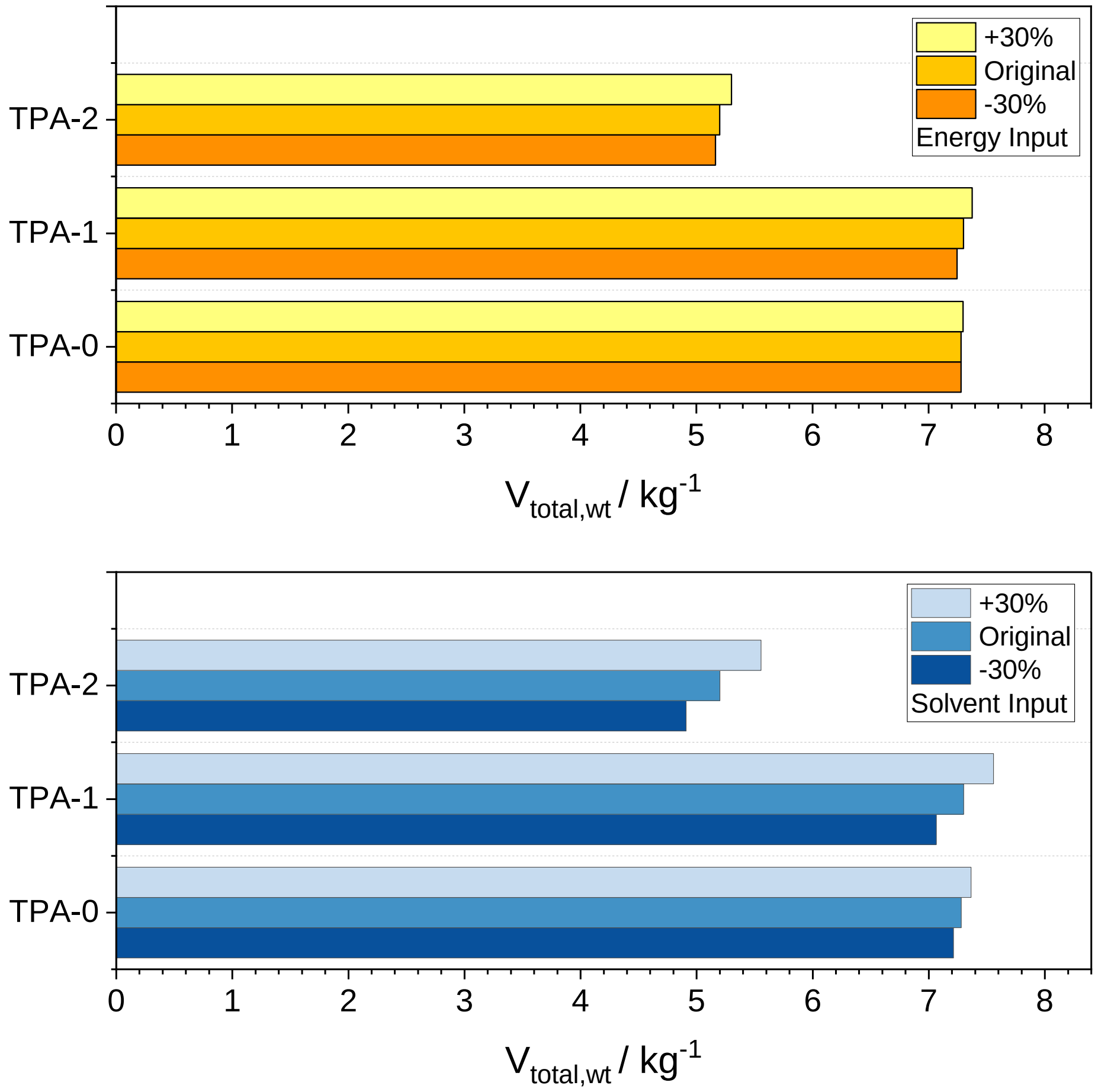


**Figure S6: Sensitivity analysis of $V_{total,wt}$ for TPA-0, TPA-1 and TPA-2.** We assumed a variation of ±30% relative to the original data. a) energy input in units of [MJ], yielding sensitivities of 0.13% (TPA-0), 0.9% (TPA-1), and 1.3% (TPA_2); b) solvent use in units of [kg]**,** yielding sensitivities of 1.0% (TPA-0), 3.3% (TPA-1), and 6.0% (TPA-2).

# Chapter S6) Building Block Approach (BBA)

## Figure S7: Illustration of the mathematical ansatz for the building-block approach (BBA)

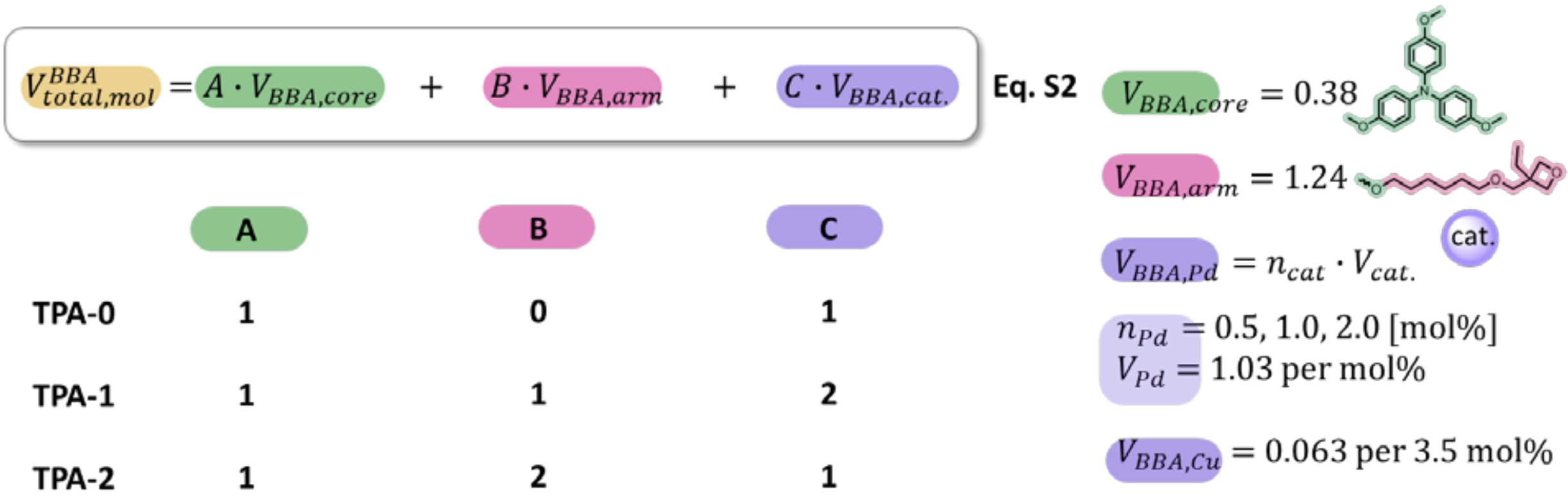


**Figure S7: Visualization of the mathematical procedure to sum up the building blocks.** The values $V_x$ of the building block approach were derived from the values calculated by ESR.

$V_{Pd}$ could directly been taken from the ESR, by simply converting the value for 1 kg palladium to 1 mol. To obtain the value $V_{BBA,Pd}$ for the actual used amount of Pd in the synthesis, $V_{Pd}$ was multiplied with the used amount of substance.

In the following, the impact of used Pd ($V_{BBA,Pd}$) could be subtracted from $V_{total,mol}$ of **TPA-0** yielding $V_{BBA,core}$. The value for $V_{BBA,core}$ can then also be applied to **TPA-1** and **TPA-2**.

The building block value of the arm $V_{BBA,arm}$ was averaged as follows: from ESR value $V_{total,mol}$ of **TPA-1** and **TPA-2** the building block values $V_{BBA,core}$ and $V_{BBA,Pd}$ were subtracted, obtaining 1.46 and 2.26, respectively. The sum 3.72 was divided by 3 (since 3 arms are involved) to obtain a average value of 1.24 for $V_{BBA,arm}$.

$V_{BBA,Cu}$ was calculated from the value of $V_{Cu}$ obtained by ESR and taking the 8% lower yield compared to the Pd route into account.

To obtain the $V_{total,mol}^{BBA}$ the corresponding building blocks to obtain **TPA-0**, **TPA-1** and **TPA-2** are summed up, Equation S2:

$$V_{total,mol}^{BBA} = A \cdot V_{BBA,core} + B \cdot V_{BBA,arm} + C \cdot V_{BBA,cat.} \qquad \textbf{Equation S2}$$

# Chapter S7) Comparison to other Metrics

## Figure S8 Alternative representation of the comparison of ESR to mass-based metrics

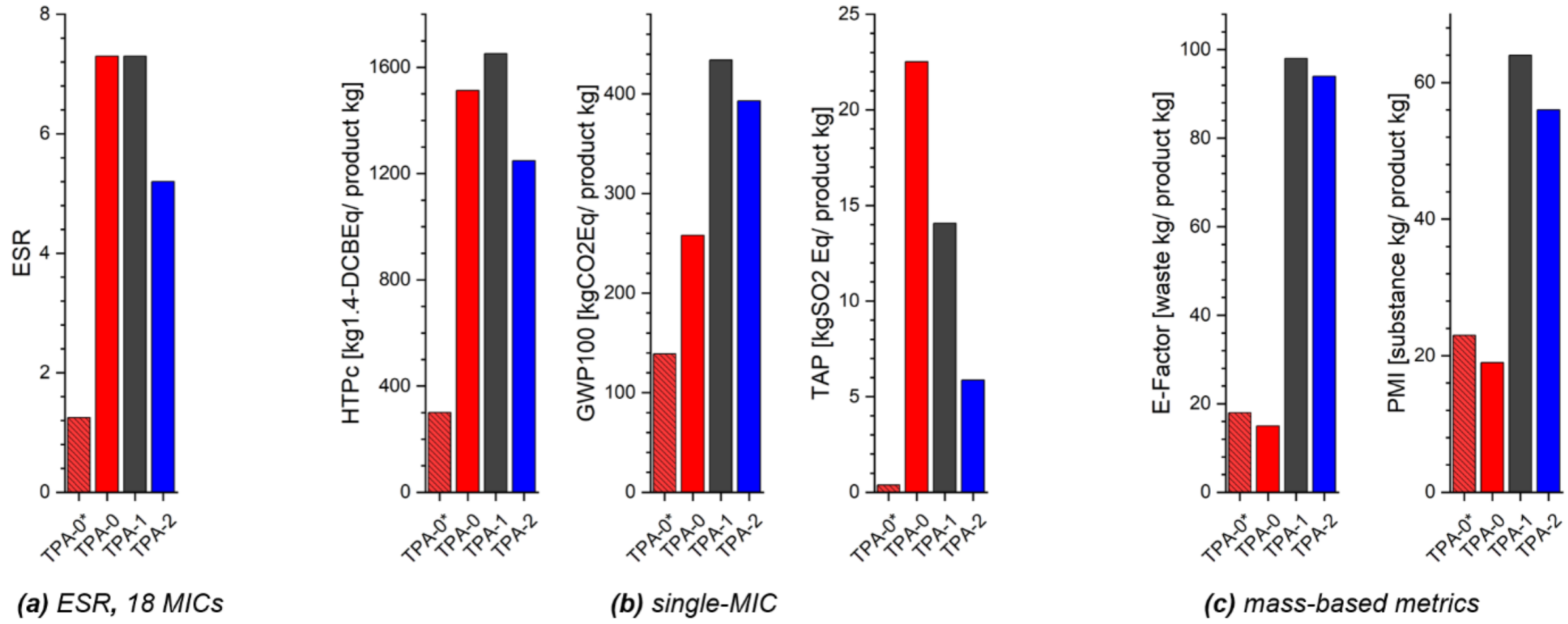


**Figure S8: Alternative representation of the comparison of ESR to mass-based metrics:** environmental impacts of the synthesis towards TPA-0 (*with Cu-catalyst), TPA-1 and TPA-2 using different approaches: (a) ESR (18 impact categories, with normalization), (b) specific single MICs "HTPc" (left, MIC 9), "GWP100" (middle, MIC 2) and "TAP" (right, MIC 1) (all without normalization), and (c) the mass-metrics "E-Factor" (left) and "PMI" (right).

# Chapter S8) **NMC** Lab-Scale Synthesis & Industrial Production

## Chapter S8a) **NMC** synthesis

### Figure S9 Flowchart **NMCxyz** laboratory scale synthesis

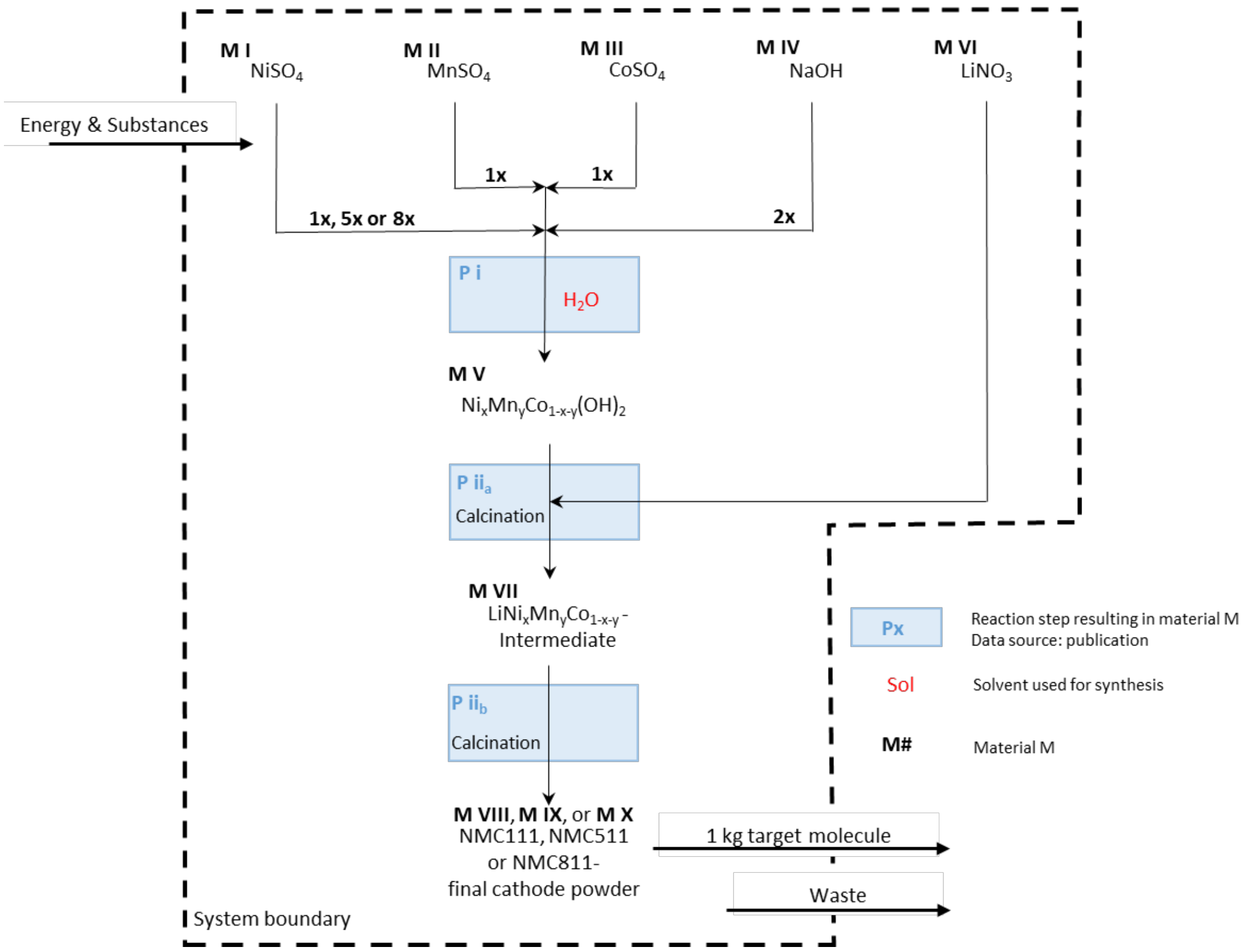


**Figure S9:** Flow chart and system boundary for the laboratory scale production of **NMC** inorganic cathode active material according to *Liu et al*.[29]. The stoichiometric ratio of the starting materials **M I**, **M II**, and **M III** is set to 1:1:1, 5:1:1 or 8:1:1. The stoichiometric ratio of **M IV**:[**M I**:**M II**:**M III**] is 2:1. The stoichiometric ratio of **M V**:**M VI** is 1:1.

## Figure S10 Flowchart **NMC811** industrial-scale production

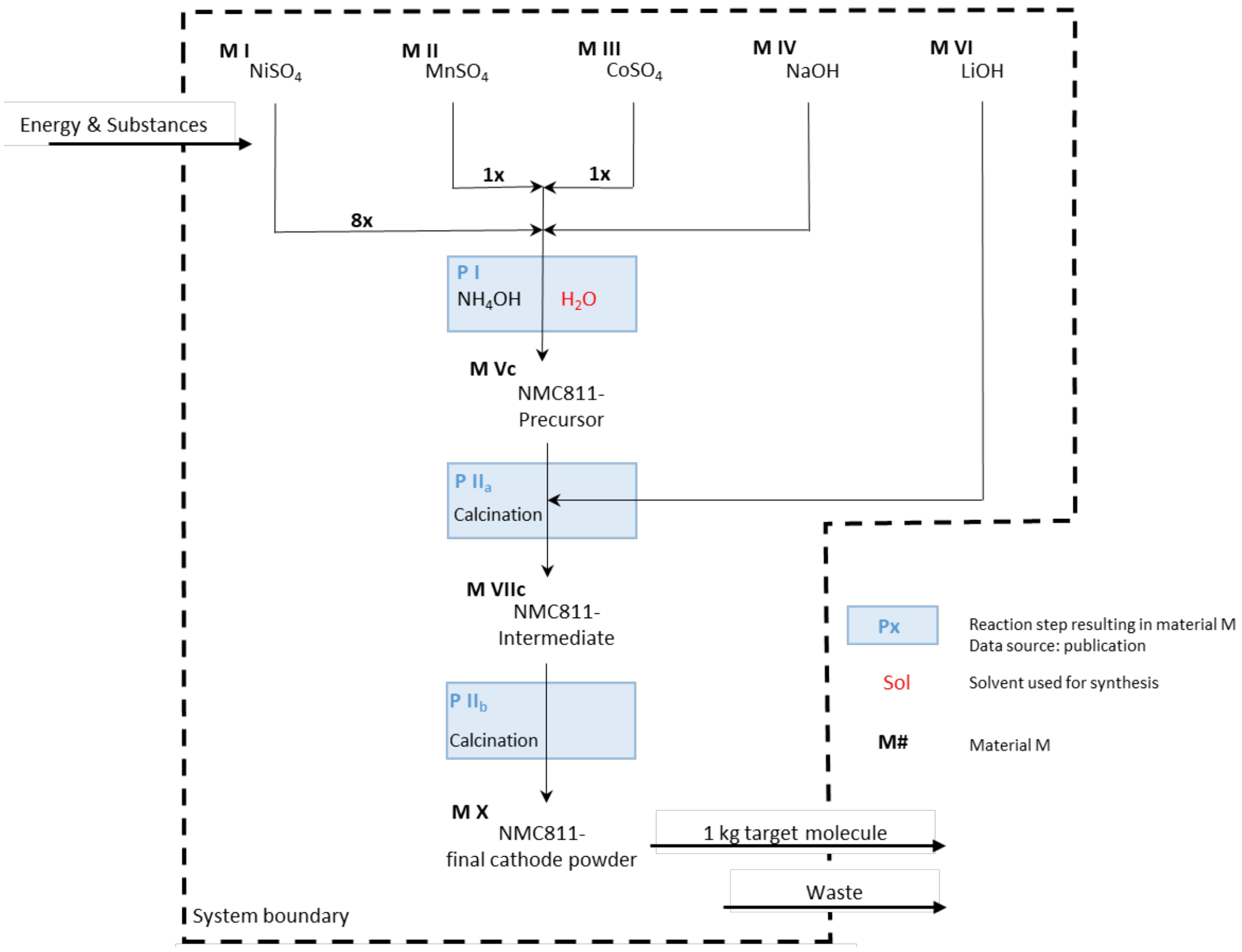


**Figure S10:** Flow chart and system boundary for the industrial production of **NMC**-based inorganic cathode active material according to E.Crenna et al. [26a]. The stoichiometric ratio of the starting materials **M I**, **M II**, and **M III** is 8:1:1. The stoichiometric ratio of **M IV**:[**M I**:**M II**:**M III**] is 1:1. $NH_4OH$ is used as a chelating agent. The stoichiometric ratio of **M V**:**M VI** varies from 1:1 to 1:1.05. The additives to promote complete calcination (oxide formation) are unknown, and no additives are considered.

## Chapter S8b) Overview process details for all four **NMC** synthesis

### Table: S12 Overview process details **NMCxyz**

**Table S12**: Process steps with detailed information for the synthesis of **NMC** from compounds that are available in the EcoInvent database. Synthesis steps are derived from the first published paper about **NMC** cathode materials for laboratory synthesis. The experimental procedure was adapted to **NMC111** ($LiNi_{0.33}Mn_{0.33}Co_{0.33}O_2$; **M VIII**), **NMC511** ($LiNi_{0.71}Mn_{0.14}Co_{0.14}O_2$; **M IX**)and **NMC811** ($LiNi_{0.8}Mn_{0.1}Co_{0.1}O_2$; **M X**). For the industry **NMC811** synthesis we refer to the literature from *Crenna et al*.[30]. Possible workup processes were dr – drying, fi – filtration, wa – washing, mixing crushing, sieving, metal detection, demagnetization, grounded and pelletized but are not considered in LCA analysis. This simplification is supported by a comparison of the self-modelled industrial-scale inventory with the corresponding ecoinvent dataset for industrial NMC811 production "NMC811 oxide production, for Li-ion battery", which showed deviations of less than 10% across all impact categories after normalization.

| **NMCxyz laboratory scale** | | | | | | | | | | |
|---|---|---|---|---|---|---|---|---|---|---|
| **P#** | Educt | Reactant | Solvent (excess factor) | Cp / J/mol·K | Reaction conditions | Product (state) | Byproduct(s) / waste | | Yield [%] | Reference |
| **Pi** | $NiSO_4$ (**M I**), $MnSO_4$ (**M II**), $CoSO_4$ (**M III**), | 2 x NaOH (**M IV**) | $H_2O$ | 75 | stirred at 60°C, 30 min;[31] drying 120°C, 8 h | $Ni_{1-x-y}Mn_xCo_y(OH)_2$ (**M Va, M Vb, M Vc**) | $Na_2SO_4$ | | 100% | Liu, Yu and Lee 1999[29] |
| **Piia** | $Ni_{1-x-y}Mn_xCo_y(OH)_2$ (**M Va, M Vb, M Vc**) | $LiNO_3$ | | | precalcined 550°C, 10 h in $O_2$ flow | **M VIIa, M VIIb, M VIIc** | $NO_2$, $H_2O$ | | 100% | Liu, Yu and Lee 1999[29] |
| **Piib** | **M VIIa, M VIIb, M VIIc** | | | | calcinated at 750°C, 24 h in $O_2$ flow | $LiNi_{1-x-y}Mn_xCo_yO_2$ (**M VIII – M X**) | | | 100% | Liu, Yu and Lee 1999[29] |
| **NMC811 industrial scale** | | | | | | | | | | |
| **P#** | Educt | Reactant | Solvent (excess factor) | Cp / J/mol·K | Reaction conditions | Product (state) | Byproduct(s) / waste | | Yield [%] | Reference |
| **PI** | $NiSO_4$ (**M I**), $MnSO_4$ (**M II**), $CoSO_4$ (**M III**), | $NH_4OH$, NaOH (**M IV**) | $H_2O$ | 75 | 50 °C | $Ni_{0.8}Mn_{0.1}Co_{0.1}(OH)_2$ (**M Vc**) | ½ $Na_2SO_4$ ½ $(NH_4)_2SO_4$ | | 100% | Crenna et al. 2021[30] |
| **PIIa** | $Ni_{0.8}Mn_{0.1}Co_{0.1}(OH)_2$ (**M Vc**) | LiOH (**M VI**) | | | Calcination 1000 °C, 12 h | NMC811 intermediate (**M VIIc**) | | | 100% | Crenna et al. 2021[30] |
| **PIIb** | NMC811 intermediate (**M VIIc**) | additives | | | Mixing, 1000 °C, 12 h | $LiNi_{0.8}Mn_{0.1}Co_{0.1}O_2$ (**M X - NMC811**) | | | 100% | Crenna et al. 2021[30] |

# Chapter S8c) **NMC** Life Cycle Inventory

## Contributions of each Process Step for **NMC111** lab-synthesis

### Table S13a: LCI of Pi - Synthesis of $Ni_{0.1}Mn_{0.1}Co_{0.1}(OH)_2$ (M Va)

| **Inputs** | **Dataset** | **Amount** | **$x_i$** | **Unit** |
|---|---|---|---|---|
| Nickel sulfate | market for nickel sulfate | 0.563 | 0.563 | kg |
| Cobalt sulfate | market for cobalt sulfate | 0.564 | 0.564 | kg |
| Manganese sulfate | market for manganese sulfate | 0.549 | 0.549 | kg |
| Sodium hydroxide, without water, in 50% solution state | market for sodium hydroxide, without water, in 50% solution state | 0.869 | 0.830 | kg |
| Water, deionized | market for water, deionized | 1.00 | 0.955 | kg |
| Electricity, low voltage | market for electricity | 3.24 | 3.07 | MJ |
| **Outputs** | **Dataset** | **Amount** | **$x_i$** | **Unit** |
| $Ni_{0.1}Mn_{0.1}Co_{0.1}(OH)_2$ (M Va) | Pi: Synthesis of $Ni_{0.1}Mn_{0.1}Co_{0.1}(OH)_2$ (M Va) | 1.00 | 0.955 | kg |
| Waste | Treatment of spent solvent mixture, hazardous waste incineration | 1.54 | 1.47 | kg |

### Table S13b: LCI of Pii - Synthesis of $LiNi_{0.1}Mn_{0.1}Co_{0.1}O_2$ (M VIII – **NMC111**)

| **Inputs** | **Dataset** | **Amount** | **$x_i$** | **Unit** |
|---|---|---|---|---|
| *$Ni_{0.1}Mn_{0.1}Co_{0.1}(OH)_2$ (M Va)* | *Pi: Synthesis of $Ni_{0.1}Mn_{0.1}Co_{0.1}(OH)_2$ (M Va)* | *0.955* | *0.955* | *kg* |
| Lithium sulfate | market for lithium sulfate | 0.714 | 0.714 | kg |
| Electricity, low voltage | market for electricity | 184 | 184 | MJ |
| **Outputs** | **Dataset** | **Amount** | **$x_i$** | **Unit** |
| $LiNi_{0.1}Mn_{0.1}Co_{0.1}O_2$ (M VIII – NMC111) | Pii: Synthesis of $LiNi_{0.1}Mn_{0.1}Co_{0.1}O_2$ (M VIII – NMC111) | 1.00 | 1.00 | kg |
| Waste | Treatment of spent solvent mixture, hazardous waste incineration | 0.662 | 0.662 | kg |

Contributions of each Process Step for **NMC511** lab-synthesis

Table S13c: LCI of Pi - Synthesis of $Ni_{0.72}Mn_{0.14}Co_{0.14}(OH)_2$ (M Vb)

| **Inputs** | **Dataset** | **Amount** | **$x_i$** | **Unit** |
|---|---|---|---|---|
| Nickel sulfate | market for nickel sulfate | 1.20 | 1.20 | kg |
| Cobalt sulfate | market for cobalt sulfate | 0.237 | 0.237 | kg |
| Manganese sulfate | market for manganese sulfate | 0.231 | 0.231 | kg |
| Sodium hydroxide, without water, in 50% solution state | market for sodium hydroxide, without water, in 50% solution state | 0.869 | 0.829 | kg |
| Water, deionized | market for water, deionized | 1.00 | 0.955 | kg |
| Electricity, low voltage | market for electricity | 3.22 | 3.07 | MJ |
| **Outputs** | **Dataset** | **Amount** | **$x_i$** | **Unit** |
| $Ni_{0.72}Mn_{0.14}Co_{0.14}(OH)_2$ (M Vb) | *Pi: Synthesis of $Ni_{0.72}Mn_{0.14}Co_{0.14}(OH)_2$ (M Vb)* | 1.00 | 0.955 | kg |
| Waste | Treatment of spent solvent mixture, hazardous waste incineration | 1.54 | 1.47 | kg |

Table S13d: LCI of Pii - Synthesis of $LiNi_{0.72}Mn_{0.14}Co_{0.14}O_2$ (M IX – **NMC511**)

| **Inputs** | **Dataset** | **Amount** | **$x_i$** | **Unit** |
|---|---|---|---|---|
| *$Ni_{0.72}Mn_{0.14}Co_{0.14}(OH)_2$ (M Vb)* | *Pi: Synthesis of $Ni_{0.72}Mn_{0.14}Co_{0.14}(OH)_2$ (M Vb)* | *0.955* | *0.955* | *kg* |
| Lithium sulfate | market for lithium sulfate | 0.714 | 0.714 | kg |
| Electricity, low voltage | market for electricity | 184 | 184 | MJ |
| **Outputs** | **Dataset** | **Amount** | **$x_i$** | **Unit** |
| *$LiNi_{0.72}Mn_{0.14}Co_{0.14}O_2$ (M IX – NMC511)* | Pii: Synthesis of *$LiNi_{0.72}Mn_{0.14}Co_{0.14}O_2$ (M IX – NMC511)* | 1.00 | 1.00 | kg |
| Waste | Treatment of spent solvent mixture, hazardous waste incineration | 0.662 | 0.662 | kg |

## Contributions of each Process Step for **NMC811** lab-synthesis

### Table S13e: LCI of Pi - Synthesis of $Ni_{0.8}Mn_{0.1}Co_{0.1}(OH)_2$ (M Vc)

| **Inputs** | **Dataset** | **Amount** | **$x_i$** | **Unit** |
|---|---|---|---|---|
| Nickel sulfate | market for nickel sulfate | 1.28 | 1.28 | kg |
| Cobalt sulfate | market for cobalt sulfate | 0.160 | 0.160 | kg |
| Manganese sulfate | market for manganese sulfate | 0.156 | 0.156 | kg |
| Sodium hydroxide, without water, in 50% solution state | market for sodium hydroxide, without water, in 50% solution state | 0.869 | 0.830 | kg |
| Water, deionized | market for water, deionized | 1.00 | 0.955 | kg |
| Electricity, low voltage | market for electricity | 3.22 | 3.07 | MJ |
| **Outputs** | **Dataset** | **Amount** | **$x_i$** | **Unit** |
| $Ni_{0.8}Mn_{0.1}Co_{0.1}(OH)_2$ (M Vc) | *Pi: Synthesis of $Ni_{0.8}Mn_{0.1}Co_{0.1}(OH)_2$ (M Vc)* | 1.00 | 0.955 | kg |
| Waste | Treatment of spent solvent mixture, hazardous waste incineration | 1.54 | 1.47 | kg |

### Table S13f: LCI of Pii - Synthesis of $LiNi_{0.8}Mn_{0.1}Co_{0.1}O_2$ (M X – **NMC811**)

| **Inputs** | **Dataset** | **Amount** | **$x_i$** | **Unit** |
|---|---|---|---|---|
| *$Ni_{0.8}Mn_{0.1}Co_{0.1}(OH)_2$ (M Vc)* | *Pi: Synthesis of $Ni_{0.8}Mn_{0.1}Co_{0.1}(OH)_2$ (M Vc)* | *0.955* | *0.955* | *kg* |
| Lithium sulfate | market for lithium sulfate | 0.714 | 0.714 | kg |
| Electricity, low voltage | market for electricity | 184 | 184 | MJ |
| **Outputs** | **Dataset** | **Amount** | **$x_i$** | **Unit** |
| $LiNi_{0.8}Mn_{0.1}Co_{0.1}O_2$ (M X – NMC811) | *Pii: Synthesis of $LiNi_{0.8}Mn_{0.1}Co_{0.1}O_2$ (M X – NMC811)* | 1.00 | 1.00 | kg |
| Waste | Treatment of spent solvent mixture, hazardous waste incineration | 0.662 | 0.662 | kg |

Table S14: Specific data for the synthesis of LiNixMnyCO1-x-y (**NMC111**, **NMC511**, and **NMC811**) from the EcoInvent database.

| Category | TAP | GWP 1000 | FETP | METP | TETP | FFP | FEP | MEP | HTPc | HTPnc | IRP | LOP | SOP | ODP infinite | PMFP | HOFP | EOFP | WCP |
|---|---|---|---|---|---|---|---|---|---|---|---|---|---|---|---|---|---|---|
| **Unit** | kg SO2-Eq | kg CO2-Eq | kg 1.4-DCB-Eq | kg 1.4-DCB-Eq | kg 1.4-DCB-Eq | kg oil-Eq | kg P-Eq | kg N-Eq | kg 1.4-DCB-Eq | kg 1.4-DCB-Eq | kBq Co-60-Eq | m2*a crop-Eq | kg Cu-Eq | kg CFC-11-Eq | kg PM2.5-Eq | kg NOx-Eq | kg NOx-Eq | m3 |
| Cobalt sulfate | | | | | | | | | | | | | | | | | | |
| Lithium nitrate | | | | | | | | | | | | | | | | | | |
| Manganese sulfate | | | | | | | | | | | | | | | | | | |
| Nickel Sulfate | | | | | | | | | | | | | | | | | | |
| Sodium hydroxide | | | | | | | | | | | | | | | | | | |
| Water, deionized | | | | | | | | | | | | | | | | | | |
| Electricity | | | | | | | | | | | | | | | | | | |
| Waste | | | | | | | | | | | | | | | | | | |

Raw data cannot be published here. Licence from EcoInvent required.

# Chapter S8d) Environmental Matrix **EM** (process steps vs. MICs) for **NMC**

## Table S15: Normalized impact values $V_{EM}$(MIC,P) **NMC**

**Table S15:** Normalized impact values $V_{EM}$(MIC,P) according to Equation 2 (main text) for all process steps and MICs involved in the lab-scale synthesis of **NMCxyz** using ReCiPe 2016 for functional unit of 1 kg material. The stoichiometric ratio of the starting materials is set to 1:1:1, 5:1:1 or 8:1:1, respectively.

| Category | TAP | GWP 1000 | FETP | METP | TETP | FFP | FEP | MEP | HTPc | HTPnc | IRP | LOP | SOP | ODP infinite | PMFP | HOFP | EOFP | WCP |
|---|---|---|---|---|---|---|---|---|---|---|---|---|---|---|---|---|---|---|
| **NMC111 laboratory scale** | | | | | | | | | | | | | | | | | | |
| **Pi** | 4.23E-03 | 3.79E-03 | 1.22E-02 | 6.74E-03 | 5.21E-02 | 6.39E-03 | 1.51E-02 | 1.05E-03 | 4.52E-01 | 6.97E-03 | 1.44E-02 | 9.66E-05 | 5.34E-05 | 3.50E-04 | 2.41E-03 | 2.46E-03 | 2.99E-03 | 1.36E-02 |
| **Pii** | 1.52E-03 | 4.34E-03 | 1.10E-02 | 5,67E-03 | 9.96E-03 | 6.47E-03 | 5.22E-02 | 8.29E-04 | 5.44E-01 | 5.29E-03 | 9.77E-03 | 9.55E-05 | 6.10E-05 | 2.86E-04 | 8.79E-04 | 1.75E-03 | 2.09E-03 | 6.06E-04 |
| **$V_{EF}$** | 5.75E-03 | 8.12E-03 | 2.32E-02 | 1.24E-02 | 6.21E-02 | 1.29E-02 | 6.73E-02 | 1.88E-03 | 9.96E-01 | 1.23E-02 | 2.42E-02 | 1.92E-04 | 1.14E-04 | 6.36E-04 | 3.29E-03 | 4.21E-03 | 5.08E-03 | 1.42E-02 |
| **NMC511 laboratory scale** | | | | | | | | | | | | | | | | | | |
| **Pi** | 3.89E-03 | 2.76E-03 | 9.64E-03 | 5.24E-03 | 4.24E-02 | 4.63E-03 | 1.13E-02 | 7.63E-04 | 3.09E-01 | 5.39E-03 | 1.07E-02 | 7.13E-05 | 4.02E-05 | 2.61E-04 | 2.12E-03 | 1.84E-03 | 2.23E-03 | 1.01E-02 |
| **Pii** | 1.52E-03 | 4.34E-03 | 1.10E-02 | 5.67E-03 | 9.96E-03 | 6.47E-03 | 5.22E-02 | 8.29E-04 | 5.44E-01 | 5.29E-03 | 9.77E-03 | 9.55E-05 | 6.10E-05 | 2.86E-04 | 8.79E-04 | 1.75E-03 | 2.09E-03 | 6.06E-04 |
| **$V_{EF}$** | 5.41E-03 | 7.09E-03 | 2.07E-02 | 1.09E-02 | 5.24E-02 | 1.11E-02 | 6.35E-02 | 1.59E-03 | 8.53E-01 | 1.07E-02 | 2.04E-02 | 1.67E-04 | 1.01E-04 | 5.46E-04 | 3.00E-03 | 3.59E-03 | 4.32E-03 | 1.07E-02 |
| **NMC811 laboratory scale** | | | | | | | | | | | | | | | | | | |
| **Pi** | 3.67E-03 | 2.47E-03 | 8.74E-03 | 4.73E-03 | 3.88E-02 | 4.10E-03 | 1.02E-02 | 6.74E-04 | 2.68E-01 | 4.86E-03 | 9.51E-03 | 6.35E-05 | 3.59E-05 | 2.33E-04 | 1.98E-03 | 1.64E-03 | 2.00E-03 | 9.02E-03 |
| **Pii** | 1.52E-03 | 4.34E-03 | 1.10E-02 | 5.67E-03 | 9.96E-03 | 6.47E-03 | 5.22E-02 | 8.29E-04 | 5.44E-01 | 5.29E-03 | 9.77E-03 | 9.55E-05 | 6.10E-05 | 2.86E-04 | 8.79E-04 | 1.75E-03 | 2.09E-03 | 6.06E-04 |
| **$V_{EF}$** | 5.19E-03 | 6.80E-03 | 1.98E-02 | 1.04E-02 | 4.87E-02 | 1.06E-02 | 6.24E-02 | 1.50E-03 | 8.12E-01 | 1.01E-02 | 1.93E-02 | 1.59E-04 | 9.68E-05 | 5.19E-04 | 2.86E-03 | 3.39E-03 | 4.09E-03 | 9.63E-03 |
| **NMC811 industrial scale** | | | | | | | | | | | | | | | | | | |
| **PI** | 3.83E-03 | 2.36E-03 | 9.00E-03 | 4.88E-03 | 4.05E-02 | 4.93E-03 | 9.23E-03 | 6.89E-04 | 2.78E-01 | 5.02E-03 | 9.82E-03 | 6.51E-05 | 3.77E-05 | 2.31E-04 | 2.07E-03 | 1.75E-03 | 2.02E-03 | 9.47E-03 |
| **PII** | 6.22E-04 | 1.13E-03 | 1.61E-03 | 1.13E-03 | 2.32E-03 | 1.77E-03 | 1.06E-02 | 6.09E-04 | 1.22E-01 | 1.09E-03 | 1.78E-03 | 2.56E-05 | 8.94E-05 | 6.32E-05 | 3.79E-04 | 7.56E-04 | 9.00E-04 | 2.04E-04 |
| **$V_{EF}$** | 4.45E-03 | 3.50E-03 | 1.06E-02 | 6.01E-03 | 4.29E-02 | 6.70E-03 | 1.98E-02 | 1.30E-03 | 3.99E-01 | 6.10E-03 | 1.16E-02 | 9.07E-05 | 1.27E-04 | 2.94E-04 | 2.45E-03 | 2.50E-03 | 9.00E-04 | 9.67E-03 |

## Chapter S8e) Total impacts of the individual process steps of **NMCxyz** and the entire synthesis

### Table S16: Overview of the process radar **NMC**

**Table S16:** Overview of the process radar impact values $V_{PR}$ according to Equation 3 (main text) of the individual process steps and the total value for the entire synthesis of both **TPA**.

| | **NMC111** | **NMC511** | **NMC811** | **NMC811** |
|---|---|---|---|---|
| | **Lab-scale** | **Lab-scale** | **Lab-scale** | **Industrial scale** |
| **$V_{PR}$( Pi )** | 0.60 | 0.42 | 0.37 | |
| **$V_{PR}$( Pii )** | 0.66 | 0.66 | 0.66 | |
| **$V_{PR}$( PI )** | | | | 0.38 |
| **$V_{PR}$( PII )** | | | | 0.15 |
| **$V_{total,wt}$** | 1.3 | 1.1 | 1.0 | 0.53 |

## Figure S11: Comparison Environmental Fingerprints **EF** for **TPAs** & **NMCs**

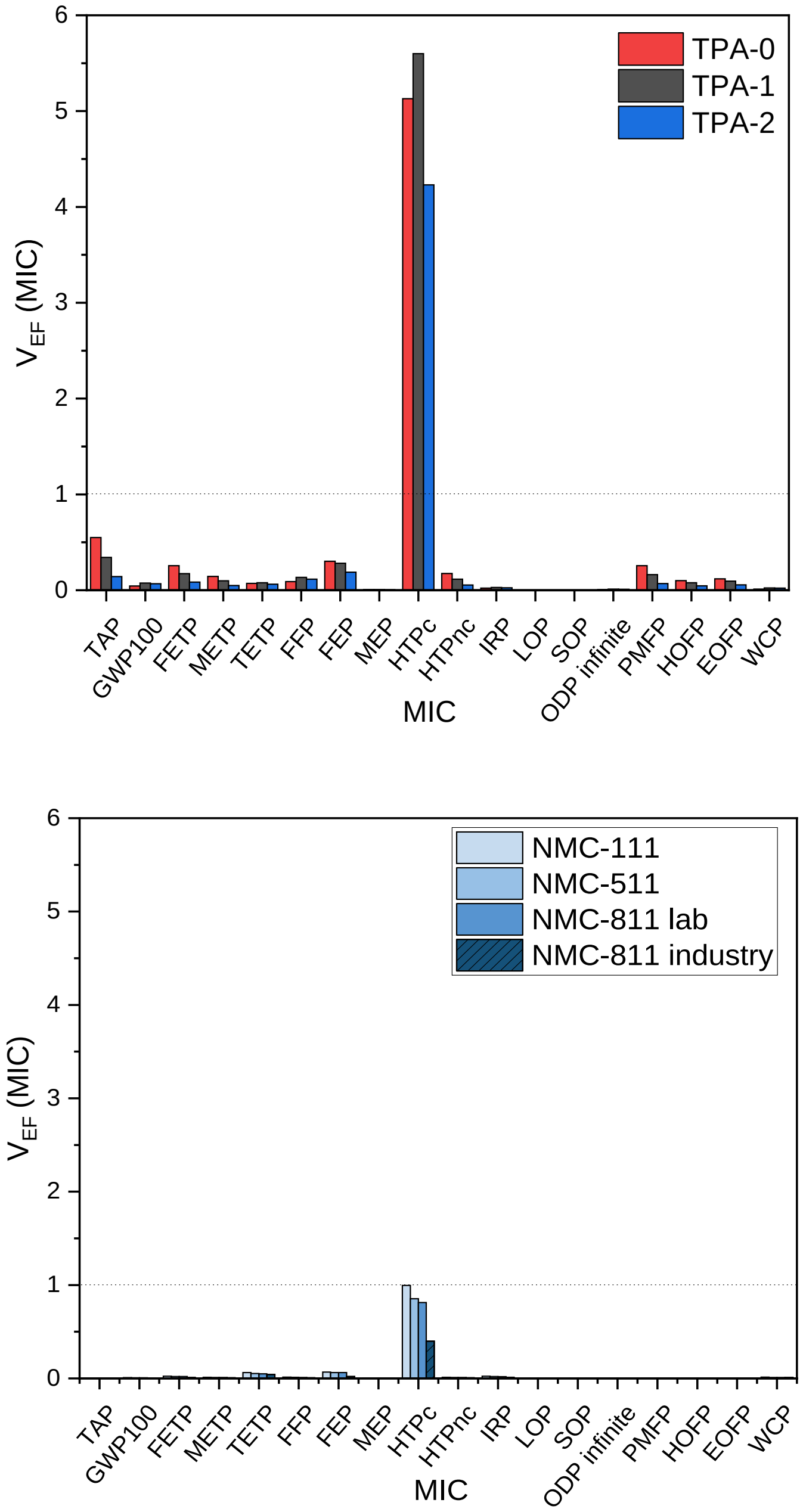


**Figure S11:** Environmental fingerprint bar charts for the normalized environmental impacts of all process steps per MIC for **TPAs** (top; **TPA-0** (red), **TPA-1** (black) and **TPA-2** (blue)) and **NMCs** (bottom; **NMC511** (lab-scale, light blue), **NMC511** (lab-scale, medium blue), **NMC811** (lab-scale, blue), and **NMC811** (industrial scale, dark blue, dashed)). Data were taken from Table S7-S9 and Table S15.

## Figure S12: Process Radar (PR) for industrial and laboratory scale **NMCxyz** synthesis

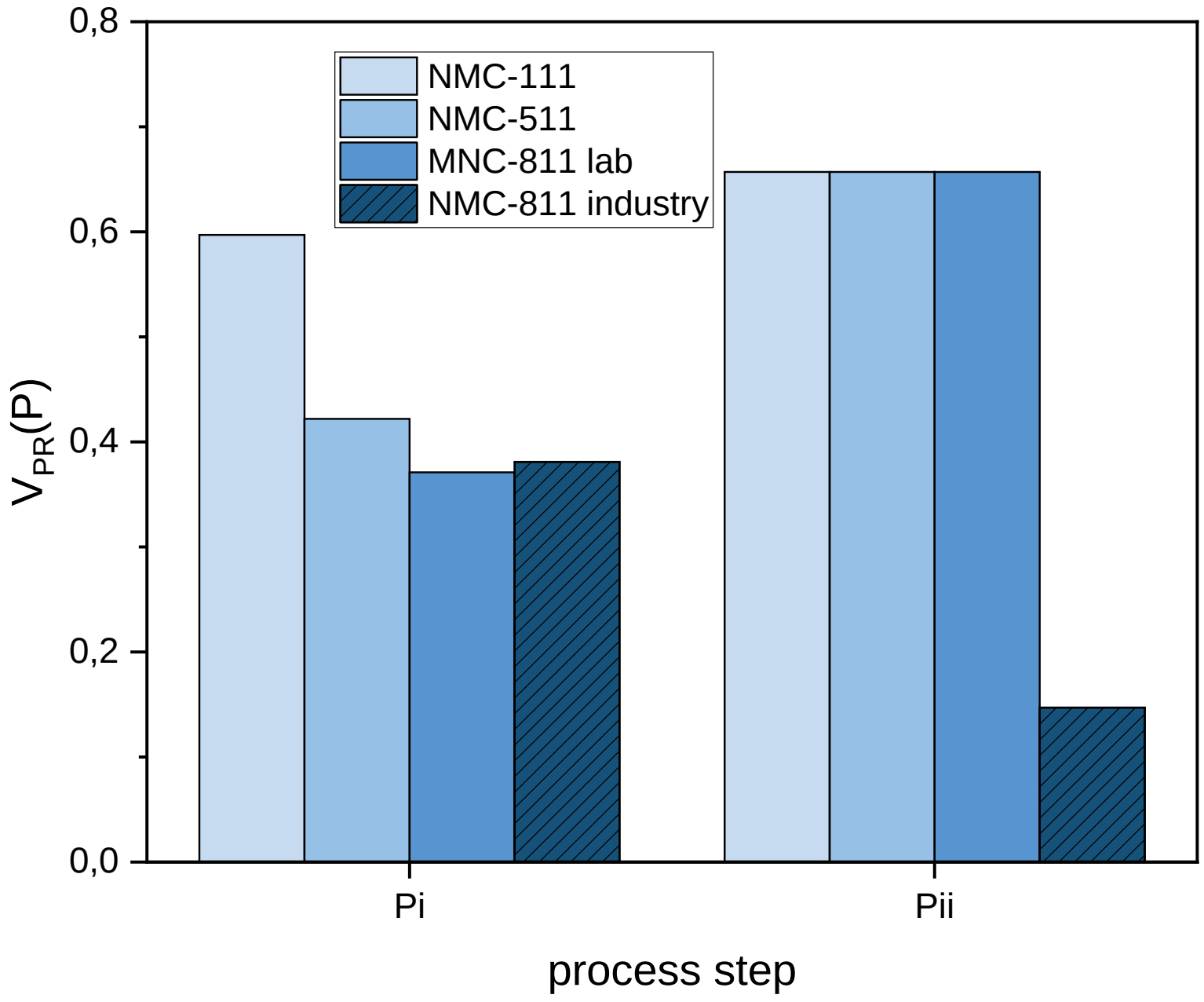


**Figure S12:** Process radar $\mathbf{V_{PR}(P)}$ based on the ESR model for the production of **NMC111** (lab-scale, light blue), **NMC511** (lab-scale, medium blue), **NMC811** (lab-scale, blue), and **NMC811** (industrial scale, dark blue, dashed) for 1 kg target material. Data taken from Table S16.

## Chapter S8f) Contribution of Reagents, Energy, Solvents and Waste for **NMC** Synthesis

### Table S17: Overview of the impact values in the categories “reagents”, “solvent”, “energy”, and “waste”

**Table S17:** Overview of the impact values in the categories “reagents”, “solvent”, “energy”, and “waste” accumulated over all process steps for the respective **NMC**-derivatives and the total value $V_{total,wt}$ for the entire synthesis (compare Figure S12).

| | **NMC111** | **NMC511** | **NMC811** | … | **NMC811** |
|---|---|---|---|---|---|
| **synthesized scale** | **Lab.** | **Lab.** | **Lab.** | | **Ind.** |
| | | | | | |
| **Reagents** | 0.66 | 0.49 | 0.43 | | 0.44 |
| **Solvent** | 0.0 | 0.0 | 0.0 | | 0.0 |
| **Energy** | 0.59 | 0.59 | 0.59 | | 9.12E-02 |
| **Waste** | 9.23E-03 | 9.23E-03 | 9.23E-03 | | 0.00 |
| **$V_{total,wt}$** | 1.25 | 1.08 | 1.03 | | 0.53 |

### Figure S13: Pie charts NMC synthesis/production

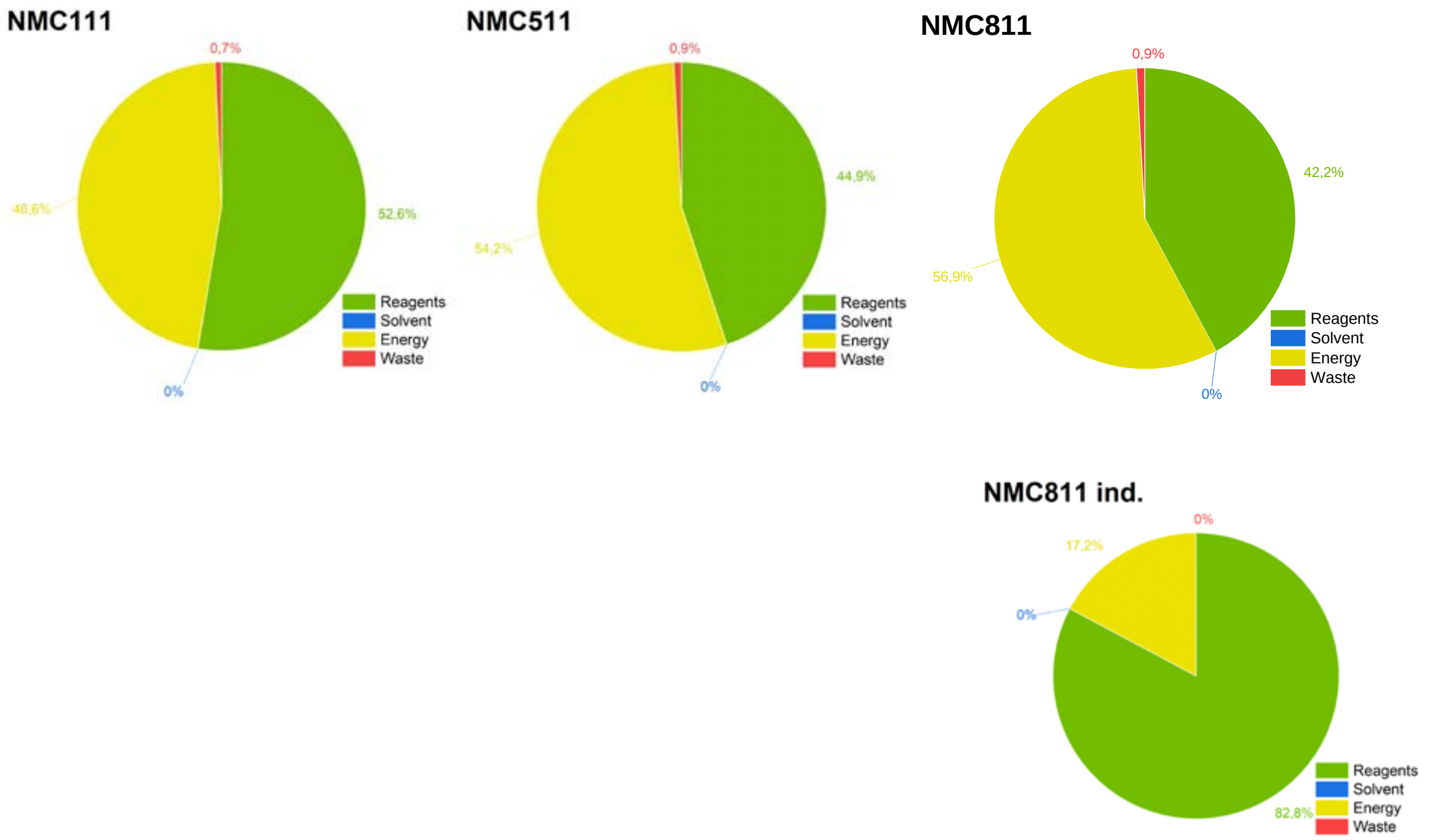


**Figure S13:** Pie-chart of the impact values of “reagents” (green), “solvents” (blue), “energy” (yellow), and “waste” (red) for the synthesis of all **NMC**-derivatives synthesized in laboratory scale and industrial scale.

## Figure S14: Sensitivity analysis of energy and solvent input

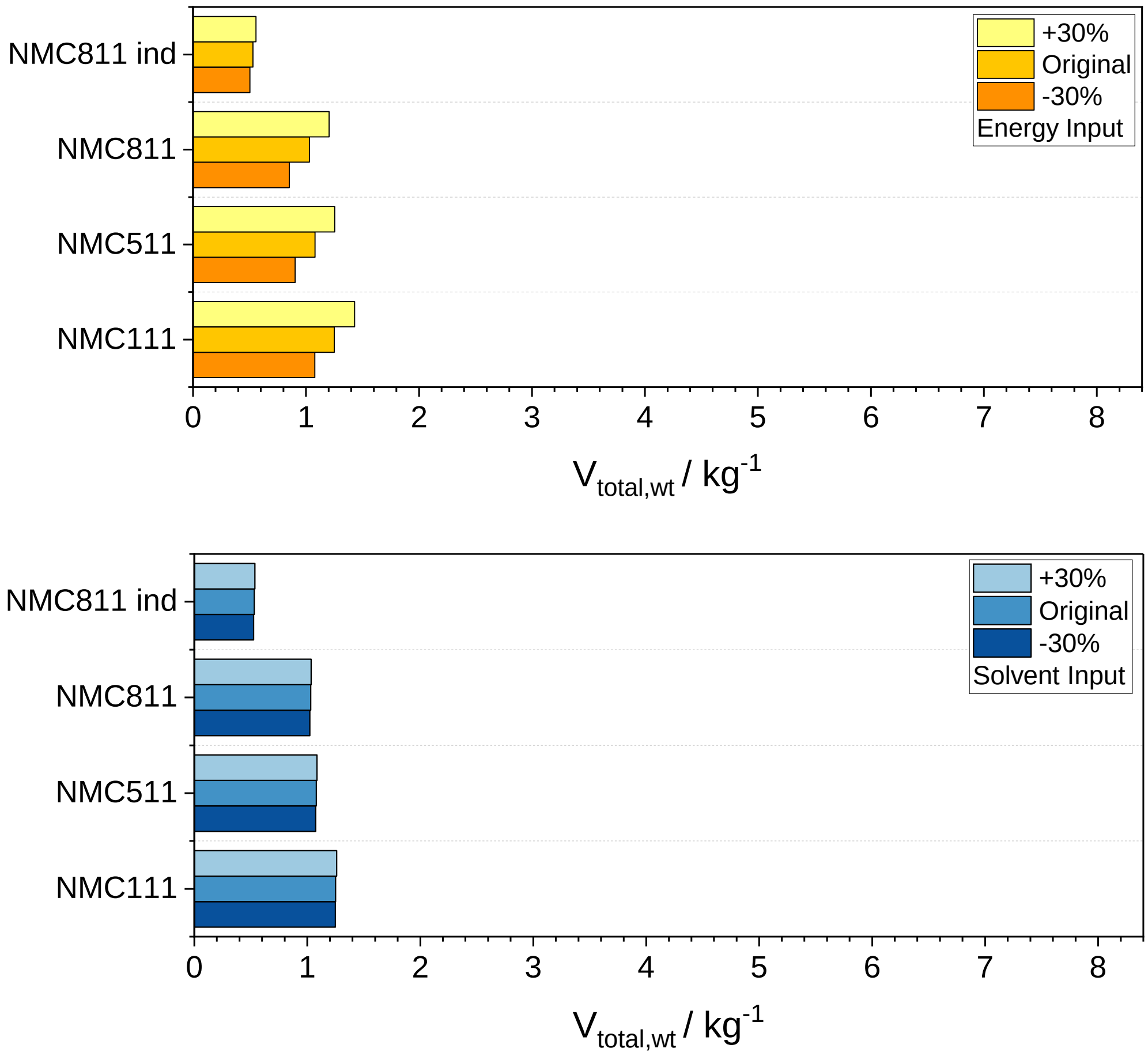


**Figure S14: Sensitivity analysis of $V_{total,wt}$ for NMC111, NMC511and NMC811 on lab. scale NMC811 on industry scale.** We assumed a variation of ±30% relative to the original data. a) energy input in units of [MJ], yielding sensitivities of 13% (NMC111), 15% (NMC511), 16% (NMC811) and 5.5% (NMC811 ind.); b) solvent use in units of [kg]**,** yielding sensitivities of 0.7% (NMC111), 0.6% (NMC511), 0.6 (NMC811) and 1.2% (NMC811 ind.).

# Chapter S9) Estimation of world-wide lithium-ion battery (LIB) production - validity and applicability of the ESR formalism

As a final validation of the ESR formalism and its practical applicability, we apply the results obtained from the ESR analysis of **NMC811** to estimate the environmental impact of global lithium-ion battery (LIB) production.

Using 2024 as the reference year,[32,33] we find that the production of LIB active materials accounts for approximately 1% of total global emissions, as illustrated in Figure S15.

- In 2024, the global need for LIB cathode materials was reported to be 2.8 E+9 kg.[32,33] Assuming that the market of LIB can be approximated/represented by NMC811 and using $V_{total,wt} = 0.5\ kg^{-1}$, we obtain emissions of **1.4 E+9 [dimensionless]** for the industrial production of LIB.

- On the other hand, each human being emits $V_{total,wt} = 18\ human^{-1}$ (see Equation 3, main text), and the global population in 2024 amounted to 8.2 billion (8.2 E+9 humans), resulting in world-wide total emissions of **147.6 E+9 [dimensionless].** Here we assumed that between 2010 (the year the normalization factors were published) and 2024, total emissions increased similar to the global population.

$$\mathrm{m(world\ demand\ of\ cathode\ material)} = 2.8 \cdot 10^{9}\ \mathrm{kg}$$

$$\mathrm{n(world\ population, 2024)} = 8.2 \cdot 10^{9}\ \mathrm{human}$$

$$V_{\mathrm{total,wt}}(\mathrm{NMC, 811\ ind.}) = 0.5\ \mathrm{kg}^{-1}$$

$$V_{\mathrm{total}}(\mathrm{per\ person}) = 18\ \mathrm{human}^{-1}$$

$$\begin{aligned}\mathrm{emission(world\ cathode\ production)} &= V_{\mathrm{total,wt}}(\mathrm{NMC, 811\ ind.}) \cdot \mathrm{m(world\ demand\ of\ cathode\ material)} \\ &= 0.5\ \mathrm{kg}^{-1} \cdot 2.8 \cdot 10^{9}\ \mathrm{kg} \\ &= 1.4 \cdot 10^{9}\end{aligned}$$

$$\begin{aligned}\mathrm{emission(world\ population)} &= V_{\mathrm{total}}(\mathrm{per\ person}) \cdot \mathrm{n(world\ population, 2024)} \\ &= 18\ \mathrm{human}^{-1} \cdot 8.2 \cdot 10^{9}\ \mathrm{human} \\ &= 147.6 \cdot 10^{9}\end{aligned}$$

$$\begin{aligned}\mathrm{contribution\ of\ cathode\ emission\ to\ world\ emisson} &= \frac{\mathrm{emission(world\ cathode\ production)}}{\mathrm{emission(world\ population)}} \\ &= \frac{147.6 \cdot 10^{9}}{1.4 \cdot 10^{9}} \\ &= \sim 1\%\end{aligned}$$

**Figure S15:** Illustration of the calculation of the contribution of LIB cathode material to the total world emissions.

# Chapter S10) References in Supporting Information


[1] M. A. J. Huijbregts, Z. J. N. Steinmann, P. M. F. Elshout et al. "ReCiPe2016: a harmonised life cycle impact assessment method at midpoint and endpoint level". *The International Journal of Life Cycle Assessment,* 22 (2017): 138-147 https://doi.org/10.1007/s11367-016-1246-y

[2] G. Wernet, S. Papadokonstantakis, S. Hellweg and K. Hungerbühler. "Bridging data gaps in environmental assessments: Modeling impacts of fine and basic chemical production". *Green Chemistry,* 11 (2009): 1826-1831 https://doi.org/10.1039/B905558D

[3] F. Piccinno, R. Hischier, S. Seeger and C. Som. "From laboratory to industrial scale: a scale-up framework for chemical processes in life cycle assessment studies". *Journal of Cleaner Production,* 135 (2016): 1085-1097 https://doi.org/10.1016/j.jclepro.2016.06.164

[4] P. Kosky, R. Balmer, W. Keat and G. Wise 2020, "*Exploring Engineering: An Introduction to Engineering and Design*". Elsevier Science. 'ISBN 9780128150733.

[5] P. Holzapfel, J. Bunsen, I. Schmidt-Sierra, V. Bach and M. Finkbeiner. "Replacing location-based electricity consumption with market-based residual mixes in background data to avoid possible double counting: a quantitative analysis of effects and challenges". *The International Journal of Life Cycle Assessment,* 29 (2024): 1279-1289 https://doi.org/10.1007/s11367-024-02294-x

[6] S. Zhang, N. Ericsson, P.-A. Hansson, M. Sjödin and Å. Nordberg. "Life cycle assessment of an all-organic battery: Hotspots and opportunities for improvement". *Journal of Cleaner Production,* 337 (2022): 130454 https://doi.org/10.1016/j.jclepro.2022.130454

[7] S. Jungermann, N. Riegel, D. Müller, K. Meerholz and O. Nuyken. "Novel Photo-Cross-Linkable Hole-Transporting Polymers: Synthesis, Characterization, and Application in Organic Light Emitting Diodes". *Macromolecules,* 39 (2006): 8911-8919 https://doi.org/10.1021/ma060844o

[8] K. Schuller and K. Meerholz 2025. "Optimized Purification of 3-(((6-bromohexyl)oxy)methyl)-3-ethyloxetane" *Unpublished Results*, University of Cologne, Germany, Cologne.

[9] L. Plein, E. Gatzka and K. Meerholz 2025. "Synthesis of Photocrosslinkable Triphenylamine" *Unpublished Results*, University of Cologne, Germany, Cologne.

[10] L. Plein, L. Höfer and K. Meerholz 2025. "Photocrosslinkable Organic Cathode Material" *Unpublished Results*, University of Cologne, Germany, Cologne.

[11] J. Zhu, K. K. Hii and K. Hellgardt. "Toward a Green Generation of Oxidant on Demand: Practical Electrosynthesis of Ammonium Persulfate". *ACS Sustainable Chemistry & Engineering,* 4 (2016): 2027-2036 https://doi.org/10.1021/acssuschemeng.5b01372

[12] G. H. Imanzadeh, M. R. Zamanloo, Y. Mansoori and A. Khodayari. "Aqueous Media Oxidation of Alcohols with Ammonium Persulfate". *Chinese Journal of Chemistry,* 25 (2007): 836-838 https://doi.org/10.1002/cjoc.200790153

[13] M. O. Robeson 1965, "*Trimethylopropane"*. Celanese Corporation of America, United States of America, US3,183,274.

[14] P. Werle, M. Morawietz, S. Lundmark et al. 2008, "Alcohols, Polyhydric." in *Ullmann's Encyclopedia of Industrial Chemistry*. ISBN 9783527306732.

[15] P. Qiu, L. Wang, X. Jiang and B. Yang. "Synthesis of Diethyl Carbonate by the Combined Process of Transesterification with Distillation". *Energy & Fuels,* 26 (2012): 1254-1258 https://doi.org/10.1021/ef201732w

[16] D. B. Pattison. "Cyclic ethers made by pyrolysis of carbonate esters". *Journal of the American Chemical Society,* 79 (1957): 3455-3456

[17] Seinosuke Satoh; Yukio Tanigawa 1999, "*PROCESS FOR PRODUCING METHYLAL"*. Kurashiki (JP), United States, US 6,379,507 B1.

[18] E. Bernatek. " Methylal as a Solvent in Lithium Aluminium Hydride Reduction". *Acta Chemica Scandinavica,* 8 (1954): 874-875

[19] S. De, V. K. Aswal and S. Ramakrishnan. "Phenyl-Ring-Bearing Cationic Surfactants: Effect of Ring Location on the Micellar Structure". *Langmuir,* 26 (2010): 17882-17889 https://doi.org/10.1021/la1036053

[20] R. Adams and C. Marvel. "p-Bromophenol". *Org. Synth,* 1 (1921): 39

[21] M. Y. Lui, A. K. L. Yuen, A. F. Masters and T. Maschmeyer. "Masked N-Heterocyclic Carbene-Catalyzed Alkylation of Phenols with Organic Carbonates". *ChemSusChem,* 9 (2016): 2312-2316 https://doi.org/10.1002/cssc.201600493

[22] L. Sun, P. He, B. Xu, X. Xu and X. Wang. "Promoting the catalytic efficiency of a catalyst by a solvothermal method". *RSC Advances,* 3 (2013): 5819-5823 https://doi.org/10.1039/C3RA40489G

[23] C. J. Saint-Louis, D. J. Warner, K. S. Keane et al. "Photo-Electroswitchable Arylaminoazobenzenes". *The Journal of Organic Chemistry,* 86 (2021): 11341-11353 https://doi.org/10.1021/acs.joc.1c00763

[24] G. Lamanna, C. Faggi, F. Gasparrini et al. "Efficient Thia-Bridged Triarylamine Heterohelicenes: Synthesis, Resolution, and Absolute Configuration Determination". *Chemistry – A European Journal,* 14 (2008): 5747-5750 https://doi.org/10.1002/chem.200800705

[25] G. Koehler, M. Neumann, A. Harthun, F. Elsebach and K.-H. Bruehl 2001, "*Verfahren zur Herstellung von Alkalimetall- und Erdalkalimetall-Alkoholaten mit einer Wasserabtrennung durch Pervaporation"*. Evonik Operations GmbH, Germany, DE10158353A1.

[26] A. A. Kelkar, N. M. Patil and R. V. Chaudhari. "Copper-catalyzed amination of aryl halides: single-step synthesis of triarylamines". *Tetrahedron Letters,* 43 (2002): 7143-7146 https://doi.org/10.1016/S0040-4039(02)01708-2

[27] M. A. J. Huijbregts, Z. J. N. Steinmann, P. M. F. Elshout et al. 2016. "*ReCiPe 2016 : A harmonized life cycle impact assessment method at midpoint and endpoint level Report I: Characterization.*" Report No. RIVM Report 2016-0104 National Institute for Public Health and the Environment, <https://rivm.openrepository.com/entities/publication/3d19272d-3ee1-4d95-bbc7-3ad21f0a769e>.

[28] N. I. f. P. H. a. t. Environment. "Normalization scores ReCiPe 2016*"*. *National Institute for Public Health and the Environment*, (2024), https://www.rivm.nl/en/documents/normalization-scores-recipe-2016, accessed.

[29] Z. Liu, A. Yu and J. Y. Lee. "Synthesis and characterization of LiNi1− x− yCoxMnyO2 as the cathode materials of secondary lithium batteries". *Journal of Power Sources,* 81 (1999): 416-419 https://doi.org/10.1016/S0378-7753(99)00221-9

[30] E. Crenna, M. Gauch, R. Widmer, P. Wäger and R. Hischier. "Towards more flexibility and transparency in life cycle inventories for Lithium-ion batteries". *Resources, Conservation and Recycling,* 170 (2021): 105619 https://doi.org/10.1016/j.resconrec.2021.105619

[31] H. Yang, C. Wang, F. Hu et al. "Atomic-scale Pt clusters decorated on porous α-Ni(OH)2 nanowires as highly efficient electrocatalyst for hydrogen evolution reaction". *Science China Materials,* 60 (2017): 1121-1128 https://doi.org/10.1007/s40843-017-9035-8

[32] P. S. Molina. "InfoLink: 222 GWh more energy storage worldwide in 2025*"*. *EssNews powered by pv magazine*, (2025), https://www.ess-news.com/2025/02/25/infolink-222-gwh-more-energy-storage-worldwide-in-2025/, accessed 04/08/2026.

[33] G. Sirma and T. Wicke. "Competitive market for battery materials: Market leaders, technologies and cost analysis*"*. *Fraunhofer ISI*, (2025), https://www.isi.fraunhofer.de/en/blog/themen/batterie-update/batterie-materialien-kosten-technologien-markt-fuehrer.html, accessed 04/08/2026.
